# Adolescent mental health before, during, and after the COVID-19 pandemic in Iceland: a repeated, cross-sectional, population-based study

**DOI:** 10.1016/j.lanepe.2025.101301

**Published:** 2025-04-29

**Authors:** Erin Haskell, Berglind Sigmarsdottir, Ingibjorg Eva Thorisdottir, Audun Valborgarson, Elena Bonilla Aparicio, Olli Kiviruusu, Jaana Suvisaari, Zheng Chang, Eivind Ystrom, Agnieszka Butwicka, Bryndis Bjork Asgeirsdottir, Heiddis Bjork Valdimarsdottir, Inga Dora Sigfusdottir, John Philip Allegrante, Thorhildur Halldorsdottir

**Affiliations:** a Department of Psychology, Reykjavik University, Reykjavik, Iceland; b Planet Youth, Reykjavik, Iceland; c Department of Public Health Solutions, National Institute for Health and Welfare, Helsinki, Finland; d Department of Medical Epidemiology and Biostatistics, Karolinska Institute, Stockholm, Sweden; e Department of Psychology, Faculty of Social Sciences, University of Oslo, Oslo, Norway; f Division of Mental Health Services, R&D Department, Akershus University Hospital, Lørenskog, Norway; g Institute of Clinical Medicine, Faculty of Medicine, University of Oslo, Oslo, Norway; h Department of Biostatistics and Translational Medicine, Medical University of Lodz, Lodz, Poland; i Department of Population Health Science and Policy, Icahn School of Medicine at Mount Sinai, New York, NY, USA; j Department of Health Studies and Applied Educational Psychology, Teachers College, Columbia University, New York, NY, USA; k Centre of Public Health Sciences, University of Iceland, Reykjavik, Iceland

**Keywords:** Adolescents, Mental health, COVID-19, Gender differences, Screen use, Bioecological model

## Abstract

**Background:**

Adolescents' mental health declined during the COVID-19 pandemic, yet little is known about the long-term outcomes after the pandemic's declassification as a global health emergency (5 May 2023). This study examined changes in adolescent mental health in Iceland from 2016 to 2023, using a bioecological framework to identify risk and protective factors.

**Methods:**

Youth in Iceland surveys were administered nationwide to 13-15-year-olds in 2016, 2018, 2020, 2021, 2022 and 2023, with an average 75% response rate across the years. The surveys included measures on depressive symptoms, anxiety and hostility (Symptom Checklist-90), parental social support (Perceived Parental Support Scale), screen time, and stress/trauma exposure (Negative Life Events Scale). Stepwise-reduced mixed-effects models assessed the association of age, gender, time and risk/protective factors and their effect on mental health. Multiple imputation addressed missing data, and Bonferroni corrections adjusted for multiple testing.

**Findings:**

62,011 adolescents participated: 48.2% female (n = 29,890), 50.0% male (n = 31,002), 1.8% non-binary (n = 1119). Depressive symptoms showed signs of improvement post-pandemic (2023) compared to 2021 (β 0.19, 95% CI 0.13–0.24), yet remained higher than pre-pandemic levels (2016: β −0.38, 95% CI −0.44 to −0.33; 2018: β −0.26, 95% CI −0.31 to −0.20). Anxiety and hostility also increased and remained higher than pre-pandemic levels in 2023 (anxiety: 2016 β −0.29, 95% CI −0.35 to −0.24, 2018 β −0.20, 95% CI −0.26 to −0.15; hostility: 2016 β −0.26, 95% CI −0.31 to −0.20, 2018 β −0.12, 95% CI −0.18 to −0.07). Across all the models examining the predictors from diverse bioecological spheres, low parental social support, high social media use, and bad grades were consistently associated with poor mental health, regardless of mental health outcome and gender.

**Interpretation:**

The COVID-19 pandemic has had a serious and continuing negative effect on adolescents’ mental health. Targeted interventions are needed to address the increase in mental health problems during the COVID-19 pandemic, with a focus on enhancing parental support and managing screen use.

**Funding:**

Icelandic Research Fund (217612-051); 10.13039/501100004785NordForsk (147386).

**Research in context**

**Evidence before this study**

PubMed and Google Scholar were searched for peer-reviewed papers in English published from 1 January 2022 to 7 November 2024. In these databases, the terms “COVID-19″ or “coronavirus” were paired with “mental health”, “psych∗”, “depress∗”, “anx∗” or “hostility”. We combined these terms with “risk”, “protective”, “trauma”, “abuse”, “maltreatment”, “screen time”, “social media”, “gaming”, “social support”, “long-term” or “longitudinal”. The search was restricted to “adolescen∗”, “child” or “youth”. Additional relevant studies on youth mental health during COVID-19 were located in the identified papers.

To date, little is known about youth mental health outcomes following the declassification of COVID-19 as a global health emergency by the World Health Organization (WHO; 5 May 2023) (see Table S1 for overview of studies). The only existing population-based study tracking mental health of 13-20-year-old adolescents from 2015 to 2023 showed the initial increase in mental health problems during the pandemic has persisted.

Prior studies have identified female gender, family stressors, stress and trauma exposure, and screen time as risk factors for poor mental health outcomes among youth during the pandemic. Conversely, parental social support has consistently been reported as a protective factor. The existing findings are informative; however, their generalisability is limited due to small sample sizes, non-representative samples, and lack of pre-pandemic data. No study has integrated risk and protective factors across individual, family, and school/peer environments to determine which factors shaped adolescent mental throughout the COVID-19 pandemic.

**Added value of this study**

This study is, to the best of our knowledge, among the first to examine the long-term effects of COVID-19 on adolescent mental health at a population level after the WHO's declaration of the end of the pandemic, i.e. 5 May 2023. We found that adolescent mental health worsened during the pandemic, with the poorest outcomes occurring in 2022. Depressive symptoms decreased in 2023 compared to peaks in 2021 and 2022, particularly among girls. Anxiety and hostility increased in 2020 and remained high in 2023. Girls reported worse mental health scores overall but boys showed less post-pandemic recovery. Over the study period, parental social support decreased while arguments with parents, witnessing parental arguments, time spent gaming alone, social media use and serious accidents increased. Divorce and domestic violence rates remained relatively stable; however, peer sexual abuse increased during this time.

Leveraging the bioecological model, factors across environments contributed to mental health outcomes, with parental social support, social media use, and bad grades emerging as consistent predictors. Stress and trauma exposure were associated with mental health outcomes, although their contribution varied by outcome and gender. Social media use was associated with depressive symptoms, anxiety, and hostility, particularly among girls. For boys, serious arguments with parents, witnessing parental arguments, and speaking a non-Icelandic language at home were strongly linked to mental health problems.

**Implications of all the available evidence**

The collective findings consistently demonstrate a marked decline in adolescent mental health at the onset of the COVID-19 pandemic, characterised by increased depressive symptoms, anxiety, and hostility. However, research on adolescent mental health outcomes in the post-pandemic period remains limited. Available data suggest that the pandemic's negative effects persist beyond its immediate aftermath, though there are some indications of a gradual return to pre-pandemic levels. Evidence suggests that elements across environments influence adolescent mental health in times of stress, highlighting multiple opportunities for protective measures. Specifically, interventions should focus on enhancing parental social support, regulating screen time, and addressing trauma-related experiences, such as sexual abuse, at a societal level.

## Introduction

**Fig. 1:**
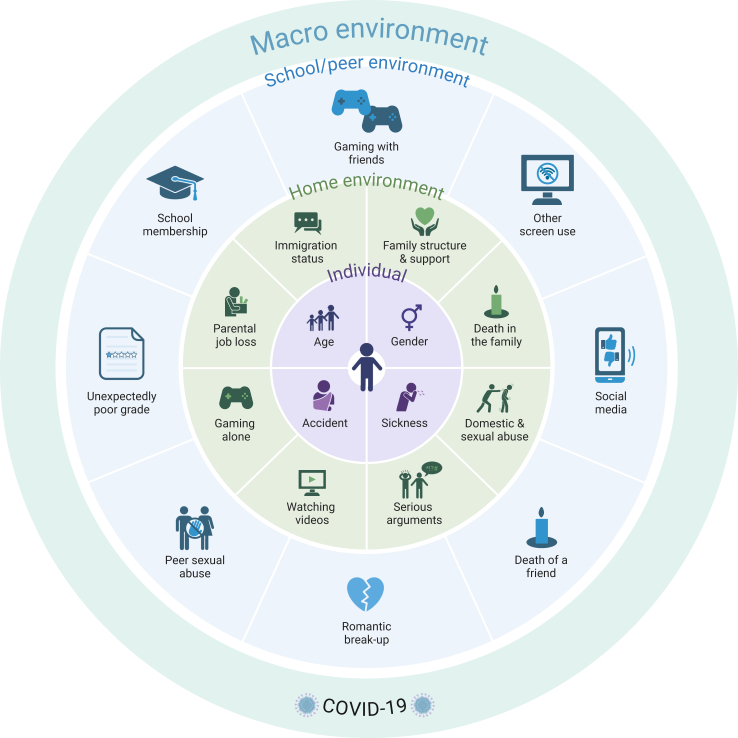
**Conceptual image of the bioecological model in the context of COVID-19, and depicting the variables utilised within this paper at each of the levels pertinent to an adolescent: individual, home and school/peer environments**. The central (purple) circle represents the ‘individual’ and includes variables of age, gender, and incidence of serious illness and accidents. The next (green) circle represents the immediate ‘home’ environment and includes variables relating to the parent/carer, activities in the home and home-related stresses and traumas. The next (light blue) circle includes variables relating to the ‘school/peer’ environment and includes academic- and peer-related stresses, traumas and activities. The elements of each environment can interact with each other, adjacent positioning does not indicate a direct interaction. The ‘macro’ scale of the bioecological model is represented by the light turquoise external circle, whereby all other bioecological spheres were affected by the COVID-19 pandemic and related restrictions, this is represented with the ‘year’ variable in our analysis. Created in BioRender. Haskell, E. (2025) licence details available at https://BioRender.com/f21j794.

The spread of SARS-CoV-2 (COVID-19) was defined as a global pandemic by the World Health Organisation (WHO) from 11 March 2020 to 5 May 2023. Fear and uncertainty surrounding the pandemic alongside social restrictions to control the virus's spread greatly impacted the daily life of all, including adolescents worldwide.^1^, ^2^, ^3^, ^4^, ^5^, ^6^ Existing literature indicates an increase in mental health problems during the early stages of the COVID-19 pandemic ^2^^,^^7^^,^^8^; however, few studies have examined the long-term adolescent mental health outcomes (see Appendix Table S1 for overview of studies).^9^, ^10^, ^11^, ^12^, ^13^, ^14^ To date, only one population-based study has tracked youth mental health from before the pandemic to after the WHO declassed COVID-19 as a global health emergency.^1^ The bioecological model defines the components of the ecosystem that can affect children's development.^15^ This model therefore provides an excellent framework to quantify the changes in an adolescent's life under the broad-reaching influence of the COVID-19 pandemic, how this affected mental health, and highlight areas receptive to prevention measures (Fig. 1).

In this repeated, cross-sectional, population-based study, we examined changes in the mental health of 13-15-year-olds in Iceland from 2016 to 2023. Expanding upon our previous work,^16^^,^^17^ we examined depressive symptoms, anxiety and hostility across time and applied the bioecological model to examine risk and protective factors associated with adolescent mental health, which have been shown to vary across the pandemic.^7^^,^^18^^,^^19^ These include parental social support, screen usage across various media types, and stressful or traumatic/significant life events. Given the well-documented gender differences in adolescent mental health, exacerbated during the pandemic,^1^^,^^2^^,^^16^ we examined outcomes overall and stratified by gender. Based on existing research,^1^ we hypothesised that post-pandemic (*i.e.* 2023) adolescent mental health would remain worse compared to pre-pandemic levels, both in terms of overall mental health scores and the proportion of youth with scores exceeding high threshold cutoffs. We also hypothesised that a decrease in parental social support,^20^^,^^21^ an increase in screen time^18^ and stress and trauma/significant life event exposure^19^^,^^22^ would occur during the pandemic and would be associated with poor mental health.

## Methods

### Survey design and participants

Data from the Youth in Iceland school surveys by the Icelandic Centre for Social Research and Analysis (ICSRA) was used. These nationwide surveys monitor factors associated with adolescent well-being. These surveys are modelled after comparative international studies (*e.g.,* European School Survey Project). Methodological details are available elsewhere,^17^^,^^23^ with additional details presented in the Appendix.

All schools offering grades 8–10 (13- to 15-year-olds) in Iceland participated. Surveys were distributed in February 2016 (n = 10,684), February 2018 (n = 10,563), November 2020 (n = 9169), March 2021 (n = 11,794), March 2022 (n = 10,263) and November 2023 (n = 9538). On average 136 schools participated (SD = 6.84), but some schools opted out in 2020 (n = 57) and 2023 (n = 56). The survey maintained a high response rate across the study period, averaging 74.59% (SD = 8.82) of all 13-15-year-olds in Iceland and closely matching the national age distribution (Appendix Figure S1). Survey administration overlapped with lockdowns in Iceland during 2020 and 2021, described in detail elsewhere.^17^

### Ethics approval

This study was approved by the National Bioethics Committee of Iceland (#21–038-V1) and the Icelandic Data Protection Authority and is reported in accordance with the STROBE guidelines (Appendix Table S2). Prior to survey administrations, caregivers received consent forms with an opt-out option. Adolescents received information that participation was voluntary, responses would be anonymous and confidential, and they could choose not to respond to any question or stop participation at any time.

### Procedures

Surveys were administered in class by teachers in paper format (2016–2018) or via an electronic link (2020–2023) and students responded anonymously. When paper-based, the students returned the questionnaires sealed in blank envelopes. Surveys were in Icelandic across all years and available in other languages across the study period. Number of surveys completed in a foreign language were as follows: English in 2020 (n = 156), 2022 (n = 257) and 2023 (n = 428) and Polish in 2022 (n = 85) and 2023 (n = 131).

### Measures

Age (13- *vs* 14- *vs* 15-years-old), self-reported gender identity (girl *vs* boy *vs* non-binary) and immigration differences (speaking only Icelandic at home *vs* other language spoken alone or with Icelandic) were assessed. To account for nesting within schools, school ID and capital area residency (residency within the capital area *vs* outside the capital area) were added as a random effect. Details on all variables, survey questions and response scales/options are presented in Appendix Table S3.

Depressive symptoms (10 items; range 10–40; Cronbach's α = 0.912), anxiety symptoms (5 items; range 5–25; α = 0.809) and hostility (5 items; range 5–25; α = 0.837) were assessed with the Symptom Checklist-90,^24^ based on participant's mood and behaviour in the preceding week. These subscales were used to capture both internalising and externalising problems. Composite scores were created for each dimension with higher scores indicating greater severity. In the absence of normative scores for the Icelandic version of the SCL-90-R, we adopted a conservative approach by defining high mental health problems with threshold cutoff scores at the top 5% of scores from the pre-pandemic year 2016, stratified by age and gender. This method was chosen to track changes in potentially clinically severe symptoms over time and is consistent with our prior publications.^16^^,^^17^

The Perceived Parental Support Scale^25^ measures how easily adolescents get social and emotional support from parents/caregivers. A composite score for parental social support was calculated, with higher scores indicating greater support (range 5–20; α = 0.883).

Participants reported time spent daily (range 0 to >6 hours) watching videos/films, playing video games alone or with friends, using social media, or other screen usage (*e.g*., homework). Changes in daily mean hours within each media type were analysed separately. Screen questions were not administered in 2021.

Exposure to 16 different stressors or traumatic/significant life events was assessed using the Negative Life Events Scale.^26^ A stressor was considered present if it occurred within the past 30 days and a traumatic/significant life event if experienced within the past year. Stressors included experiencing serious sickness, an unusually bad academic grade, a romantic breakup, having a serious argument with parents or witnessing a serious parental argument. Traumatic or significant life events included experiencing a serious accident, parental job loss, parental divorce/separation, witnessing or being subject to domestic violence, death of parent or sibling, death of a friend, and sexual abuse perpetrated by an adult or peer. These questions were not administered in 2021.

### Statistical analysis

Statistical analyses were conducted in R version 4.4.1; additional methods and R script are available in the Appendix. Expanding on our previous work on adolescent mental health during COVID-19,^16^^,^^17^ this paper examines these outcomes in the post-pandemic year 2023 compared to the height of the pandemic and preceding years. To determine general changes in mental health throughout the pandemic, base models examining the interactive effects of survey year (index: 2023), gender (index: female) and age (index: 13-years-old) were generated. The proportion of individuals who experienced the most severe (top 5% score standardised to 2016 levels in corresponding age and gender groups) mental health scores was calculated, and mixed-effects binomial models evaluated changes between years.

Composite scores for parental social support, screen usage and mental health were linearly transformed to be assessed by beta-distribution mixed-effect models in the glmmTMB package following a distribution assessment (detailed in Appendix).^27^^,^^28^ Stress- and trauma-related items were analysed using glmmTMB mixed-effects binomial models.

Additionally, separate models were built to analyse variables specific to each environment (‘individual’, ‘home’, and ‘school/peer’) and cumulatively (‘whole’ models) to address how different spheres influence adolescent mental health within the bioecological framework. These four models were run for depressive symptoms, anxiety and hostility, using a sample that combined all gender identities (mixed-gender model) as well as samples stratified by gender into male-only and female-only groups. Non-binary models were not individually generated due there being no non-binary option for gender pre-2020, and low sample size (Appendix Table S3). Models were generated using the buildglmmTMB^29^ function and a beta-distribution family.^28^ Stepwise model selection was employed to build robust models balancing goodness-of-fit and model complexity (full details available in the Appendix). Best-fit models were selected for each mental health outcome following model comparison between the base model (evaluating fixed-effects: year, age and gender) and the bioecological model levels (‘whole’, ‘individual’, ‘home’ and ‘school/peer’) using the Akaike and Bayesian Information Criteria (AIC, BIC), Log Likelihood and Deviance fit statistics. To gauge effect sizes, the recommended^30^ exponentiation of the beta coefficients, which are comparable to log-odds ratios, are presented in the tables.

Multiple imputation was used to address missing data (Appendix Table S4). A total of 115 analyses were conducted, and Bonferroni corrections set at *p* < 0.00043.

### Role of the funding source

The funders of the study had no role in study design, data collection, analysis, interpretation, or writing of the report.

## Results

Table 1 shows key characteristics and sample sizes across years. Proportions of age and gender distributions remained consistent (controlling for altered gender categories), but adolescents speaking non-Icelandic languages at home increased (χ^2^_(5, 62011)_ = 255.65, *p* < 0.0001). Residency differed (χ^2^_(5, 62011)_ = 1447.18, *p* < 0.0001), with fewer participants living outside the capital area in 2020 and 2023, due to aforementioned changes in school participation.

**Table 1:** Demographic characteristics of study participants, displayed for age group across the study period 2016–2023.

	Gender		Non-binary^a^	Language other than Icelandic spoken at home	Living in capital area
	Female	Male			
13 years old					
2016 (n = 3526)	1779 (50.5%)	1747 (49.5%)	–	634 (18.0%)	2198 (62.3%)
2018 (n = 3651)	1830 (50.1%)	1821 (49.9%)	–	682 (18.7%)	2284 (62.6%)
2020 (n = 3101)	1504 (48.5%)	1547 (49.9%)	50 (1.6%)	673 (21.7%)	2209 (71.2%)
2021 (n = 4055)	1895 (46.7%)	2057 (50.7%)	103 (2.5%)	959 (23.6%)	2600 (64.1%)
2022 (n = 3568)	1618 (45.3%)	1800 (50.4%)	150 (4.2%)	791 (22.2%)	2233 (62.6%)
2023 (n = 3315)	1579 (47.6%)	1662 (50.1%)	74 (2.2%)	813 (24.5%)	2739 (82.6%)
14 years old					
2016 (n = 3549)	1758 (49.5%)	1791 (50.5%)	–	587 (16.5%)	2214 (62.4%)
2018 (n = 3561)	1783 (50.1%)	1778 (49.9%)	–	641 (18.0%)	2157 (60.6%)
2020 (n = 3183)	1521 (47.8%)	1606 (50.5%)	56 (1.8%)	636 (20.0%)	2249 (70.7%)
2021 (n = 4003)	1905 (47.6%)	2000 (50.0%)	98 (2.4%)	834 (20.8%)	2486 (62.1%)
2022 (n = 3461)	1596 (46.1%)	1715 (49.6%)	150 (4.3%)	829 (24.0%)	2118 (61.2%)
2023 (n = 3184)	1491 (46.8%)	1596 (50.1%)	97 (3.0%)	799 (25.1%)	2572 (80.8%)
15 years old					
2016 (n = 3609)	1797 (49.8%)	1812 (50.2%)	–	568 (15.7%)	2207 (61.2%)
2018 (n = 3351)	1696 (50.6%)	1655 (49.4%)	–	606 (18.1%)	2065 (61.6%)
2020 (n = 2885)	1367 (47.4%)	1455 (50.4%)	63 (2.2%)	562 (19.5%)	2065 (71.6%)
2021 (n = 3736)	1791 (47.9%)	1863 (49.9%)	82 (2.2%)	780 (20.9%)	2371 (63.5%)
2022 (n = 3234)	1517 (46.9%)	1592 (49.2%)	125 (3.9%)	674 (20.8%)	1975 (61.1%)
2023 (n = 3039)	1463 (48.1%)	1505 (49.5%)	71 (2.3%)	735 (24.4%)	2475 (81.4%)

a The question response options for the question ‘What is your gender**?’** changed twice during the study period (Appendix Table S3). Ethnicity data were not collected in this study; however, language spoken at home was reported as a proxy for cultural background.

**Fig. 2:**
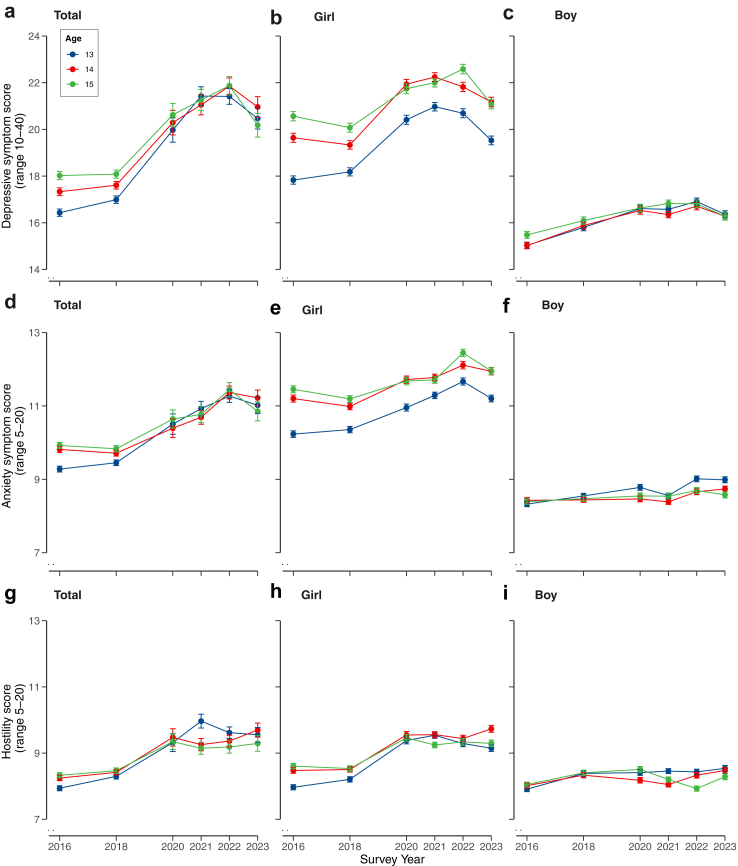
**Combined plot of mean mental health cumulative survey scores over the course of the survey period (2016**–**2023).** Participant age (13-, 14-, or 15-years-old) is represented by different colours. Graphs are segregated based on gender (Mixed gender, Girl, Boy). Depressive symptom scores range from 10 to 40 and are represented in graphs a–c, anxiety symptom scores range from 5 to 20 and are represented by graphs d–f, and hostility scores range from 5 to 20 and are represented by graphs g–i. Y-axis scales have been truncated to enable age group differences to be visualised. Error bars represent standard error. Y-axes scale origins are not set to 0, they have been truncated (as indicated by the .. symbol) to be adapted to the cumulative score range and enable age group differences to be visualised. Error bars represent standard error.

**Fig. 3:**
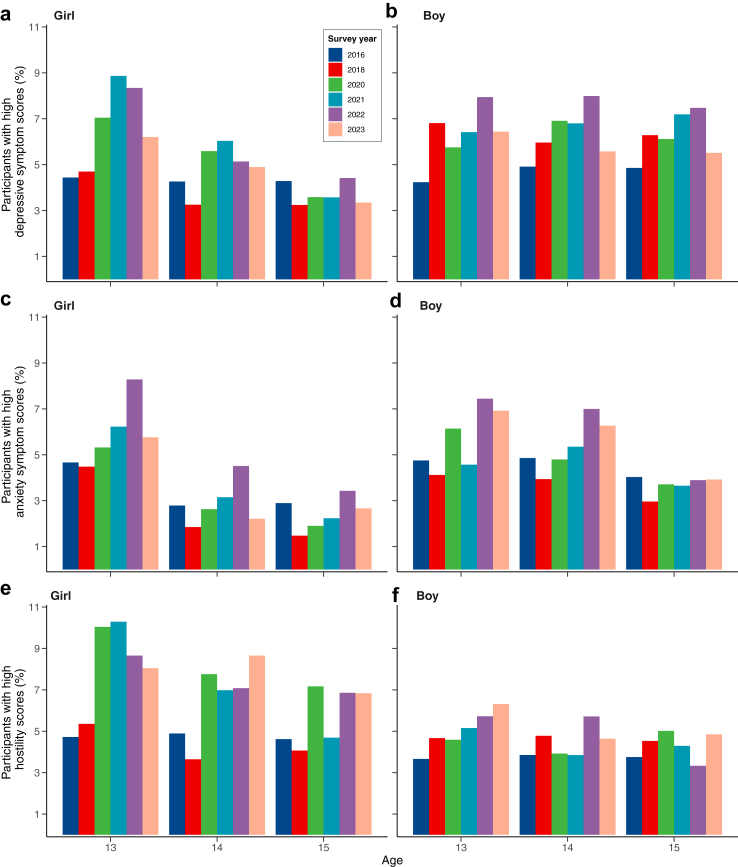
**Proportion of participants with high mental health symptom scores for 13-, 14- and 15-year-old adolescents across the study period (2016–2023)**. Figure shows the proportion of individuals with high depressive (plots a–b), anxiety (plots c–d) symptom and hostility (e–f) scores equal to or above those of the 5th centile determined separately for boys and girls for each age group in 2016 as a pre-pandemic baseline.

Fig. 2 shows mental health outcomes by age and gender over the years (Appendix Table S5). In the mixed-gender model (Appendix Table S6), pre-pandemic depressive symptoms were significantly lower (2016: β = −0.38, 95% CI = −0.44 to −0.33; 2018: β = −0.26, 95% CI = −0.31 to −0.20) than in 2023, while depressive symptoms in pandemic years were significantly higher, peaking in 2021 (β = 0.19, 95% CI = 0.13–0.24). Interaction terms revealed greater age-group differences in 2016 (β = 0.22, 95% CI = 0.14–0.30) and 2022 (β = −0.15, 95% CI = −0.23 to −0.07) compared to 2023. Among girls, a similar pattern was found (Appendix Table S6), while for boys, depressive symptoms in 2023 were lower compared to 2020 and 2022 but not 2021 (β = 0.13, 95% CI = 0.06–0.21). Severe depressive symptom scores in 2023 were not significantly different to other years, but in girls, significant differences were noted between 13- and 15-year-olds (Fig. 3, Appendix Table S7; OR = 0.52, 95% CI = 0.37–0.74).

Anxiety increased during the pandemic (Fig. 2). In the mixed-gender and girls' model, anxiety was higher in 2023 compared to pre-pandemic years, but not different from other pandemic years (Appendix Table S6). For boys, anxiety in 2016 was lower than 2023 (β = −0.25, 95% CI = −0.33 to −0.17) but not 2018 (β = −0.12, 95% CI = −0.19 to −0.04) or other years. Among girls, severe anxiety levels in 2023 were comparable to previous years but more prevalent in younger adolescents (Fig. 3; 14: OR = 0.37, 95% CI = 0.25–0.56; 15: OR = 0.45, 95% CI = 0.31–0.66). The proportion of boys with high anxiety in 2023 was higher compared to 2018 (OR = 0.64, 95% CI = 0.49–0.84), but not other years (Appendix Table S7).

In the mixed-gender model, hostility in 2016 was lower than 2023 (β = −0.26, 95% CI = −0.31 to −0.20) and 2018 (β = −0.12, 95% CI = −0.18 to −0.07), but remained comparable to other pandemic years. Interaction terms suggest dynamic age-related effects on hostility over time (Appendix Table S6). For boys, hostility was significantly lower in 2016 compared to 2023 (β = −0.15, 95% CI = −0.23 to −0.07), with no other significant terms. Among girls, high hostility in 2016 was less prevalent than in 2023 (OR = 0.56, 95% CI = 0.42–0.75), with no other significant differences, nor in the model for boys.

**Fig. 4:**
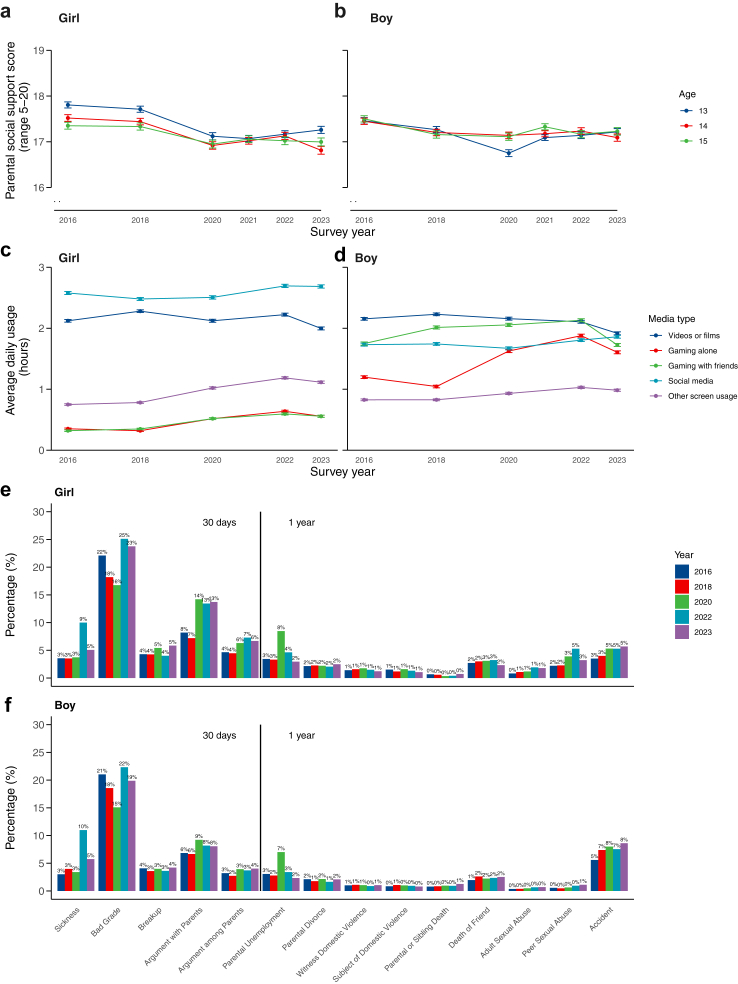
**Combined plot showing the changes in survey responses on parental social support, screen use and stress and trauma across the study period**. Plots a–b show mean cumulative survey scores (range 5–20) for parental social support for girls and boys respectively, higher scores indicate better perceived levels of support, note that Y-axes scale origins are not set to 0, they have been truncated (as indicated by the . symbol). Plots c–d show screen usage (mean hours per day, range 0 h–6+ hours), segregated by media type: watching videos, playing video games alone or with friends, social media use and offline/other screen use, for girls and boys respectively. Plots e–f show the proportion of survey participants reporting an experience of specific stressors and traumas across the study period (response options were binary), for girls and boys respectively. Responses are split by reports within the last 30 days for stressful experiences, and in the last year for traumatic experiences. Screen usage and stress and trauma related questions were not asked in 2021. Error bars represent standard error.

Fig. 4 shows trends in parental social support, screen time, and stress/trauma throughout the pandemic. Parental social support in pre-pandemic levels was significantly higher than 2023 (2016: β = 0.22, 95% CI = 0.15–0.30; 2018: β = 0.19, 95% CI = 0.11–0.27), but comparable to other pandemic years for girls. For boys, parental support was higher in 2023 compared to 2020 (β = −0.20, 95% CI = −0.28 to −0.12), with no other significant differences.

Time spent gaming alone increased during COVID-19 and remained higher than pre-pandemic levels for girls and boys (Fig. 4). This pattern was also seen in girls' gaming with friends, while boys' gaming with friends rose during the pandemic but decreased by 2023 (Appendix Table S8). Boys’ video watching steadily declined since 2018, reaching lower levels in 2023 (β = 0.26, 95% CI = 0.17–0.34), while no significant changes occurred for girls. For girls, social media use in 2018 was lower than in 2023 (β = −0.18, 95% CI = −0.27 to −0.09), with no other notable differences. Social media use was relatively stable across time among boys. A few interactions were noted between age and screen time (expanded upon in Appendix results and Table S8).

Across all stress/trauma models, occurrence of recent serious sickness was highest in 2022, but incidence in 2023 remained higher compared to other years (Appendix Table S9). Reports of bad grades were fewer in 2018 (OR = 0.85, 95% CI = 0.79–0.91) and 2020 (OR = 0.69, 95% CI = 0.64–0.75), compared to 2023, but lower than the 2022 peak (OR = 1.18, 95% CI = 1.11–1.27). Arguments with parents and witnessing parental arguments increased in 2020 and stayed high throughout the pandemic. Parental job loss peaked in 2020 (OR = 3.11, 95% CI = 2.68–3.60) but returned to pre-pandemic levels by 2023. Reports of serious accidents also increased. Specific to girls, sexual abuse, by both adults and peers, was higher during the pandemic (Fig. 4). No significant changes were found in parental divorce, domestic violence, or death of a parent, sibling, or friend over time.

Models incorporating all bioecological spheres (‘whole’ models) had superior model fit compared to simpler models, across mental health measures in mixed-gender and gender-stratified analyses. Factors associated with poor mental health across all models included low parental support, high social media use, and an unexpectedly bad grade (Appendix Table S10). This was also true for the models with the reference year set to 2016, with romantic breakup and time spent watching videos additionally included in all models (Appendix Table S11). Stress/trauma factors generally had the highest estimates, but also variance. Parental divorce and domestic violence were not retained in any model. The random term for capital area residency was dropped from all models; school membership was also dropped, except for depression and hostility models for girls.

In the bioecological model for girls, peer sexual abuse (β = 0.65, 95% CI = 0.57–0.73) had the strongest association with higher depressive symptoms, followed by arguments with parents (β = 0.46, 95% CI = 0.41–0.51), receiving a bad grade (β = 0.33, 95% CI = 0.29–0.37), and video watching and social media use (both β = 0.09, 95% CI = 0.08–0.10). Two variables interacted with survey year: parental social support was a stronger protective factor in all years except 2023 and 2022 (β = −0.02, 95% CI = −0.03 to 0.00), and romantic breakups had a stronger positive association with depressive symptoms in 2016 compared to 2023 (β = 0.35, 95% CI = 0.20–0.51).

Boys' depressive symptoms were positively associated with serious arguments with parents (β = 0.43, 95% CI = 0.37–0.48), social media use (β = 0.02, 95% CI = 0.01–0.03), and video watching (β = 0.08, 95% CI = 0.07–0.08). Unique to boys, witnessing parental arguments (β = 0.21, 95% CI = 0.13–0.29), experiencing a serious accident (β = 0.20, 95% CI = 0.14–0.25), speaking a non-Icelandic language at home (β = 0.08, 95% CI = 0.04–0.11), and gaming alone (β = 0.04, 95% CI = 0.03–0.04) were linked to higher depressive scores.

In the anxiety models (Appendix Table S10), most predictors were retained for girls and boys. Peer sexual abuse (β = 0.47, 95% CI = 0.38–0.55), recent serious sickness (β = 0.41, 95% CI = 0.34–0.48), bad grades (β = 0.30, 95% CI = 0.26–0.33), and serious accidents (β = 0.25, 95% CI = 0.18–0.32) were the largest estimates for girls. Social media (β = 0.07, 95% CI = 0.06–0.08), other screen use (β = 0.07, 95% CI = 0.05–0.08), video watching (β = 0.06, 95% CI = 0.05–0.07), and gaming with friends (β = 0.02, 95% CI = 0.00–0.03) were also linked to higher anxiety in girls. Parental social support (β = −0.05, 95% CI = −0.06 to −0.04) and speaking a non-Icelandic language at home (β = −0.10, 95% CI = −0.14 to −0.06) were associated with lower anxiety in girls.

For boys, year was not retained in the model. The largest estimates of anxiety were recent serious sickness (β = 0.34, 95% CI = 0.27–0.40) and death of a friend (β = 0.33, 95% CI = 0.27–0.43). Increased time watching videos (β = 0.06, 95% CI = 0.05–0.06), social media use (β = 0.03, 95% CI = 0.02–0.04), other screen use (β = 0.03, 95% CI = 0.01–0.04), and gaming with friends (β = 0.02, 95% CI = 0.01–0.02) were also linked to higher anxiety.

The strongest predictor of hostility was serious arguments with parents for girls (β = 0.78, 95% CI = 0.73–0.84) and boys (β = 0.82, 95% CI = 0.76–0.88). Following this, hostility was strongly linked to romantic breakups (girls:β = 0.38, 95% CI = 0.33–0.43; boys:β = 0.39, 95% CI = 0.29–0.39) and bad grades (girls:β = 0.33, 95% CI = 0.25–0.42; boys:β = 0.28, 95% CI = 0.24–0.31). Hostility in girls was further strongly linked to peer sexual abuse (β = 0.48, 95% CI = 0.39–0.57) and social media use (β = 0.11, 95% CI = 0.10–0.12). Higher parental support (β = −0.09, 95% CI = −0.10 to −0.09) and speaking a non-Icelandic language at home (β = −0.08, 95% CI = −0.12 to −0.04) were associated with lower hostility.

Other factors strongly influencing boys’ hostility included death of a friend (β = 0.29, 95% CI = 0.19–0.39), serious accidents (β = 0.23, 95% CI = 0.17–0.29), parental unemployment (β = 0.16, 95% CI = 0.08–0.23), social media use (β = 0.08, 95% CI = 0.06–0.10), video watching (β = 0.04, 95% CI = 0.03–0.05), and gaming alone (β = 0.02, 95% CI = 0.01–0.03). The bioecological, base, threshold models and were also run with the reference year set to 2016 to enable comparison with previous work (Appendix Tables S11–S13, respectively).

## Discussion

This study is, to the best of our knowledge, the first to examine changes in adolescent mental health before, throughout and following the COVID-19 pandemic using a bioecological approach to understand how individual, home, and school/peer environments shaped mental health. Partly in line with our hypothesis, depressive symptoms showed a downward trend towards pre-pandemic levels in 2023, especially among girls. In contrast, anxiety and hostility increased during the pandemic and showed limited signs of recovery post-pandemic. While the proportion of youth with severely high mental health problems in 2023 was not significantly different from other years, there were significant increases throughout the pandemic in the models indexed to 2016, particularly for younger adolescents. Despite some reduction towards pre-pandemic levels, these findings suggest that the widespread changes and uncertainty experienced during the pandemic have had lingering effects for girls and boys, particularly regarding hostility.

There were notable age-related differences, primarily reflected in a wider gap between mental health scores of 13- and 15-year-olds in the pre-pandemic years compared to 2023. This is mirrored in the growing proportion of 13-year-olds reporting severe mental health scores relative to 2016. This suggests that younger children may have experienced greater deviations in their expected, pre-pandemic mental health trajectories, as seen in related studies.^1^^,^^7^ Given the consistency across age groups, there would be value in investigating changes over time for each birth-year cohort to explore the influence of age of exposure to the pandemic-related stresses.

The pandemic's impact varied by gender, with depressive symptoms and anxiety sharply rising among girls but demonstrating some recovery by 2023. Meanwhile, boys showed little to no improvement in mental health throughout the pandemic. This suggests adolescent girls' mental health is more responsive to environmental influences, potentially due to both biological^31^^,^^32^ and societal factors, some of which are captured in our bioecological framework. These findings highlight that girls are at higher risk for mental health problems in times of stress, but the long-term trajectory of boys' mental health should be closely monitored following easing of stress.

More broadly, the bioecological framework demonstrated that elements across environments influenced adolescent mental health throughout the pandemic, highlighting multiple opportunities for protective measures. Within the home, decreased parental social support coincided with heightened arguments with parents during the pandemic, persisting into 2023, exacerbating depressive symptoms, anxiety, and particularly hostility. The peak in 2020 likely stems from families spending more time together in a stressful environment.^21^^,^^33^ Increased family time and disrupted routines during lockdowns may have led to friction and burnout, revealing gaps in perceived support.^20^ Studies also indicate that stress during adolescence may drive a desire for independence and risk taking,^34^ which may have contributed to the rise in conflicts. This finding underscores the importance of maintaining quality family support to protect adolescent mental health under stress.

Contrary to prior research,^18^ we found surprisingly few changes in screen use throughout the pandemic. Gaming alone and with friends increased, particularly gaming alone among boys in 2020, and was linked to higher hostility and depressive symptoms, while gaming with friends was associated with higher anxiety. Social media use remained relatively stable and was the most common screen activity for girls, with high use consistently linked to poorer mental health in girls and boys. A previous population-based study conducted in Iceland highlights the relationship between passive social media use and poorer mental health outcomes in both boys and girls.^35^ While viewing time spent on different media types is informative, it is crucial to explore how adolescents engage with online interactions.^36^

Also contrary to prior findings,^19^^,^^37^ we found no changes in domestic violence (witnessed or experienced) during COVID-19. This may be attributed to Iceland's less restrictive lockdowns and high levels of gender equality. Girls reported more stressful/traumatic events, including sexual abuse, possibly linked to shifting societal views on consent following the resurgence of the #MeToo movement in Iceland in 2021. Alternatively, increases in peer sexual abuse and accidents since 2020 may reflect heightened risk-taking behaviours, triggered by social restrictions during adolescence. Studies in animals and humans show that social isolation and stress during puberty can lead to earlier sexual activity and greater risk-taking.^34^ Future studies are needed to monitor such risky behaviours and potential consequences in adolescents in the wake of the pandemic.

At a societal level, all government-mandated restrictions in Iceland were lifted in June 2021. In December 2021, COVID-19 infections surged, peaking in February 2022 at around 10,300 per 100,000 individuals, coinciding with the administration of the 2022 Youth in Iceland survey. There was a 40% increase in excess deaths in March 2022, while registry data states 13-17-year-olds had the highest cumulative infection rate. This is reflected in the high reports of serious sickness and likely contributed to 2022 being the worst year for adolescent mental health. While there were no significant changes in reports of parental, sibling, or friend deaths, the lack of data on extended family deaths may limit conclusions. However, attributing the decline in mental health solely to increased COVID-19 infections oversimplifies the issue, given the preceding years of worsening mental health and ongoing uncertainty surrounding the pandemic and other global crises.

This study has several notable strengths, including its design, sample size, and population reach. However, self-reported mental health problems may not fully capture clinically relevant issues, though previous work suggests a link between self-reported depressive symptoms and visits to paediatric psychiatrists and child psychologists.^38^ Measures of hostility or irritability can be part of the manifestation of depression in adolescents, however the correlation between these measures was only moderate in this sample (Appendix Table S14). There were some variations in survey questions over time; notably, the 2020 and 2021 surveys omitted questions on peer support, household status, and additionally screentime and trauma in 2021 (Appendix Table S3). Social media usage and the (Other screen usage) category are broad and may overlook important nuances in platforms and apps. Differences in survey administration methods (offline *vs* online) and timing may have influenced pre- and post-pandemic ratings. This study did not include measures of race and ethnicity, other than language spoken at home, restricting our ability to analyse potential disparities related to these factors. Finally, the nature of cross-sectional, non-longitudinal, data means that the ability to inform causation is limited.

In conclusion, this study is, based on our current understanding, one of the first to examine adolescent mental health at a population-level before, during, and following the pandemic with repeated assessments. Our findings emphasise the lasting mental health burden on adolescents who experienced significant social and academic disruptions during this critical period. While mental health improved to some degree in 2023, particularly for girls, no measures returned to pre-pandemic levels. Positively, the bioecological framework highlights multiple opportunities for targeted interventions to support adolescent mental health.

## Contributors

EH and TH conceived the research question and design. AB, JS, OK, ZC, and EY also contributed to the conceptualisation of this study. IDS, BBA, HBV, JPA and IET, along with the personnel at Icelandic Centre for Social Research and Analysis, identified the measures used in the surveys at all timepoints and coordinated the administration to all schools in Iceland. EH ran all analyses, with support from BS and TH. AV assisted with developing and interpreting the statistical analyses. EH and TH wrote the first draft of the Article. EH, BS and TH accessed the data in the study and verified the underlying data. BS and EBA completed the literature review. All authors read and approved the final manuscript. All authors confirm they had full access to all the data in the study and accept responsibility for the decision to submit for publication.

## Data sharing statement

Individual participant data that underlie the results reported in this Article (after de-identification) and a data dictionary defining each variable will be made available for researchers who provide a methodologically sound proposal. Of note, the execution of such a proposal requires approval by the Icelandic Bioethics Committee. Proposals should be directed to thorhildurh@ru.is. To gain access, those requesting access to the data will need to sign a data access agreement.

## Declaration of interests

IET is a staff member at Planet Youth, a youth substance use prevention service organisation that is distributed globally through sale of the Planet Youth Guidance Program, which is based on the Icelandic Prevention Model, from which she receives a salary. All other authors declare no competing interests.

## Appendix A Supplementary data

Supplementary Material

IcelandicAbstract

Fig 1_Biorender_PublicationLicense

## References

[1] Kiviruusu O., Ranta K., Lindgren M. (2024). Mental health after the COVID-19 pandemic among Finnish youth: a repeated, cross-sectional, population-based study. Lancet Psychiatry.

[2] Samji H., Wu J., Ladak A. (2022). Review: mental health impacts of the COVID-19 pandemic on children and youth – a systematic review. Child Adolesc Ment Health.

[3] McGorry P.D., Mei C., Dalal N. (2024). The Lancet psychiatry commission on youth mental health - the Lancet psychiatry. Lancet Psychiatry Comm.

[4] Hakulinen C., Komulainen K. (2024). Perspectives on adolescent mental health after the COVID-19 pandemic. Lancet Psychiatry.

[5] Orben A., Tomova L., Blakemore S.-J. (2020). The effects of social deprivation on adolescent development and mental health. Lancet Child Adolesc Health.

[6] Hafstad G.S., Augusti E.-M. (2021). A lost generation? COVID-19 and adolescent mental health. Lancet Psychiatry.

[7] Panchal U., Salazar de Pablo G., Franco M. (2023). The impact of COVID-19 lockdown on child and adolescent mental health: systematic review. Eur Child Adolesc Psychiatry.

[8] Madigan S., Racine N., Vaillancourt T. (2023). Changes in depression and anxiety among children and adolescents from before to during the COVID-19 pandemic: a systematic review and meta-analysis. JAMA Pediatr.

[9] Zoellner F., Erhart M., Napp A.-K. (2024). Risk and protective factors for mental health problems in children and adolescents during the COVID-19 pandemic: results of the longitudinal COPSY study. Eur Child Adolesc Psychiatry.

[10] Shoshani A. (2024). Longitudinal changes in children's and adolescents' mental health and well-being and associated protective factors during the COVID-19 pandemic. Psychol Trauma Theory Res Pract Policy.

[11] Zijlmans J., Tieskens J.M., van Oers H.A. (2023). The effects of COVID-19 on child mental health: biannual assessments up to April 2022 in a clinical and two general population samples. JCPP Adv.

[12] Barbieri V., Wiedermann C.J., Piccoliori G. (2023). Evolution of youth's mental health and quality of life during the COVID-19 pandemic in south Tyrol, Italy: comparison of two representative surveys. Children.

[13] Park J.L., McArthur B.A., Plamondon A. (2024). The course of children's mental health symptoms during and beyond the COVID-19 pandemic. Psychol Med.

[14] Bhandari N., Gupta S. (2024). Trends in mental wellbeing of US children, 2019–2022: erosion of mental health continued in 2022. Int J Environ Res Public Health.

[15] Bronfenbrenner U., Evans G.W. (2000). Developmental science in the 21 Century: emerging questions, theoretical models, research designs and empirical findings. Soc Dev.

[16] Thorisdottir I.E., Agustsson G., Oskarsdottir S.Y. (2023). Effect of the COVID-19 pandemic on adolescent mental health and substance use up to March, 2022, in Iceland: a repeated, cross-sectional, population-based study. Lancet Child Adolesc Health.

[17] Thorisdottir I.E., Asgeirsdottir B.B., Kristjansson A.L. (2021). Depressive symptoms, mental wellbeing, and substance use among adolescents before and during the COVID-19 pandemic in Iceland: a longitudinal, population-based study. Lancet Psychiatry.

[18] Madigan S., Eirich R., Pador P., McArthur B.A., Neville R.D. (2022). Assessment of changes in child and adolescent screen time during the COVID-19 pandemic: a systematic review and meta-analysis. JAMA Pediatr.

[19] Calvano C., Engelke L., Di Bella J., Kindermann J., Renneberg B., Winter S.M. (2022). Families in the COVID-19 pandemic: parental stress, parent mental health and the occurrence of adverse childhood experiences—results of a representative survey in Germany. Eur Child Adolesc Psychiatry.

[20] Cohodes E.M., McCauley S., Gee D.G. (2021). Parental buffering of stress in the time of COVID-19: family-level factors may moderate the association between pandemic-related stress and youth symptomatology. Res Child Adolesc Psychopathol.

[21] Moss S.J., Stelfox M., McArthur E. (2024). Social factors associated with self-reported changes in mental health symptoms among youth in the COVID-19 pandemic: a cross-sectional survey. BMC Public Health.

[22] Augusti E.-M., Myhre M.C., Wentzel-Larsen T., Hafstad G.S. (2023). Violence and sexual abuse rates before and during the Covid-19 pandemic: a prospective population-based study on Norwegian youth. Child Abuse Negl.

[23] Kristjansson A.L., Sigfusson J., Sigfusdottir I.D., Allegrante J.P. (2013). Data collection procedures for school-based surveys among adolescents: the youth in Europe study. J Sch Health.

[24] Derogatis L.R., Unger R. (2010).

[25] Thorlindsson T., Bjarnason T. (1998). Modeling durkheim on the micro level: a study of youth suicidality. Am Sociol Rev.

[26] Gudjonsson G., Sigurdsson J.F., Sigfusdottir I.D. (2009). False confessions among 15- and 16-year-olds in compulsory education and the relationship with adverse life events. J Forensic Psychiatry Psychol.

[27] Delignette-Muller M.L., Dutang C. (2015). Fitdistrplus: an R package for fitting distributions. J Stat Softw.

[28] Brooks M.E., Kristensen K., van Benthem K.J. (2017). glmmTMB balances speed and flexibility among packages for zero-inflated generalized linear mixed modeling. R J.

[29] Voeten C.C. (2023). Buildmer: stepwise elimination and term reordering for mixed-effects regression. https://cran.r-project.org/web/packages/buildmer/index.html.

[30] Geissinger E.A., Khoo C.L.L., Richmond I.C., Faulkner S.J.M., Schneider D.C. (2022). A case for beta regression in the natural sciences. Ecosphere.

[31] Prince C., Joinson C., Kwong A.S.F., Fraser A., Heron J. (2023). The relationship between timing of onset of menarche and depressive symptoms from adolescence to adulthood. Epidemiol Psychiatr Sci.

[32] Dehestani N., Vijayakumar N., Ball G., Mansour L.S., Whittle S., Silk T.J. (2024). “Puberty age gap”: new method of assessing pubertal timing and its association with mental health problems. Mol Psychiatry.

[33] Spinelli M., Lionetti F., Pastore M., Fasolo M. (2020). Parents' stress and children's psychological problems in families facing the COVID-19 outbreak in Italy. Front Psychol.

[34] Blakemore S.-J., Mills K.L. (2014). Is adolescence a sensitive period for sociocultural processing?. Annu Rev Psychol.

[35] Thorisdottir I.E., Sigurvinsdottir R., Asgeirsdottir B.B., Allegrante J.P., Sigfusdottir I.D. (2019). Active and passive social media use and symptoms of anxiety and depressed mood among Icelandic adolescents. Cyberpsychol Behav Soc Netw.

[36] Odgers C.L., Jensen M.R. (2020). Annual research review: adolescent mental health in the digital age: facts, fears, and future directions. J Child Psychol Psychiatry.

[37] Ravindran S., Shah M. (2023). Unintended consequences of lockdowns, COVID-19 and the shadow pandemic in India. Nat Hum Behav.

[38] Sigfusdottir I.D., Asgeirsdottir B.B., Sigurdsson J.F., Gudjonsson G.H. (2008). Trends in depressive symptoms, anxiety symptoms and visits to healthcare specialists: a national study among Icelandic adolescents. Scand J Public Health.

