# Supplementary material for: Adolescent mental health before, during, and after the COVID-19 pandemic in Iceland: a repeated, cross-sectional, population-based study
Source: Lancet Reg Health Eur. 2025 Apr 29;53:101301. doi: 10.1016/j.lanepe.2025.101301 (PMC12432985; doi:10.1016/j.lanepe.2025.101301)
Supplement: Supplementary Material [file mmc1.docx]

Supplementary appendix 1

To accompany: Bioecological approach towards predicting adolescent mental health during COVID-19 in Iceland (2016-2023): a repeated, cross-sectional, population-based study

[SUPPLEMENTARY METHODS 3](#_Toc190175614)

[Study design 3](#_Toc190175615)

[Measures 3](#_Toc190175616)

[Statistical analysis 4](#_Toc190175617)

[Model refinement 4](#_Toc190175618)

[Missing data 4](#_Toc190175619)

[SUPPLEMENTARY RESULTS 5](#_Toc190175620)

[Screen time model interactions 5](#_Toc190175621)

[SUPPLEMENTARY TABLES 6](#_Toc190175622)

[Table S1. Literature review paper summary. 6](#_Toc190175623)

[Table S2. STROBE Statement 8](#_Toc190175624)

[Table S3. Details of survey question phrasing and response options 9](#_Toc190175625)

[Table S4. The extent of missing data 11](#_Toc190175626)

[Table S5. Depressive symptoms, anxiety symptoms, and hostility scores for each gender-age group pairing for each year of the survey administration (2016-2018). 12](#_Toc190175627)

[Table S6. Base model predictions for adolescent mental health outcomes (2023 Reference Year). Fixed effects of survey year (indexed to 2023), age, gender and interactions on mental health outcomes (depressive symptom scores, anxiety symptom scores and hostility scores), 14](#_Toc190175628)

[Table S7. Model predictions for proportions of adolescents with high mental health scores 16](#_Toc190175629)

[Table S8. Model predictions for screen time across the study period (2023 Reference Year). 18](#_Toc190175630)

[Table S9. Model predictions for stress/trauma event incidence throughout the study period. 21](#_Toc190175631)

[Table S10. Bioecological model predictions for adolescent mental health outcomes (2023 Reference Year). 24](#_Toc190175632)

[Table S11. Bioecological model predictions for adolescent mental health outcomes (2016 Reference Year). 28](#_Toc190175633)

[Table S12. Base model predictions for adolescent mental health outcomes (2016 Reference Year) 33](#_Toc190175634)

[Table S13. Model predictions for proportions of adolescents with high mental health scores (2016 Reference Year). 36](#_Toc190175635)

[Table S14: Spearman rank correlation matrix showing the relationship between the mental health symptom cumulative scores. 38](#_Toc190175636)

[SUPPLEMENTARY FIGURES 39](#_Toc190175637)

[Figure S1. Survey response rates 39](#_Toc190175638)

[R-script 40](#_Toc190175639)

[1. Create a dummy dataset modelled on the summary statistics of variables from Youth in Iceland study 40](#_Toc190175640)

[2. Cumulative survey score data analysis (‘base’ models in paper) 42](#_Toc190175641)

[Step 1: Converting cumulative/composite mental health survey scores to a beta scale for statistical analysis 42](#_Toc190175642)

[Step 2: Perform beta mixed-effects regression for mental health composite scores (referred to as ‘base models’ in the paper) 44](#_Toc190175643)

[Step 3: Convert model estimates back to original survey score scale 46](#_Toc190175644)

[3. Model building and stepwise reduction (as for the ‘bioecological models’ in the paper) 48](#_Toc190175645)

[Step 1: Define starting model formula 48](#_Toc190175646)

[Step 2: Use buildglmmTMB to build a formula using all the terms above, and order them to make the model that describes the data the best (log likelihood, LRT), and avoiding non-convergence 48](#_Toc190175647)

[Step 3: Use build glmmTMB to refine the model based on the BIC criterion, in a backwards direction, removing terms from the tail end of the formula first. 48](#_Toc190175648)

[Step 4: Run the model using the resulting formula on the resulting formula 49](#_Toc190175649)

[Step 5: Examine output 49](#_Toc190175650)

[4. High threshold mental health score data analysis 50](#_Toc190175651)

[Step 1: Calculate pre-COVID 2016 baseline mental health cumulative scores: what is the top 5% threshold for each respective age and gender grouping? 50](#_Toc190175652)

[Step 2: Calculate the number of individuals reporting the highest scores >95% within the reference year, for each age and gender grouping 50](#_Toc190175653)

[Step 3: Create binary variable (1= above 95th threshold, 0 = below 95th threshold) to determine proportion of individuals reporting extreme >95th centile mental health scores 51](#_Toc190175654)

[Step 4: Test for changes with binomial mixed effect models 52](#_Toc190175655)

[REFERENCES 52](#_Toc190175656)

# SUPPLEMENTARY METHODS

## Study design

The Youth in Iceland surveys, conducted by the Icelandic Centre for Social Research and Analysis (ICSRA), aim to identify risk and protective factors affecting adolescent well-being and substance use across individual, family, peer, school, and community levels. These surveys are based on international studies like the European School Survey Project and the U.S.-based Monitoring the Future Study. ICSRA researchers select survey measures annually, with most remaining consistent over time. New measures are added when necessary, through collaboration with various stakeholders, including the Directorate of Health, the Ministry of Education, the Ministry of Health, municipalities, and non-governmental organisations. All students enrolled in grades 8-10 (13- to 15-years-old) in Iceland were invited to participate in the study. No incentives were offered to adolescents or their families for participating in the study. Before completing the survey, adolescents were provided with clear, written information about the survey. The introduction to the questionnaire explicitly stated that participation was voluntary; responses would be anonymous and confidential, ensuring privacy; and participants could choose not to respond to any question or stop participation at any time. By proceeding with the questionnaire, adolescents provided implied consent.

Traditionally, the Youth in Iceland surveys were administered biennially to 13-15-year-olds (every even-numbered year). However, during the pandemic, the surveys were conducted annually due to heightened concerns about youth mental health. For these additional administrations, efforts were made to shorten the survey to minimise completion time, reduce disruption to teaching, and maintain high participation rates. As a result, the screen time and stressor/trauma questions were omitted.

## Measures

The Symptom Checklist-90 (SCL-90)^1^ is a widely used self-report questionnaire, measuring symptoms of various mental health problems. In this study, the depression, anxiety and hostility subscales of the SCL-90 were included to capture both internalising and externalising problems. The measure of hostility assesses irritability, tantrums/outbursts, argumentativeness and outward expression of anger (*e.g.* breaking or throwing things, screaming). Irritability has especially been shown to be a transdiagnostic symptom associated with oppositional defiant disorder, as well as depression and anxiety. By including these subscales, we aimed to capture a wider range of behavioural and emotional responses to the pandemic and to examine how these might differ by gender. Traditionally, the SCL-90 has been used to assess various psychopathological dimensions, but studies have demonstrated its effectiveness in detecting psychopathology among adolescents.^2–4^ Previous research has also shown that the depression and anxiety subscales of the SCL-90, administered nationwide through the ICSRA platform, align with the prevalence of visits to paediatric psychiatrists and clinical child psychologists.^2^ Survey items measuring screen time were developed by ICSRA but are comparable to other studies examining screen time among adolescents.^5,6^ Items pertaining to stressors and trauma were adapted from the Negative Life Events Scale.^7^

Class cohorts in Icelandic schools are grouped by birth year, so as no question is asked in the surveys regarding birth year or age, the grade was taken as a proxy for age (13-, 14-, or 15-years old), where grade 8 would include children of age 13 or due to turn 13 within the year, grade 9 for 14-year-olds and grade 10 for 15-year-olds. There may be some minor inaccuracies with this method but overall, it is uncommon for children to skip a school year, begin school early or be held back a year in Iceland.

There have been changes in the questions in the Youth in Iceland survey across time. Wording and focus of questions have been updated to reflect the changing nature of the times. However, the majority of questions were directly comparable across years. Details of the questions, and any changes over the study period are detailed in this document in Table S3.

Of note, there were changes in the response options for the ‘What is your gender?’ question over the years. In 2016 and 2018 questionnaire only allowed for “girl” or “boy”, in 2020 and 2021 “other” was added as a third category and in 2022 and 2023 “I don’t know” and “Non-binary” added as fourth and fifth possible options (Supplementary Table S3). To account for these differences gender was coded as “female”, “male” or “non-binary” in analyses, but there is limited opportunity to evaluate changes for non-binary individuals given the lack of recording pre-pandemic. All gender models were run in all cases as the binary options in 2016 and 2018 are likely to have included individuals who may have preferred to select another option for gender that was not available in those years.

The Youth in Iceland study does not directly ask any questions regarding household income, ethnicity or immigration status, therefore household status (two-parent or other) and language spoken at home are taken as proxies for sociodemographic status. The question “What language(s) do you speak at home?” had three levels of response, “Icelandic only”, “Icelandic and another language” or “a language other than Icelandic”. This was re-coded to be a binary variable where 1 equalled “Icelandic only” and 0 was assigned to the other two responses, as a proxy for immigration status.

In 2016 and 2018 the screen time gaming-related questions were regarding ‘gaming offline’ versus ‘gaming online’, while in subsequent years the questions were rephrased to ‘gaming alone’ versus ‘gaming with friends’. Although it is evident that ‘gaming with friends’ can happen both online and offline and that ‘gaming online’ could occur with both friends and solo; in order to be able to make comparisons across years the question ‘gaming offline’ was equated to ‘gaming alone’, and ‘gaming online’ was equated to ‘gaming with friends’. As the change in questions occurred during 2020, it is reasonable to assume that the vast majority of ‘gaming with friends’ did indeed occur virtually.

## Statistical analysis

Composite survey scores were used to aggregate questions within a certain theme (*e.g.* screen time usage, parental support statistics, mental health symptoms). As a result of combining multiple Likert-scale questions, resulting scores were double-bound, often skewed, exclusively positive integer scales. Assessment with the fitdistr R package was used to assess distribution^8^. Due to their heavy skewness, composite survey scores followed a beta distribution. Therefore, linear transformation is necessary to analyse them with beta-distribution models.

Composite scores (*y*) were rescaled:

*y’ = (y – min)/(max – min)*

where min and max refer to the minimum and maximum possible values of the scores, followed by compression to avoid absolute zero and one values where N=sample size^9,10^:

*y’’ = [y’(N – 1)+1/2]/N*

A subset of transformed variables was run using generalised linear mixed effects models with a beta distribution family, alongside comparative models run using the brms package with Bayesian model building methodologies (MCMC), based on recommendations for beta models^11^. Results from these models yielded highly similar predictions to ML/REML methods in the glmmTMB model, as such it was decided to proceed with glmmTMB methods for subsequent analyses as they are computationally less demanding.

Bioecological models evaluated whether the addition of variables improves model fit relative to the simplest ‘base’ model testing only gender, age and survey year. Due to trauma and screen time questions not being asked in 2021, data for this year was not included in the bioecological models and the base models were re-run without the year 2021 for model fit comparison purposes. Five models (base, whole (all variables), individual, home, school/peer) were produced for each mental health measure (depressive symptom, anxiety symptom, and hostility scores) for all genders (including non-binary), and girls and boys separately. Starting model formulas were specified with all independent variables of interest and built using the direction ‘order’ applying the Likelihood-Ratio Test (LRT). Generated formulas were then subjected to a ‘backward’ stepwise reduction applying the Bayesian Information Criterion (BIC) to constrain model build.

### Model refinement

Some bioecological models on the dataset indexed to 2016 for girls preserved the interaction term between survey year and parental social support. Incorporating this interaction accounted for significant amounts of variance in the model due to collinearity with survey year and resulted in individual model estimates for survey year predicting contrary results to the observed data and other models, while simultaneously elevating estimates for the dependent variable to a non-viable level. Therefore, the model building process was repeated for 2016 anxiety and depression whole environment models on the dataset for girls without the interaction term, results of which are displayed in Table S11.

## Missing data

Multiple imputation was used to address missing data following existing protocol.^12^ Table S4 illustrates the extent of missing data for gender and each outcome variable. We compared participants with complete and incomplete data on demographic variables and our outcomes. All demographic, outcomes and factors related to the ecological framework were included in the imputation models. In the imputation models, chronological age, mental health outcomes (*i.e.,* depressive symptoms, anxiety, and hostility), screen use variables, and parental support were coded as continuous variables. Gender, language spoken at home, residency and the trauma exposure variables were coded as categorical variables. Multiple imputation was conducted with the aregImpute function from the Hmisc package in R^13^. This function uses bootstrapping to approximate the process of drawing predicted values from a full Bayesian predictive distribution. For practical reasons and deemed sufficient to obtain reasonable predictions,^12^ the set of first imputations was used in the initial analyses and for data representation in tables.

# SUPPLEMENTARY RESULTS

## Screen time model interactions

There were several interactions between screen time media types, gender and age categories which are elaborated on here with statistical models presented in Table S8. In the all gender models, hours spent gaming alone and with a friend increased during the pandemic (Figure 3), with usage remaining higher in 2023 compared to 2016 (β= -0·28, 95% CI -0·34 to -0·22 and β=-0·17, 95% CI -0·22 to -0·11, respectively). There was no overall effect of age in the boys’ model, but an interaction reveals a significant margin in time spent gaming with friends between 13- and 15-year-olds in 2020 compared to 2023 (β= 0·27, 95% CI 0·14 - 0·40), with 15-year-olds increasing time spent on this activity relative to 13- and 14-year-olds.

The pattern in hours spent watching videos or films was variable across the study period but was overall lower in 2023 than any previous years, and in the gender stratified models there were no significant changes over time for girls, but there was more time spent on this activity by boys in 2018 (β=0·26, 95% CI 0·17-0·34) compared to 2023.

In the all gender models, social media usage was higher in 2023 than 2016 (β=-0·11, 95% CI -0·17 to -0·05), but not significantly different to any other years. Social media usage for girls in 2023 was significantly higher than in 2018 (β=-0·18, 95% CI -0·27 to -0·09). There were two significant interactions between survey year and the age 15, indicating a greater gap between 13- and 15-year-old social media usage in 2016 (β=0.27, 95% CI 0·14 - 0·39) and 2018 (β=0.26, 95% CI 0·13 - 0·38) compared to the situation in 2023, driven by an increase in time spent on social media by 13-year-olds in later years.

# SUPPLEMENTARY TABLES

Table S1. Literature review paper summary. Overview of studies on self-reported mental health among adolescents during the COVID-19 pandemic in population-based or nationally representative cohorts with measurements occurring between 2022-2024.

| **Author** | **Study design** | **Sample size** | **Age range**  **(in years)** | **Country** | **Timing of data collection** | **Main findings** |
| --- | --- | --- | --- | --- | --- | --- |
| Ravens-Sieberer *et al.* 2023^14^+ | Longitudinal cohort study | 1673 | 7 to 17 | Germany | May–June 2020; December 2020–January 2021; September–October 2021; February 2022; September–October 2022. Pre-pandemic data from a different study was used for comparison (2017/18) | Anxiety levels decreased in September/October 2022 but were still higher than prior pandemic.  Children (7 – 10 years-old) experienced more mental health problems than adolescents (14 – 17 years-old) during later stages of the pandemic (September/October 2021 through September/October 2022).  Risk factors for poor mental health included greater parental pandemic burden, parental mental health problems, low parental education, restricted living space and migration background. Protective factors included high family and social support and strong personal resources such as problem-solving skills and optimism. |
| Kaman *et al.* 2023^15^+ | Longitudinal cohort study | 1602 | 7 to 17 | Germany | May–June 2020; December 2020–January 2021; September–October 2021; February 2022. Pre-pandemic data from a different study was used for comparison (2014 to 2017) | Higher levels of mental health problems, such as emotional problems, conduct problems and hyperactivity/inattention, in 2022 than pre-pandemic.  Risk factors for poor mental health included parental mental disorders, parental stress due to the pandemic, low parental education and having a restricted living space. Protective factors against poor mental health included a positive family atmosphere, strong personal resources and social support. |
| Shoshani and Kor, 2023^16^++ | Longitudinal study | 3718 | 12 to 16 | Israel | September 2019, May 2020, May 2021 and May 2022 | Depressive symptoms and anxiety increased over the course of the pandemic, especially among girls and older adolescents. Screen use and social media use increased too, which was also more pronounced among girls.  Social support and maintaining daily routines served as protective factors against poor mental health. |
| Zijlmans *et al.* 2023^17^+++ | Repeated cross-sectional | 222 – 1333 | 8 to 18 | Netherlands | Pre-pandemic; April - May 2020; November - December 2020; March - April 2021; November - December 2021; March – April 2022 | Anxiety, depressive symptoms and anger increased in the beginning of the pandemic but started to decrease in April 2022 (albeit still higher than pre-pandemic). Gender and age did not predict increases in mental health problems during the pandemic. |
| Barbieri *et al.* 2023^18^++++ | Repeated cross-sectional | 5159 - 6675 | 7 to 19 | Italy | June 2021 and March 2022 | Depressive symptoms and anxiety remained stable between 2021 and 2022. Girls experienced higher levels of depressive symptoms and anxiety than boys and adolescents were more likely to experience mental health problems than children.  Risk factors for mental health problems included single parenthood and parental mental health issues and |
| Barbieri *et al.* 2023^19^++++ | Repeated cross-sectional | 1760 -1885 | 11 to 19 | Italy | June 2021 and March 2022 | Symptoms of depression and anxiety remained stable across assessments, with higher levels noted among girls than boys.  Single parent household and parental mental health problems were risk factors for greater mental health problems. |
| Reiß *et al*. 2023^20^+ | Data from different studies | 1500 - 7300 | 7 to 17 | Germany | Pre-pandemic 2002 – 2018; May – June 2020; December 2020 – January 2021; September – October 2021; February 2022; September – October 2022 | Mental health problems, emotional problems, conduct problems and hyperactivity/inattention, increased in the beginning of the pandemic and have decreased in 2022 but not returned to pre-pandemic levels. |
| Kiviruusu *et al.* 2024^21^ | Repeated cross- sectional | 119681 - 158897 | 13 to 20 | Finland | Before March-May 2019; March-May 2021; March-May 2023 | Anxiety and depressive symptoms in 2023 have remained higher than prior to the pandemic. For 14-16-year-old girls, these mental health problems increased from 2021 to 2023. For boys, anxiety and depressive symptoms decreased between 2021 and 2023 but not to pre-pandemic levels. |
| Van Oers *et al.* 2023^22^+++ | Repeated cross-sectional | 818 - 1056 | 8 to 18 | Netherlands | Pre-pandemic; April - May 2020; November - December 2020; March - April 2021; November - December 2021; March – April 2022; November – December 2022; March – April 2023 | Depressive symptoms, anxiety and anger in late 2022 and spring 2023 was worse than before the pandemic. Previously reported trends in decreases in mental health issues in 2022 did not continue post-pandemic. |
| Kaman *et al.* 2024^23^+ | Longitudinal cohort study | 744 | 7 to 20 | Germany | May-June 2020; December 2020–January 2021; September–October 2021; February 2022; September-October 2022. | 64% of participants displayed low internalizing symptoms, while 74% showed low externalizing symptoms. There was also a resilient group that remained stable throughout the pandemic. However, 79% of youths reported moderate psychosomatic symptoms, highlighting a significant concern. The study also found that 10% of children experienced persistent internalizing mental health problems, and 18% faced increasing externalizing issues. Notably, improvement groups emerged, with 2 to 18% of initially impaired participants showing recovery, suggesting that some children's mental health needs were met over time. |
| Park *et al.* 2024^24^ | Longitudinal cohort study | 1399 | 9 to 15 | Canada | Pandemic: July–August 2020; March–April 2021; November 2021–January 2022  Post-pandemic: January–July 2023 | Children’s mental health symptoms increased during the pandemic, anxiety and depressive symptoms being particularly prevalent. However, mental health problems began to improve as the pandemic subsided. Girls were more likely to experience persistent mental health problems, especially anxiety and depressive symptoms, compared to boys. While some children demonstrated resilience and recovery, others continued to experience high levels of distress, particularly in terms of psychosomatic and emotional symptoms. |
| Zoellner *et al.* 2024^25^+ | Longitudinal cohort study | 2471 | 7 to 17 | Germany | May-June 2020; December 2020–January 2021; September–October 2021; February 2022; September-October 2022. | Boys and younger children were at higher risk of developing mental health problems. Low parental education and single parenthood, parental depressive symptoms and parental burden were associated with greater mental health problems. Higher personal resources, family cohesion and social support was protective against mental health problems. |
| Shoshani (2024)^26^++ | Longitudinal study | 5127 | 9 to 17 | Israel | Pre-pandemic: September 2019; Pandemic: May 2020, May 2021; May 2022 | Mental health symptoms peaked in 2022. Children and adolescent perceived a decrease in social support throughout the pandemic and they reported being less satisfied with their lives. Girls reported higher mental health problems than boys, and older adolescents showed greater increases in depressive symptoms and somatization than younger adolescents and children. |
| Bhandari and Gupta (2024)^27^ | Repeated cross sectional | 27378 | 2 to 17 | US | 2019, 2020, 2021 and 2022 | Notable fluctuations in mental health among adolescents, with a decline observed in 2020, partial recovery in 2021, and a subsequent worsening in 2022. Increase in both depression and anxiety, particularly among adolescents, though children also showed a significant rise in anxiety. |

*Notes*. + Findings from the same cohort . ++ Findings from the same cohort. +++ Findings from the same cohort. ++++ Findings from the same cohort.

Table S2. STROBE Statement—Checklist of items that should be included in reports of *cross-sectional studies.*

|  | Item No | Recommendation | Page No |
| --- | --- | --- | --- |
| Title and abstract | 1 | (*a*) Indicate the study’s design with a commonly used term in the title or the abstract | 1 |
|  |  | (*b*) Provide in the abstract an informative and balanced summary of what was done and what was found | 4 |
| Introduction | | |  |
| Background/rationale | 2 | Explain the scientific background and rationale for the investigation being reported | 2-3 |
| Objectives | 3 | State specific objectives, including any prespecified hypotheses | 5 |
| Methods | | |  |
| Study design | 4 | Present key elements of study design early in the paper | 5-6 |
| Setting | 5 | Describe the setting, locations, and relevant dates, including periods of recruitment, exposure, follow-up, and data collection | 6 |
| Participants | 6 | (*a*) Give the eligibility criteria, and the sources and methods of selection of participants | 5 |
| Variables | 7 | Clearly define all outcomes, exposures, predictors, potential confounders, and effect modifiers. Give diagnostic criteria, if applicable | 6-7 |
| Data sources/ measurement | 8* | For each variable of interest, give sources of data and details of methods of assessment (measurement). Describe comparability of assessment methods if there is more than one group | 6-7 |
| Bias | 9 | Describe any efforts to address potential sources of bias | 7 |
| Study size | 10 | Explain how the study size was arrived at | 6 |
| Quantitative variables | 11 | Explain how quantitative variables were handled in the analyses. If applicable, describe which groupings were chosen and why | 6-7 |
| Statistical methods | 12 | (*a*) Describe all statistical methods, including those used to control for confounding | 6-7 |
|  |  | (*b*) Describe any methods used to examine subgroups and interactions | 6-7 |
|  |  | (*c*) Explain how missing data were addressed | 7 |
|  |  | (*d*) If applicable, describe analytical methods taking account of sampling strategy | 6-7 |
|  |  | (*e*) Describe any sensitivity analyses | 6-7 |
| Results | | |  |
| Participants | 13* | (a) Report numbers of individuals at each stage of study—e.g. numbers potentially eligible, examined for eligibility, confirmed eligible, included in the study, completing follow-up, and analysed | 7 |
|  |  | (b) Give reasons for non-participation at each stage | 6 |
|  |  | (c) Consider use of a flow diagram | NA |
| Descriptive data | 14* | (a) Give characteristics of study participants (e.g. demographic, clinical, social) and information on exposures and potential confounders | 7 |
|  |  | (b) Indicate number of participants with missing data for each variable of interest | 7 |
| Outcome data | 15* | Report numbers of outcome events or summary measures | 7-10 |
| Main results | 16 | (*a*) Give unadjusted estimates and, if applicable, confounder-adjusted estimates and their precision (e.g., 95% confidence interval). Make clear which confounders were adjusted for and why they were included | 7-10 |
|  |  | (*b*) Report category boundaries when continuous variables were categorized | NA |
|  |  | (*c*) If relevant, consider translating estimates of relative risk into absolute risk for a meaningful time period | NA |
| Other analyses | 17 | Report other analyses done—e.g. analyses of subgroups and interactions, and sensitivity analyses | 7-10 |
| Discussion | | |  |
| Key results | 18 | Summarise key results with reference to study objectives | 10-11 |
| Limitations | 19 | Discuss limitations of the study, taking into account sources of potential bias or imprecision. Discuss both direction and magnitude of any potential bias | 11 |
| Interpretation | 20 | Give a cautious overall interpretation of results considering objectives, limitations, multiplicity of analyses, results from similar studies, and other relevant evidence | 10-11 |
| Generalisability | 21 | Discuss the generalisability (external validity) of the study results | 11-12 |
| Other information | | |  |
| Funding | 22 | Give the source of funding and the role of the funders for the present study and, if applicable, for the original study on which the present article is based | 13 |

Table S3. Details of survey question phrasing and response options of demographic, mental health, trauma exposure and screen use variables utilised in the study. Details on changes in question phrasing and response options over the study period are detailed, as well as corresponding standard questionnaire scales where appropriate.

| Variables in the study | | Question | Response options |
| --- | --- | --- | --- |
| Demographics | | | |
|  | Gender | What is your gender? | 1. Boy 2. Girl 3. Other (Option in 2020 and later) 4. Non-binary (Option in 2022 and later) 5. Do not know (Option in 2022 and later) |
|  | Age | Which grade are you in? | 1. 8 2. 9 3. 10 |
|  | Language spoken at home | Is Icelandic spoken at your home? | 1. Yes, only Icelandic 2. Yes, Icelandic and another language 3. No, only another language |
| Mental health problems | | | |
| - Depressive symptoms (SCL-90-R) | | | |
|  | How often did you experience the following distress or discomfort during the past week? | 1. You had little interest in doing things 2. You had little appetite 3. You felt lonely 4. You cried easily, or wanted to cry 5. You had difficulty falling asleep, or staying asleep 6. You felt depressed or sad 7. You were not excited to do anything 8. You felt sluggish or weak 9. You felt the future was hopeless 10. You thought about committing suicide | 1. Never 2. Rarely 3. Sometimes 4. Often |
| - Anxiety (SCL-90-R) | | | |
|  | How often did you experience the following distress or discomfort during the past week? | 1. Headache 2. Stomach pain 3. Nervousness 4. Sudden fear for no reason 5. You were excited/tense | 1. Never 2. Rarely 3. Sometimes 4. Often |
| - Hostility (SCL-90-R) | | | |
|  | How often did you experience the following distress or discomfort during the past week? | 1. You were easily annoyed or irritated 2. You had tantrums you couldn’t control 3. You wanted to break or smash things 4. You had an argument 5. You screamed or threw things | 1. Never 2. Rarely 3. Sometimes 4. Often |
| Stress and traumatic significant life events (NLES) | | | |
|  | Has any of the following happened to you? (Mark one or more options as appropriate) | 1. You had a serious accident 2. You had a serious illness 3. Your parents divorced or separated 4. You had a serious fight with your parents 5. You witnessed a serious fight between your parents 6. You witnessed physical abuse at home where an adult was involved 7. You experienced physical abuse at home where an adult was involved 8. Your friend died 9. You broke up with your boyfriend/girlfriend 10. You got an unusually bad grade 11. Father or mother lost their job 12. Parent or sibling died 13. You were sexually abused by an adult 14. You were sexually abused by a peer | 1. Yes, within the last 30 days 2. Yes, within the last 12 months 3. Yes, more than 12 months ago 4. No |
| Screen usage | | | |
|  | Screen time (all years) | How much time do you spend on the following on average per day?   1. Watching movies, shows or videos 2. Being on social media (*e.g.* Facebook, Snapchat, Instagram, TikTok...) | 1. Nearly no time 2. ½ - 1 hour 3. About 1 hour 4. About 2 hours 5. About 3 hours 6. About 4 hours 7. About 5 hours 8. 6 hours or more |
|  | Screen time (2016 and 2018) | How much time do you spend on the following on average per day?   1. Playing video games online 2. Playing video games offline 3. Using the internet for something other than social media or video games 4. Using computers for something other than being online or playing videogames (*e.g.* studying, writing text, working with images) | 1. Nearly no time 2. ½ - 1 hour 3. About 1 hour 4. About 2 hours 5. About 3 hours 6. About 4 hours 7. About 5 hours 8. 6 hours or more |
|  | Screen time (2020 and later) | How much time do you spend on the following on average per day?   1. Playing video games alone 2. Playing video games with others (*e.g.* with friends, with people online) 3. Using computers for something else (*e.g.* studying, reading news, writing text, working with images) | 1. Nearly no time 2. ½ - 1 hour 3. About 1 hour 4. About 2 hours 5. About 3 hours 6. About 4 hours 7. About 5 hours 8. 6 hours or more |
| Parental support | | | |
|  | How easy or difficult is it for you to: | 1. Get affection and warmth from your parents? 2. Discuss personal matters with your parents? 3. Get advice about your schoolwork from your parents? 4. Get advice about other things from your parents? 5. Get help with various tasks from your parents? | 1. Very difficult 2. Pretty difficult 3. Pretty easy 4. Very easy |

*Notes: SLC-90-R = Revised Symptom checklist 90, NLES = Negative Life Events Scale. Questions are presented in English in this document but were originally presented in Icelandic in all years, and available in English language in 2020 and subsequent years, and additionally in the Polish language in 2022 and subsequent years.

Table S4. The extent of missing data for demographic and mental health questions (depressive symptom, anxiety symptom and hostility scores) in the study across the study period (2016-2023). Values presented are the number of cases where the questions were not answered (n) and the proportion to the total sample size (%).

| Survey questions | **2016** | | **2018** | **2020** | **2021** | **2022** | **2023** |
| --- | --- | --- | --- | --- | --- | --- | --- |
| Age | 130 (1.22%) | | 122 (1.15%) | 13 (0.14%) | 10 (0.08%) | 207 (2.02%) | 105 (1.10%) |
| Gender | 107 (1.00%) | | 113 (1.07%) | 3 (0.03%) | 5 (0.04%) | 202 (1.97%) | 102 (1.07%) |
| Language spoken at home | 66 (0.62%) | | 72 (0.68%) | 8 (0.09%) | 5 (0.04%) | 260 (2.53%) | 158 (1.66%) |
| **Mental health symptom question categories** | |  |  |  |  |  |  |
| Depressive symptom scores | 131 (1.23%) | | 141 (1.33%) | 173 (1.89%) | 112 (0.95%) | 390 (3.80%) | 505 (5.29%) |
| Anxiety symptom scores | 119 (1.11%) | | 131 (1.24%) | 168 (1.83%) | 110 (0.93%) | 381 (3.71%) | 478 (5.01%) |
| Hostility scores | 153 (1.43%) | | 167 (1.58%) | 263 (2.87%) | 129 (1.09%) | 406 (3.96%) | 537 (5.63%) |

Table S5. Depressive symptoms, anxiety symptoms, and hostility scores for each gender-age group pairing for each year of the survey administration (2016-2018). Sample size (N) and mean (standard deviation) are presented.

|  | **2016** | **2018** | **2020** | **2021** | **2022** | **2023** |
| --- | --- | --- | --- | --- | --- | --- |
| **Girls 13-years-old** |  |  |  |  |  |  |
| Sample size | 1779 | 1830 | 1504 | 1895 | 1618 | 1579 |
| Depressive symptoms | 17.83 (7.43) | 18.18 (7.48) | 20.41 (7.54) | 20.97 (7.93) | 20.69 (7.75) | 19.53 (7.45) |
| Anxiety | 10.23 (3.75) | 10.36 (3.72) | 10.96 (3.76) | 11.29 (3.84) | 11.67 (3.90) | 11.20 (3.67) |
| Hostility | 7.97 (3.31) | 8.21 (3.47) | 9.38 (3.97) | 9.54 (3.99) | 9.29 (3.86) | 9.15 (3.83) |
| **Boys 13-years-old** |  |  |  |  |  |  |
| Sample size | 1747 | 1821 | 1547 | 2057 | 1800 | 1662 |
| Depressive symptoms | 15.03 (5.68) | 15.80 (6.21) | 16.61 (5.88) | 16.58 (5.99) | 16.91 (6.35) | 16.36 (6.15) |
| Anxiety | 8.32 (2.97) | 8.55 (3.00) | 8.78 (3.16) | 8.56 (3.04) | 9.01 (3.31) | 8.99 (3.27) |
| Hostility | 7.91 (3.19) | 8.39 (3.39) | 8.41 (3.38) | 8.46 (3.46) | 8.43 (3.49) | 8.54 (3.65) |
| **Girls 14-years-old** |  |  |  |  |  |  |
| Sample size | 1758 | 1783 | 1521 | 1905 | 1596 | 1491 |
| Depressive symptoms | 19.64 (8.14) | 19.33 (7.66) | 21.94 (8.01) | 22.24 (8.19) | 21.82 (7.91) | 21.17 (7.91) |
| Anxiety | 11.20 (4.05) | 10.99 (3.82) | 11.72 (3.91) | 11.77 (3.97) | 12.11 (4.08) | 11.94 (3.82) |
| Hostility | 8.47 (3.56) | 8.50 (3.34) | 9.55 (4.02) | 9.56 (3.93) | 9.44 (3.98) | 9.73 (4.02) |
| **Boys 14-years-old** |  |  |  |  |  |  |
| Sample size | 1791 | 1778 | 1606 | 2000 | 1715 | 1596 |
| Depressive symptoms | 15.02 (5.74) | 15.88 (6.03) | 16.52 (6.20) | 16.35 (6.20) | 16.71 (6.51) | 16.28 (5.92) |
| Anxiety | 8.43 (3.07) | 8.44 (2.87) | 8.47 (3.07) | 8.38 (3.10) | 8.66 (3.30) | 8.74 (3.16) |
| Hostility | 8.02 (3.16) | 8.33 (3.33) | 8.18 (3.37) | 8.05 (3.29) | 8.33 (3.58) | 8.48 (3.50) |
| **Girls 15-years-old** |  |  |  |  |  |  |
| Sample size | 1797 | 1696 | 1367 | 1791 | 1517 | 1463 |
| Depressive symptoms | 20.56 (8.46) | 20.07 (7.91) | 21.74 (7.88) | 22.00 (7.74) | 22.58 (7.92) | 21.08 (7.56) |
| Anxiety | 11.45 (4.04) | 11.19 (3.84) | 11.68 (3.85) | 11.71 (3.87) | 12.45 (3.90) | 11.95 (3.82) |
| Hostility | 8.61 (3.52) | 8.53 (3.45) | 9.46 (3.91) | 9.25 (3.59) | 9.34 (3.84) | 9.29 (3.81) |
| **Boys 15-years-old** |  |  |  |  |  |  |
| Sample size | 1812 | 1655 | 1455 | 1863 | 1592 | 1505 |
| Depressive symptoms | 16.09 (6.52) | 16.63 (6.12) | 16.83 (6.39) | 16.81 (6.57) | 16.28 (6.31) | 16.09 (6.52) |
| Anxiety | 8.47 (3.14) | 8.55 (3.15) | 8.54 (3.14) | 8.70 (3.25) | 8.58 (3.24) | 8.47 (3.14) |
| Hostility | 8.40 (3.35) | 8.51 (3.39) | 8.20 (3.30) | 7.93 (3.10) | 8.29 (3.50) | 8.40 (3.35) |

Table S6. Base model predictions for adolescent mental health outcomes (2023 Reference Year). Fixed effects of survey year (indexed to 2023), age, gender and interactions on mental health outcomes (depressive symptom scores, anxiety symptom scores and hostility scores), results are presented for all gender models, and gender-stratified models for girls and boys separately. Model estimates from beta mixed-effects models (β) are presented with confidence intervals (CI), alongside survey score equivalents to the cumulative survey scores, and significance value (*p*).

|  | **Depressive symptom scores (range 10-40)** | | | | | | | | | | | |
| --- | --- | --- | --- | --- | --- | --- | --- | --- | --- | --- | --- | --- |
|  | **All genders** | | | | **Girls** | | | | **Boys** | | | |
| **Predictors** | **β (CI)** | **Odds ratio** | **Survey score equivalent** | **p value** | **β (CI)** | **Odds ratio** | **Survey score equivalent** | **p value** | **β (CI)** | **Odds ratio** | **Survey score equivalent** | **p value** |
| 2016 | -0.380 (-0.44 – -0.33) | 0.684 | 18.003 | <0.0001***** | -0.390 (-0.47 – -0.31) | 0.677 | 17.504 | <0.0001***** | -0.360 (-0.44 – -0.28) | 0.698 | 14.749 | <0.0001***** |
| 2018 | -0.260 (-0.31 – -0.20) | 0.771 | 18.759 | <0.0001***** | -0.300 (-0.38 – -0.22) | 0.741 | 18.027 | <0.0001***** | -0.190 (-0.26 – -0.11) | 0.827 | 15.468 | <0.0001***** |
| 2020 | 0.160 (0.10 – 0.21) | 1.174 | 21.500 | <0.0001***** | 0.180 (0.09 – 0.26) | 1.197 | 21.115 | <0.0001***** | 0.150 (0.07 – 0.23) | 1.162 | 17.160 | 0.0002***** |
| 2021 | 0.190 (0.13 – 0.24) | 1.209 | 21.736 | <0.0001***** | 0.230 (0.15 – 0.31) | 1.259 | 21.508 | <0.0001***** | 0.130 (0.06 – 0.21) | 1.139 | 17.053 | 0.0005 |
| 2022 | 0.180 (0.13 – 0.24) | 1.197 | 21.687 | <0.0001***** | 0.180 (0.10 – 0.26) | 1.197 | 21.138 | <0.0001***** | 0.160 (0.09 – 0.24) | 1.174 | 17.221 | <0.0001***** |
| 14-years-old | 0.120 (0.06 – 0.17) | 1.127 | 21.238 | <0.0001***** | 0.240 (0.16 – 0.33) | 1.271 | 21.577 | <0.0001***** | 0.000 (-0.08 – 0.08) | 1.000 | 16.355 | 0.953 |
| 15-years-old | 0.070 (0.01 – 0.12) | 1.073 | 20.882 | 0.021 | 0.240 (0.15 – 0.32) | 1.271 | 21.526 | <0.0001***** | -0.070 (-0.15 – 0.01) | 0.932 | 16.014 | 0.073 |
| Boys | -0.680 (-0.70 – -0.66) | 0.507 | 16.361 | <0.0001***** | .. | .. | .. | .. | .. |  | .. | .. |
| Non-binary | 0.540 (0.47 – 0.61) | 1.716 | 24.297 | <0.0001***** | .. | .. | .. | .. | .. |  | .. | .. |
| 2016*14-years-old | 0.080 (0.00 – 0.16) | 1.083 | 20.967 | 0.044 | 0.100 (-0.02 – 0.21) | 1.105 | 20.549 | 0.098 | 0.060 (-0.05 – 0.16) | 1.062 | 0.310 | 0.310 |
| 2018*14-years-old | 0.060 (-0.02 – 0.14) | 1.062 | 20.841 | 0.120 | -0.010 (-0.12 – 0.10) | 0.990 | 19.843 | 0.877 | 0.120 (0.01 – 0.22) | 1.127 | 0.032 | 0.032 |
| 2020*14-years-old | -0.080 (-0.16 – -0.00) | 0.923 | 19.857 | 0.042 | -0.060 (-0.18 – 0.06) | 0.942 | 19.485 | 0.297 | -0.080 (-0.20 – 0.03) | 0.923 | 0.134 | 0.134 |
| 2021*14-years-old | -0.100 (-0.18 – -0.02) | 0.905 | 19.749 | 0.010 | -0.090 (-0.20 – 0.02) | 0.914 | 19.304 | 0.111 | -0.060 (-0.17 – 0.04) | 0.942 | 0.226 | 0.226 |
| 2022*14-years-old | -0.150 (-0.23 – -0.07) | 0.861 | 19.441 | 0.0002***** | -0.110 (-0.23 – 0.01) | 0.896 | 19.200 | 0.072 | -0.160 (-0.27 – -0.05) | 0.852 | 0.004 | 0.004 |
| 2016*15-years-old | 0.220 (0.14 – 0.30) | 1.246 | 21.951 | <0.0001***** | 0.260 (0.14 – 0.37) | 1.297 | 21.676 | <0.0001***** | 0.160 (0.05 – 0.27) | 1.174 | 0.004 | 0.004 |
| 2018*15-years-old | 0.110 (0.03 – 0.19) | 1.116 | 21.186 | 0.006 | 0.090 (-0.03 – 0.20) | 1.094 | 20.488 | 0.136 | 0.090 (-0.01 – 0.20) | 1.094 | 0.087 | 0.087 |
| 2020*15-years-old | -0.010 (-0.09 – 0.08) | 0.990 | 20.372 | 0.868 | -0.080 (-0.20 – 0.04) | 0.923 | 19.403 | 0.218 | 0.050 (-0.06 – 0.16) | 1.051 | 0.389 | 0.389 |
| 2021*15-years-old | 0.010 (-0.07 – 0.08) | 1.010 | 20.463 | 0.871 | -0.070 (-0.18 – 0.05) | 0.932 | 19.453 | 0.24 | 0.090 (-0.01 – 0.20) | 1.094 | 0.090 | 0.090 |
| 2022*15-years-old | -0.040 (-0.12 – 0.04) | 0.961 | 20.128 | 0.287 | 0.050 (-0.07 – 0.17) | 1.051 | 20.212 | 0.444 | -0.110 (-0.22 – -0.00) | 0.896 | 0.043 | 0.043 |
|  | **Anxiety symptom scores (range 5-20)** | | | | | | | | | | | |
| **Predictors** | **β (CI)** | **Odds ratio** | **Survey score equivalent** | **p value** | **β (CI)** | **Odds ratio** | **Survey score equivalent** | **p value** | **β (CI)** | **Odds ratio** | **Survey score equivalent** | **p value** |
| 2016 | -0.290 (-0.35 – -0.24) | 0.748 | 21.216 | <0.0001***** | -0.340 (-0.42 – -0.26) | 0.712 | 11.337 | <0.0001***** | -0.250 (-0.33 – -0.17) | 0.779 | 8.09 | <0.0001***** |
| 2018 | -0.200 (-0.26 – -0.15) | 0.819 | 21.854 | <0.0001***** | -0.290 (-0.37 – -0.20) | 0.748 | 10.136 | <0.0001***** | -0.120 (-0.19 – -0.04) | 0.887 | 8.43 | 0.003 |
| 2020 | -0.070 (-0.13 – -0.01) | 0.932 | 22.829 | 0.019 | -0.110 (-0.20 – -0.03) | 0.896 | 10.318 | 0.009 | -0.020 (-0.10 – 0.06) | 0.980 | 8.70 | 0.658 |
| 2021 | -0.080 (-0.14 – -0.03) | 0.923 | 22.737 | 0.003 | -0.050 (-0.13 – 0.03) | 0.951 | 10.926 | 0.202 | -0.130 (-0.20 – -0.05) | 0.878 | 8.40 | 0.001 |
| 2022 | 0.090 (0.04 – 0.15) | 1.094 | 24.047 | 0.001 | 0.130 (0.05 – 0.22) | 1.139 | 11.145 | 0.002 | -0.040 (-0.04 – 0.12) | 1.041 | 8.86 | 0.314 |
| 14-years-old | 0.040 (-0.01 – 0.10) | 1.041 | 23.676 | 0.134 | 0.180 (0.09 – 0.26) | 1.197 | 11.823 | <0.0001***** | -0.080 (-0.15 – 0.00) | 0.923 | 8.54 | 0.063 |
| 15-years-old | 0.010 (-0.04 – 0.07) | 1.010 | 23.449 | 0.648 | 0.230 (0.14 – 0.31) | 1.259 | 11.994 | <0.0001***** | -0.170 (-0.25 – -0.09) | 0.844 | 8.28 | <0.0001* |
| Boys | -0.920 (-0.94 – -0.90) | 0.399 | 17.268 | <0.0001***** | .. | .. | .. | .. | .. | .. | .. | .. |
| Non-binary | 0.230 (0.16 – 0.31) | 1.259 | 25.095 | <0.0001***** | .. | .. | .. | .. | .. | .. | .. | .. |
| 2016*14-years-old | 0.140 (0.06 – 0.22) | 1.150 | 24.390 | 0.001 | 0.110 (-0.01 – 0.23) | 1.116 | 11.747 | 0.061 | 0.150 (0.04 – 0.26) | 1.162 | 9.20 | 0.006 |
| 2018*14-years-old | 0.060 (-0.02 – 0.14) | 1.062 | 23.798 | 0.137 | 0.030 (-0.08 – 0.15) | 1.030 | 11.460 | 0.571 | 0.070 (-0.04 – 0.18) | 1.073 | 8.94 | 0.233 |
| 2020*14-years-old | 0.000 (-0.09 – 0.08) | 1.000 | 23.326 | 0.943 | 0.070 (-0.05 – 0.19) | 1.073 | 11.593 | 0.259 | -0.060 (-0.17 – 0.05) | 0.942 | 8.58 | 0.282 |
| 2021*14-years-old | -0.010 (-0.09 – 0.07 | 0.990 | 23.283 | 0.824 | 0.010 (-0.10 – 0.13) | 1.010 | 11.389 | 0.809 | 0.000 (-0.11 – 0.11) | 1.000 | 8.74 | 0.985 |
| 2022*14-years-old | -0.090 (-0.17 – -0.01) | 0.914 | 22.686 | 0.027 | -0.030 (-0.15 – 0.09) | 0.970 | 11.244 | 0.677 | -0.140 (-0.25 – -0.03) | 0.869 | 8.38 | 0.015 |
| 2016*15-years-old | 0.200 (0.12 – 0.28) | 1.221 | 24.856 | <0.0001***** | 0.180 (0.07 – 0.30) | 1.197 | 12.012 | 0.002 | 0.200 (0.09 – 0.31) | 1.221 | 9.35 | 0.0003***** |
| 2018*15-years-old | 0.070 (-0.01 – 0.15) | 1.073 | 23.897 | 0.072 | -0.010 (-0.13 – 0.11) | 0.990 | 11.302 | 0.871 | 0.130 (0.02 – 0.24) | 1.139 | 9.12 | 0.023 |
| 2020*15-years-old | 0.020 (-0.06 – 0.10) | 1.020 | 23.498 | 0.636 | -0.020 (-0.15 – 0.10) | 0.980 | 11.255 | 0.722 | 0.060 (-0.05 – 0.18) | 1.062 | 8.92 | 0.298 |
| 2021*15-years-old | 0.050 (-0.03 – 0.13) | 1.051 | 23.695 | 0.243 | -0.080 (-0.20 – 0.03) | 0.923 | 11.033 | 0.158 | 0.170 (0.06 – 0.28) | 1.185 | 9.25 | 0.002 |
| 2022*15-years-old | -0.030 (-0.11 – 0.05) | 0.970 | 23.144 | 0.502 | -0.020 (-0.14 – 0.10) | 0.980 | 11.259 | 0.728 | -0.030 (-0.14 – 0.09) | 0.970 | 8.68 | 0.654 |
|  | **Hostility scores (range 5-20)** | | | | | | | | | | | |
| **Predictors** | **β (CI)** | **Odds ratio** | **Survey score equivalent** | **p value** | **β (CI)** | **Odds ratio** | **Survey score equivalent** | **p value** | **β (CI)** | **Odds ratio** | **Survey score equivalent** | **p value** |
| 2016 | -0.260 (-0.31 – -0.20) | 0.771 | 8.722 | <0.0001***** | -0.340 (-0.42 – -0.26) | 0.712 | 8.424 | <0.0001***** | -1.150 (-0.23 – -0.07) | 0.861 | 8.120 | 0.0002***** |
| 2018 | -0.120 (-0.18 – -0.07) | 0.887 | 9.105 | <0.0001***** | -0.270 (-0.35 – -0.19) | 0.763 | 8.627 | <0.0001* | -0.060 (-0.02 – 0.14) | 1.062 | 8.666 | 0.168 |
| 2020 | 0.030 (-0.03 – 0.09) | 1.030 | 9.581 | 0.019 | 0.070 (-0.02 – 0.16) | 1.073 | 9.632 | 0.116 | 0.000 (-0.08 – 0.08) | 1.000 | 8.515 | 0.995 |
| 2021 | 0.070 (0.02 – 0.13) | 1.073 | 9.712 | 0.003 | 0.130 (0.05 – 0.21) | 1.139 | 9.835 | 0.002 | 0.000 (-0.07 – 0.08) | 1.000 | 8.525 | 0.92 |
| 2022 | 0.040 (-0.02 – 0.09) | 1.041 | 9.594 | 0.001 | 0.060 (-0.02 – 0.15) | 1.062 | 9.608 | 0.154 | 0.010 (-0.07 – 0.09) | 1.010 | 8.530 | 0.881 |
| 14-years-old | 0.080 (0.02 – 0.14) | 1.083 | 9.736 | 0.008 | 0.200 (0.12 – 0.29) | 1.221 | 10.071 | <0.0001***** | 0.030 (-0.11 – 0.05) | 0.970 | 8.431 | 0.452 |
| 15-years-old | -0.020 (-0.04 – 0.08) | 1.020 | 9.541 | 0.543 | 0.170 (0.08 – 0.25) | 1.185 | 9.944 | 0.0002***** | -0.100 (-0.18 – -0.02) | 0.905 | 8.252 | 0.017 |
| Boys | 0.320 (-0.34 – -0.30) | 0.726 | 8.554 | <0.0001***** | .. | .. | .. | .. | .. | .. | .. | .. |
| Non-binary | 0.390 (0.31 – 0.46) | 1.477 | 10.787 | <0.0001***** | .. | .. | .. | .. | .. | .. | .. | .. |
| 2016*14-years-old | -0.030 (-0.05 – 0.11) | 1.030 | 9.574 | 0.48 | -0.030 (-0.14 – 0.09) | 0.970 | 9.334 | 0.670 | 0.060 (-0.05 – 0.17) | 1.062 | 8.685 | 0.274 |
| 2018*14-years-old | -0.020 (-0.10 – 0.06) | 0.980 | 9.428 | 0.671 | -0.060 (-0.17 – 0.06) | 0.942 | 9.243 | 0.354 | -0.020 (-0.13 – 0.10) | 0.980 | 8.472 | 0.782 |
| 2020*14-years-old | -0.070 (-0.16 – 0.01) | 0.932 | 9.253 | 0.081 | -0.120 (-0.25 – -0.00) | 0.887 | 9.036 | 0.048 | -0.030 (-0.14 – 0.09) | 0.970 | 8.435 | 0.617 |
| 2021*14-years-old | -0.150 (-0.22 – -0.07) | 0.861 | 9.039 | 0.0003***** | -0.170 (-0.29 – -0.06) | 0.844 | 8.896 | 0.004 | -0.100 (-0.21 – 0.01) | 0.905 | 8.249 | 0.068 |
| 2022*14-years-old | 0.140 (-0.22 – -0.06) | 0.869 | 9.063 | 0.001 | -0.150 (-0.27 – -0.02) | 0.861 | 8.974 | 0.019 | -0.110 (-0.22 – 0.01) | 0.896 | 8.239 | 0.066 |
| 2016*15-years-old | 0.150 (0.07 – 0.23) | 1.162 | 9.974 | 0.0002***** | 0.090 (-0.02 – 0.21) | 1.094 | 9.705 | 0.122 | 0.180 (0.07 – 0.29) | 1.197 | 9.022 | 0.002 |
| 2018*15-years-old | 0.080 (-0.00 – 0.16) | 1.083 | 9.729 | 0.064 | 0.020 (-0.09 – 0.14) | 1.020 | 9.487 | 0.695 | 0.080 (-0.03 – 0.19) | 1.083 | 8.736 | 0.165 |
| 2020*15-years-old | -0.050 (-0.03 – 0.14) | 1.051 | 9.647 | 0.233 | -0.080 (-0.20 – 0.05) | 0.923 | 9.181 | 0.236 | 0.160 (0.05 – 0.28) | 1.174 | 8.976 | 0.006 |
| 2021*15-years-old | -0.060 (-0.14 – 0.02) | 0.942 | 9.286 | 0.118 | -0.180 (-0.30 – -0.07) | 0.835 | 8.865 | 0.002 | 0.050 (-0.07 – 0.16) | 1.051 | 8.637 | 0.421 |
| 2022*15-years-old | -0.080 (-0.16 – 0.00) | 0.923 | 9.232 | 0.052 | -0.070 (-0.19 – 0.05) | 0.932 | 9.200 | 0.264 | -0.080 (-0.20 – 0.03) | 0.923 | 8.297 | 0.155 |

*Note:* *: Significant after Bonferroni correction (<0.00043)

Table S7. Model predictions for proportions of adolescents with high mental health scores**.** Fixed effects of time, age and interactions on the proportion of individuals with high depression, anxiety and hostility scores (scores equal to or above those of the 5th centile determined separately for boys and girls for each age group in 2016). Model estimates presented from logistic mixed effects models (odd ratio, OR) with CI and statistical significance (*p*). Models are presented separately for girls and boys.

| **High-threshold depressive symptom scores** | **Girls** | | | **Boys** | | |
| --- | --- | --- | --- | --- | --- | --- |
|  | **OR** | **CI** | **p value** | **OR** | **CI** | **p value** |
| 2016 | 0.673 | 0.495 - 0.917 | 0.012 | 0.638 | 0.470 - 0.867 | 0.004 |
| 2018 | 0.717 | 0.530 - 0.969 | 0.031 | 1.055 | 0.806 - 1.381 | 0.695 |
| 2020 | 1.111 | 0.833 - 1.481 | 0.473 | 0.877 | 0.655 - 1.174 | 0.376 |
| 2021 | 1.441 | 1.108 - 1.874 | 0.006 | 0.994 | 0.763 - 1.296 | 0.966 |
| 2022 | 1.336 | 1.016 - 1.757 | 0.038 | 1.237 | 0.953 - 1.607 | 0.110 |
| 14-years-old | 0.773 | 0.565 - 1.057 | 0.107 | 0.865 | 0.647 - 1.156 | 0.326 |
| 15-years-old | 0.521 | 0.366 - 0.741 | 0.0003***** | 0.849 | 0.632 - 1.142 | 0.280 |
| 2016*14-years-old | 1.230 | 0.784 - 1.930 | 0.368 | 1.348 | 0.877 - 2.072 | 0.173 |
| 2018*14-years-old | 0.875 | 0.551 - 1.390 | 0.571 | 1.005 | 0.677 - 1.493 | 0.978 |
| 2020*14-years-old | 1.008 | 0.655 - 1.551 | 0.971 | 1.409 | 0.935 - 2.122 | 0.101 |
| 2021*14-years-old | 0.839 | 0.563 - 1.251 | 0.390 | 1.221 | 0.833 - 1.789 | 0.307 |
| 2022*14-years-old | 0.760 | 0.498 - 1.162 | 0.205 | 1.166 | 0.797 - 1.705 | 0.429 |
| 2016*15-years-old | 1.845 | 1.145 - 2.973 | 0.012 | 1.362 | 0.883 - 2.102 | 0.163 |
| 2018*15-years-old | 1.299 | 0.793 - 2.129 | 0.299 | 1.079 | 0.723 - 1.612 | 0.709 |
| 2020*15-years-old | 0.956 | 0.582 - 1.568 | 0.858 | 1.254 | 0.820 - 1.916 | 0.297 |
| 2021*15-years-old | 0.723 | 0.456 - 1.146 | 0.168 | 1.323 | 0.898 - 1.948 | 0.157 |
| 2022*15-years-old | 0.964 | 0.605 - 1.534 | 0.876 | 1.107 | 0.749 - 1.635 | 0.610 |
| **High-threshold anxiety symptom scores** | **Girls** | | | **Boys** | | |
|  | **OR** | **CI** | **p value** | **OR** | **CI** | **p value** |
| 2016 | 0.800 | 0.589 - 1.087 | 0.154 | 0.562 | 0.421 - 0.748 | 0.006 |
| 2018 | 0.764 | 0.561 - 1.039 | 0.086 | 0.640 | 0.486 - 0.844 | 0.0003* |
| 2020 | 0.913 | 0.669 - 1.245 | 0.566 | 1.272 | 0.990 - 1.633 | 0.341 |
| 2021 | 1.088 | 0.820 - 1.445 | 0.558 | 1.307 | 1.031 - 1.657 | 0.002 |
| 2022 | 1.476 | 1.119 - 1.948 | 0.006 | 1.073 | 0.833 - 1.383 | 0.653 |
| 14-years-old | 0.371 | 0.247 - 0.557 | <0.0001***** | 1.075 | 0.831 - 1.391 | 0.417 |
| 15-years-old | 0.450 | 0.307 - 0.659 | <0.0001***** | 0.829 | 0.630 - 1.090 | 0.0002* |
| 2016*14-years-old | 1.570 | 0.913 - 2.700 | 0.103 | 0.954 | 0.638 - 1.426 | 0.525 |
| 2018*14-years-old | 1.087 | 0.611 - 1.934 | 0.777 | 0.628 | 0.416 - 0.948 | 0.757 |
| 2020*14-years-old | 1.296 | 0.740 - 2.270 | 0.364 | 0.704 | 0.490 - 1.010 | 0.473 |
| 2021*14-years-old | 1.308 | 0.782 - 2.190 | 0.307 | 0.607 | 0.430 - 0.858 | 0.185 |
| 2022*14-years-old | 1.409 | 0.853 - 2.327 | 0.181 | 0.747 | 0.518 - 1.077 | 0.778 |
| 2016*15-years-old | 1.351 | 0.803 - 2.275 | 0.257 | 1.175 | 0.776 - 1.779 | 0.057 |
| 2018*15-years-old | 0.712 | 0.393 - 1.288 | 0.262 | 0.906 | 0.596 - 1.377 | 0.286 |
| 2020*15-years-old | 0.772 | 0.428 - 1.392 | 0.389 | 0.846 | 0.577 - 1.240 | 0.773 |
| 2021*15-years-old | 0.758 | 0.447 - 1.287 | 0.305 | 0.519 | 0.354 - 0.760 | 0.114 |
| 2022*15-years-old | 0.870 | 0.525 - 1.441 | 0.589 | 0.946 | 0.646 - 1.386 | 0.772 |
| **High-threshold hostility scores** | **Girls** | | | **Boys** | | |
|  | **OR** | **CI** | **p value** | **OR** | **CI** | **p value** |
| 2016 | 0.562 | 0.421 - 0.748 | <0.0001***** | 0.561 | 0.407 - 0.775 | <0.001* |
| 2018 | 0.640 | 0.486 - 0.844 | 0.002 | 0.726 | 0.539 - 0.978 | 0.035 |
| 2020 | 1.272 | 0.990 - 1.633 | 0.060 | 0.719 | 0.526 - 0.983 | 0.039 |
| 2021 | 1.307 | 1.031 - 1.657 | 0.027 | 0.799 | 0.603 - 1.060 | 0.119 |
| 2022 | 1.073 | 0.833 - 1.383 | 0.585 | 0.895 | 0.674 - 1.189 | 0.446 |
| 14-years-old | 1.075 | 0.831 - 1.391 | 0.580 | 0.722 | 0.531 - 0.981 | 0.038 |
| 15-years-old | 0.829 | 0.630 - 1.090 | 0.179 | 0.765 | 0.562 - 1.041 | 0.088 |
| 2016*14-years-old | 0.954 | 0.638 - 1.426 | 0.819 | 1.451 | 0.913 - 2.305 | 0.116 |
| 2018*14-years-old | 0.628 | 0.416 - 0.948 | 0.027 | 1.416 | 0.916 - 2.188 | 0.117 |
| 2020*14-years-old | 0.704 | 0.490 - 1.010 | 0.056 | 1.171 | 0.737 - 1.861 | 0.505 |
| 2021*14-years-old | 0.607 | 0.430 - 0.858 | 0.005 | 1.025 | 0.667 - 1.575 | 0.910 |
| 2022*14-years-old | 0.747 | 0.518 - 1.077 | 0.118 | 1.375 | 0.905 - 2.091 | 0.136 |
| 2016*15-years-old | 1.175 | 0.776 - 1.779 | 0.446 | 1.345 | 0.845 - 2.141 | 0.212 |
| 2018*15-years-old | 0.906 | 0.596 - 1.377 | 0.644 | 1.252 | 0.804 - 1.951 | 0.320 |
| 2020*15-years-old | 0.846 | 0.577 - 1.240 | 0.391 | 1.422 | 0.901 - 2.244 | 0.130 |
| 2021*15-years-old | 0.519 | 0.354 - 0.760 | 0.001 | 1.081 | 0.704 - 1.659 | 0.723 |
| 2022*15-years-old | 0.946 | 0.646 - 1.386 | 0.777 | 0.744 | 0.471 - 1.177 | 0.207 |

*Note*: *: Significant after Bonferroni correction (<0.00043).

Table S8. Model predictions for screen time across the study period (2023 Reference Year). Separate models were run separately for each media type (watching videos, playing video games with friends, playing video games alone, using social media, and other screen usage). Model estimates from beta mixed-effects models (β) are presented with CI, alongside odds ratios, survey score equivalents to the number of hours spent daily on each media type (full range was from 0-6+ hours), and significance value (*p*). All genders, and gender stratified models for girls and boys are presented separately.

| **Video watching** | | | | | | | | | | | | |
| --- | --- | --- | --- | --- | --- | --- | --- | --- | --- | --- | --- | --- |
|  | **All genders** | | | | **Girls** | | | | **Boys** | | | |
| **Predictors** | **β (CI)** | **Odds ratio** | **Survey score equivalent** | **p value** | **β (CI)** | **Odds ratio** | **Survey score equivalent** | **p value** | **β (CI)** | **Odds ratio** | **Survey score equivalent** | **p value** |
| 2016 | 0.061 (0.00 – 0.12) | 1.063 | 2.430 | 0.052 | -0.016 (-0.10 – 0.07) | 0.984 | 2.428 | 0.724 | 0.129 (0.04 – 0.22) | 1.138 | 2.487 | 0.004 |
| 2018 | 0.189 (0.13 – 0.25) | 1.208 | 2.519 | <0.0001* | 0.111 (0.02 – 0.20) | 1.117 | 2.613 | 0.012 | 0.258 (0.17 – 0.34) | 1.295 | 2.677 | <0.0001* |
| 2020 | 0.088 (0.02 – 0.15) | 1.092 | 2.707 | 0.007 | 0.015 (-0.07 – 0.11) | 1.015 | 2.473 | 0.740 | 0.153 (0.06 – 0.24) | 1.165 | 2.522 | 0.001 |
| 2022 | 0.143 (0.08 – 0.20) | 1.154 | 2.558 | <0.0001* | 0.125 (0.04 – 0.21) | 1.133 | 2.633 | 0.006 | 0.149 (0.06 – 0.23) | 1.160 | 2.515 | 0.001 |
| 14 years old | 0.091 (0.03 – 0.15) | 1.095 | 2.640 | 0.004 | 0.090 (0.00 – 0.18) | 1.095 | 2.583 | 0.048 | 0.100 (0.01 – 0.19) | 1.105 | 2.444 | 0.027 |
| 15 years old | 0.051 (-0.01 – 0.11) | 1.052 | 2.563 | 0.113 | 0.062 (-0.03 – 0.15) | 1.063 | 2.540 | 0.182 | 0.040 (-0.05 – 0.13) | 1.041 | 2.359 | 0.380 |
| Boy | -0.067 (-0.09 - -0.04) | 0.935 | 2.504 | <0.0001* | .. | .. | .. | .. | .. | .. | .. | .. |
| Non-binary | 0.227 (0.14 – 0.32) | 1.254 | 2.334 | <0.0001* | .. | .. | .. | .. | .. | .. | .. | .. |
| 2016*14 years old | 0.095 (0.01 – 0.18) | 1.099 | 2.764 | 0.032 | 0.091 (-0.03 – 0.21) | 1.095 | 2.583 | 0.146 | 0.091 (-0.03 – 0.21) | 1.095 | 2.431 | 0.148 |
| 2018*14 years old | 0.003 (-0.09 – 0.08) | 0.997 | 2.568 | 0.939 | 0.095 (-0.03 – 0.22) | 1.100 | 2.589 | 0.128 | -0.095 (-0.22 – 0.03) | 0.909 | 2.168 | 0.129 |
| 2020*14 years old | 0.013 (-0.08 – 0.10) | 1.013 | 2.426 | 0.775 | 0.085 (-0.04 – 0.21) | 1.088 | 2.574 | 0.193 | -0.57 (-0.18 – 0.07) | 0.945 | 2.222 | 0.382 |
| 2022*14 years old | -0.085 (-0.17 – 0.00) | 0.919 | 2.449 | 0.055 | -0.048 (-0.17 – 0.08) | 0.953 | 2.381 | 0.451 | -0.110 (-0.23 – 0.01) | 0.896 | 2.147 | 0.080 |
| 2016*14 years old | 0.115 (0.03 – 0.20) | 1.122 | 2.309 | 0.009 | 0.105 (-0.02 – 0.23) | 1.111 | 2.605 | 0.092 | 0.126 (0.00 – 0.25) | 1.134 | 2.482 | 0.046 |
| 2018*15 years old | 0.050 (-0.04 – 0.14) | 1.051 | 2.599 | 0.260 | 0.107 (-0.02 – 0.23) | 1.112 | 2.607 | 0.091 | 0.003 (-0.12 – 0.13) | 1.003 | 2.305 | 0.965 |
| 2020*15 years old | 0.177 (0.09 – 0.27) | 1.194 | 2.503 | 0.0001* | 0.193 (0.06 – 0.32) | 1.213 | 2.734 | 0.004 | 0.171 (0.04 – 0.30) | 1.187 | 2.548 | 0.010 |
| 2022*15 years old | 0.036 (-0.05 – 0.12) | 1.036 | 2.691 | 0.426 | 0.080 (-0.05 – 0.21) | 1.083 | 2.567 | 0.220 | 0.015 (-0.11 – 0.14) | 1.015 | 2.322 | 0.819 |
| **Gaming with friends** | | | | | | | | | | | | |
|  | **All genders** | | | | **Girls** | | | | **Boys** | | | |
| **Predictors** | **β (CI)** | **Odds ratio** | **Survey score equivalent** | **p value** | **β (CI)** | **Odds ratio** | **Survey score equivalent** | **p value** | **β (CI)** | **Odds ratio** | **Survey score equivalent** | **p value** |
| 2016 | -0.169 (-0.23 – -0.11) | 0.844 | 0.873 | <0.0001* | -0.168 (-0.24 – -0.09) | 0.845 | -13.552 | <0.0001* | -0.179 (-0.27 – -0.09) | 0.836 | -11.412 | <0.0001* |
| 2018 | -0.053 (-0.11 – 0.01) | 0.948 | 0.963 | 0.073 | -0.189 (-0.26 – -0.11) | 0.828 | -15.106 | <0.0001* | 0.083 (0.00 – 0.17) | 1.087 | 5.537 | 0.062 |
| 2020 | 0.106 (0.04 – 0.17) | 1.111 | 1.098 | 0.001 | 0.074 (-0.01 – 0.15) | 1.077 | 6.533 | 0.070 | 0.120 (0.03 – 0.21) | 1.128 | 8.020 | 0.010 |
| 2022 | 0.136 (0.08 – 0.20) | 1.146 | 1.126 | <0.0001* | 0.042 (-0.04 – 0.12) | 1.043 | 3.620 | 0.301 | 0.227 (0.14 – 0.32) | 1.255 | 15.380 | <0.0001* |
| 14 years old | -0.002 (-0.06 – 0.06) | 0.998 | 1.005 | 0.951 | -0.068 (-0.15 – 0.01) | 0.935 | -5.648 | 0.098 | 0.071 (-0.02 – 0.16) | 1.073 | 4.674 | 0.125 |
| 15 years old | -0.097 (-0.16 – -0.04) | 0.908 | 0.928 | 0.002 | -0.154 (-0.23 – -0.07) | 0.857 | -12.482 | 0.0002* | -0.046 (-0.14 – 0.04) | 0.955 | -3.030 | 0.317 |
| Boy | 1.021 (1.00 – 1.04) | 2.776 | 2.153 | <0.0001* | .. | 1.000 | .. | .. | .. | 1.000 | .. | .. |
| Non-binary | 0.561 (0.47 – 0.65) | 1.752 | 1.566 | <0.0001* | .. | 1.000 | .. | .. | .. | 1.000 | .. | .. |
| 2016*14 years old | 0.068 (-0.01 – 0.15) | 1.071 | 1.065 | 0.105 | 0.014 (-0.09 – 0.12) | 1.014 | 1.226 | 0.797 | 0.124 (0.00 – 0.25) | 1.132 | 8.257 | 0.051 |
| 2018*14 years old | 0.034 (-0.05 – 0.12) | 1.034 | 1.035 | 0.426 | 0.081 (-0.03 – 0.19) | 1.084 | 7.117 | 0.143 | -0.020 (-0.14 – 0.10) | 0.980 | -1.317 | 0.753 |
| 2020*14 years old | -0.028 (-0.11 – 0.06) | 0.973 | 0.984 | 0.531 | -0.085 (-0.20 – 0.03) | 0.918 | -7.075 | 0.142 | 0.026 (-0.10 – 0.16) | 1.026 | 1.696 | 0.696 |
| 2022*14 years old | -0.031 (-0.12 – 0.05) | 0.969 | 0.981 | 0.462 | -0.023 (-0.14 – 0.09) | 0.977 | -1.986 | 0.682 | -0.026 (-0.15 – 0.10) | 0.974 | -1.711 | 0.685 |
| 2016*14 years old | 0.061 (-0.02 – 0.14) | 1.063 | 1.059 | 0.150 | 0.031 (-0.08 – 0.14) | 1.031 | 2.647 | 0.580 | 0.102 (-0.02 – 0.23) | 1.108 | 6.808 | 0.107 |
| 2018*15 years old | 0.043 (-0.04 – 0.13) | 1.043 | 1.043 | 0.317 | 0.091 (-0.02 – 0.20) | 1.095 | 8.045 | 0.101 | 0.003 (-0.12 – 0.13) | 1.003 | 0.175 | 0.966 |
| 2020*15 years old | 0.086 (0.00 – 0.17) | 1.090 | 1.081 | 0.054 | -0.066 (-0.18 – 0.05) | 0.936 | -5.523 | 0.261 | 0.272 (0.14 – 0.40) | 1.313 | 18.510 | <0.0001* |
| 2022*15 years old | -0.061 (-0.15 – 0.02) | 0.941 | 0.956 | 0.156 | -0.070 (-0.18 – 0.04) | 0.933 | -5.825 | 0.224 | -0.027 (-0.15 – 0.10) | 0.973 | -1.769 | 0.679 |
| **Gaming alone** | | | | | | | | | | | | |
|  | **All genders** | | | | **Girls** | | | | **Boys** | | | |
| **Predictors** | **β (CI)** | **Odds ratio** | **Survey score equivalent** | **p value** | **β (CI)** | **Odds ratio** | **Survey score equivalent** | **p value** | **β (CI)** | **Odds ratio** | **Survey score equivalent** | **p value** |
| 2016 | -0.282 (-0.34 – -0.22) | 0.754 | 0.892 | <0.0001* | -0.174 (-0.25 – -0.10) | 0.840 | 0.710 | <0.0001* | -0.407 (-0.49 – -0.32) | 0.666 | 1.578 | <0.0001* |
| 2018 | -0.356 (-0.41 – -0.30) | 0.701 | 0.838 | <0.0001* | -0.201 (-0.28 – -0.12) | 0.818 | 0.694 | <0.0001* | -0.523 (-0.61 – -0.44) | 0.593 | 1.447 | <0.0001* |
| 2020 | -0.002 (-0.06 – 0.06) | 0.998 | 1.127 | 0.955 | 0.028 (-0.05 – 0.11) | 1.028 | 0.847 | 0.496 | -0.056 (-0.15 – 0.03) | 0.946 | 2.019 | 0.228 |
| 2022 | 0.094 (0.04 – 0.15) | 1.098 | 1.216 | 0.002 | 0.028 (-0.05 – 0.11) | 1.029 | 0.847 | 0.480 | 0.137 (0.05 – 0.23) | 1.147 | 2.285 | 0.002 |
| 14 years old | -0.048 (-0.11 – 0.01) | 0.953 | 1.085 | 0.115 | -0.068 (-0.15 – 0.01) | 0.934 | 0.779 | 0.091 | -0.046 (-0.14 – 0.04) | 0.955 | 2.032 | 0.314 |
| 15 years old | -0.120 (-0.18 – -0.06) | 0.887 | 1.022 | <0.0001* | -0.146 (-0.23 – -0.07) | 0.864 | 0.728 | 0.0003* | -0.110 (-0.20 – -0.02) | 0.896 | 1.947 | 0.017 |
| Boy | 0.712 (0.69 – 0.73) | 2.038 | 1.924 | <0.0001* | .. | .. | .. | .. | .. | .. | .. | .. |
| Non-binary | 0.725 (0.64 – 0.81) | 2.065 | 1.941 | <0.0001* | .. | .. | .. | .. | .. | .. | .. | .. |
| 2016*14 years old | 0.010 (-0.07 – 0.09) | 1.010 | 1.137 | 0.807 | 0.014 (-0.09 – 0.12) | 1.014 | 0.837 | 0.798 | 0.027 (-0.09 – 0.15) | 1.028 | 2.132 | 0.658 |
| 2018*14 years old | 0.034 (-0.05 – 0.11) | 1.034 | 1.159 | 0.414 | 0.072 (-0.04 – 0.18) | 1.075 | 0.880 | 0.186 | 0.019 (-0.10 – 0.14) | 1.019 | 2.120 | 0.762 |
| 2020*14 years old | 0.001 (-0.08 – 0.09) | 1.001 | 1.129 | 0.986 | -0.053 (-0.17 – 0.06) | 0.948 | 0.789 | 0.352 | 0.088 (-0.04 – 0.21) | 1.092 | 2.215 | 0.178 |
| 2022*14 years old | 0.009 (-0.07 – 0.09) | 1.009 | 1.136 | 0.831 | 0.050 (-0.06 – 0.16) | 1.051 | 0.863 | 0.383 | 0.011 (-0.11 – 0.14) | 1.011 | 2.110 | 0.860 |
| 2016*14 years old | 0.002 (-0.08 – 0.08) | 1.002 | 1.130 | 0.969 | 0.028 (-0.08 – 0.14) | 1.028 | 0.847 | 0.614 | -0.006 (-0.13 – 0.12) | 0.994 | 2.087 | 0.928 |
| 2018*15 years old | 0.023 (-0.06 – 0.10) | 1.023 | 1.149 | 0.588 | 0.083 (-0.02 – 0.19) | 1.087 | 0.888 | 0.132 | -0.020 (-0.14 – 0.10) | 0.980 | 2.067 | 0.747 |
| 2020*15 years old | 0.050 (-0.04 – 0.14) | 1.051 | 1.175 | 0.253 | -0.011 (-0.12 – 0.10) | 0.990 | 0.819 | 0.857 | 0.148 (0.02 – 0.28) | 1.159 | 2.300 | 0.026 |
| 2022*15 years old | -0.020 (-0.10 – 0.06) | 0.980 | 1.110 | 0.642 | 0.017 (-0.10 – 0.13) | 1.017 | 0.839 | 0.771 | -0.021 (-0.15 – 0.11) | 0.979 | 2.066 | 0.745 |
| **Social media** | | | | | | | | | | | | |
|  | **All genders** | | | | **Girls** | | | | **Boys** | | | |
| **Predictors** | **β (CI)** | **Odds ratio** | **Survey score equivalent** | **p value** | **β (CI)** | **Odds ratio** | **Survey score equivalent** | **p value** | **β (CI)** | **Odds ratio** | **Survey score equivalent** | **p value** |
| 2016 | -0.112 (-0.17 – -0.05) | 0.894 | 2.722 | 0.00042* | -0.142 (-0.23 – -0.05) | 0.867 | 2.686 | 0.002 | -0.077 (-0.16 – 0.01) | 0.926 | 1.922 | 0.082 |
| 2018 | -0.101 (-0.16 – -0.04) | 0.904 | 2.739 | 0.001 | -0.178 (-0.27 – -0.09) | 0.837 | 2.633 | <0.0001* | -0.022 (-0.11 – 0.06) | 0.978 | 1.995 | 0.615 |
| 2020 | -0.106 (-0.17 – -0.04) | 0.900 | 2.731 | 0.001 | -0.128 (-0.22 – -0.03) | 0.880 | 2.707 | 0.007 | -0.089 (-0.18 – 0.00) | 0.915 | 1.907 | 0.051 |
| 2022 | 0.057 (0.00 – 0.12) | 1.059 | 2.975 | 0.070 | 0.067 (-0.03 – 0.16) | 1.069 | 2.998 | 0.156 | 0.055 (-0.03 – 0.14) | 1.057 | 2.099 | 0.209 |
| 14 years old | 0.218 (0.15 – 0.28) | 1.243 | 3.215 | <0.0001* | 0.303 (0.21 – 0.40) | 1.355 | 3.352 | <0.0001* | 0.166 (0.08 – 0.26) | 1.181 | 2.253 | 0.0002* |
| 15 years old | 0.261 (0.20 – 0.33) | 1.298 | 3.280 | <0.0001* | 0.259 (0.17 – 0.35) | 1.295 | 3.285 | <0.0001* | 0.268 (0.18 – 0.36) | 1.308 | 2.398 | <0.0001* |
| Boy | -0.595 (-0.62 – -0.57) | 0.551 | 2.032 | <0.0001* | .. | .. | .. | .. | .. | .. | .. | .. |
| Non-binary | -0.232 (-0.32 – -0.14) | 0.793 | 2.544 | <0.0001* | .. | .. | .. | .. | .. | .. | .. | .. |
| 2016*14 years old | 0.137 (0.05 – 0.22) | 1.146 | 3.094 | 0.002 | 0.143 (0.02 – 0.27) | 1.154 | 3.112 | 0.027 | 0.105 (-0.02 – 0.23) | 1.111 | 2.168 | 0.092 |
| 2018*14 years old | 0.055 (-0.03 – 0.14) | 1.056 | 2.971 | 0.219 | 0.045 (-0.08 – 0.17) | 1.046 | 2.965 | 0.487 | 0.039 (-0.08 – 0.16) | 1.040 | 2.077 | 0.528 |
| 2020*14 years old | -0.032 (-0.12 – 0.06) | 0.969 | 2.842 | 0.492 | -0.040 (-0.17 – 0.09) | 0.961 | 2.838 | 0.554 | -0.042 (-0.17 – 0.08) | 0.959 | 1.968 | 0.515 |
| 2022*14 years old | -0.131 (-0.22 – -0.04) | 0.878 | 2.694 | 0.004 | -0.214 (-0.34 – -0.08) | 0.807 | 2.580 | 0.001 | -0.059 (-0.18 – 0.06) | 0.943 | 1.946 | 0.351 |
| 2016*14 years old | 0.210 (0.12 – 0.30) | 1.233 | 3.203 | <0.0001* | 0.267 (0.14 – 0.39) | 1.306 | 3.298 | <0.0001* | 0.159 (0.03 – 0.28) | 1.172 | 2.243 | 0.012 |
| 2018*15 years old | 0.177 (0.09 – 0.27) | 1.193 | 3.154 | <0.0001* | 0.256 (0.13 – 0.38) | 1.291 | 3.281 | <0.0001* | 0.105 (-0.02 – 0.23) | 1.110 | 2.167 | 0.101 |
| 2020*15 years old | 0.087 (-0.01 – 0.18) | 1.090 | 3.019 | 0.066 | 0.104 (-0.03 – 0.24) | 1.109 | 3.054 | 0.129 | 0.078 (-0.05 – 0.21) | 1.081 | 2.130 | 0.236 |
| 2022*15 years old | -0.126 (-0.22 – -0.04) | 0.881 | 2.701 | 0.006 | -0.070 (-0.20 – 0.06) | 0.932 | 2.793 | 0.295 | -0.190 (-0.32 – -0.06) | 0.827 | 1.777 | 0.003 |
| **Other screen usage** | | | | | | | | | | | | |
|  | **All genders** | | | | **Girls** | | | | **Boys** | | | |
| **Predictors** | **β (CI)** | **Odds ratio** | **Survey score equivalent** | **p value** | **β (CI)** | **Odds ratio** | **Survey score equivalent** | **p value** | **β (CI)** | **Odds ratio** | **Survey score equivalent** | **p value** |
| 2016 | -0.072 (-0.13 – -0.02) | 0.931 | 1.091 | 0.011 | -0.162 (-0.24 – -0.08) | 0.850 | 0.976 | <0.0001* | 0.010 (-0.07 – 0.09) | 1.010 | 1.139 | 0.804 |
| 2018 | -0.034 (-0.09 – 0.02) | 0.966 | 1.125 | 0.220 | -0.091 (-0.17 – -0.01) | 0.913 | 1.036 | 0.022 | 0.015 (-0.06 – 0.09) | 1.015 | 1.144 | 0.701 |
| 2020 | -0.080 (-0.14 – -0.02) | 0.924 | 1.085 | 0.006 | -0.075 (-0.16 – 0.01) | 0.928 | 1.049 | 0.070 | -0.086 (-0.17 – 0.00) | 0.918 | 1.053 | 0.038 |
| 2022 | 0.007 (-0.05 – 0.06) | 1.007 | 1.163 | 0.817 | -0.017 (-0.10 – 0.06) | 0.984 | 1.101 | 0.685 | 0.015 (-0.06 – 0.09) | 1.015 | 1.144 | 0.704 |
| 14 years old | 0.049 (-0.01 – 0.11) | 1.050 | 1.203 | 0.089 | 0.039 (-0.04 – 0.12) | 1.039 | 1.152 | 0.352 | 0.036 (-0.04 – 0.12) | 1.036 | 1.163 | 0.381 |
| 15 years old | 0.228 (0.17 – 0.29) | 1.256 | 1.384 | <0.0001* | 0.247 (0.16 – 0.33) | 1.280 | 1.358 | <0.0001* | 0.209 (0.13 – 0.29) | 1.233 | 1.334 | <0.0001* |
| Boy | -0.047 (-0.07 – -0.03) | 0.954 | 1.113 | <0.0001* | .. | .. | .. | .. | .. | .. | .. | .. |
| Non-binary | 0.116 (0.03 – 0.20) | 1.123 | 1.269 | 0.006 | .. | .. | .. | .. | .. | .. | .. | .. |
| 2016*14 years old | 0.136 (0.06 – 0.21) | 1.146 | 1.289 | 0.001 | 0.164 (0.05 – 0.27) | 1.178 | 1.273 | 0.004 | 0.126 (0.02 – 0.24) | 1.135 | 1.250 | 0.026 |
| 2018*14 years old | 0.109 (0.03 – 0.19) | 1.116 | 1.263 | 0.006 | 0.156 (0.05 – 0.27) | 1.169 | 1.265 | 0.006 | 0.087 (-0.02 – 0.20) | 1.091 | 1.212 | 0.122 |
| 2020*14 years old | -0.023 (-0.10 – 0.06) | 0.977 | 1.136 | 0.575 | -0.031 (-0.15 – 0.08) | 0.969 | 1.088 | 0.592 | 0.006 (-0.11 – 0.12) | 1.006 | 1.135 | 0.920 |
| 2022*14 years old | 0.081 (0.00 – 0.16) | 1.084 | 1.234 | 0.044 | 0.153 (0.04 – 0.27) | 1.165 | 1.262 | 0.009 | 0.039 (-0.07 – 0.15) | 1.040 | 1.166 | 0.488 |
| 2016*14 years old | -0.024 (-0.10 – 0.06) | 0.977 | 1.135 | 0.556 | -0.075 (-0.19 – 0.04) | 0.928 | 1.049 | 0.187 | 0.025 (-0.09 – 0.14) | 1.025 | 1.153 | 0.662 |
| 2018*15 years old | 0.040 (-0.04 – 0.12) | 1.041 | 1.195 | 0.321 | 0.012 (-0.10 – 0.12) | 1.012 | 1.127 | 0.839 | 0.068 (-0.05 – 0.18) | 1.071 | 1.194 | 0.238 |
| 2020*15 years old | 0.050 (-0.03 – 0.13) | 1.052 | 1.205 | 0.231 | 0.067 (-0.05 – 0.18) | 1.069 | 1.178 | 0.269 | 0.056 (-0.06 – 0.17) | 1.058 | 1.182 | 0.346 |
| 2022*15 years old | 0.054 (-0.03 – 0.13) | 1.056 | 1.208 | 0.185 | 0.106 (-0.01 – 0.22) | 1.112 | 1.216 | 0.073 | 0.029 (-0.08 – 0.14) | 1.030 | 1.157 | 0.612 |

*Note:* *: Significant after Bonferroni correction (<0.00043).

Table S9. Model predictions for stress/trauma event incidence throughout the study period. Fixed effects of survey year (indexed to 2023), gender and their predictive power on incidence of stress/trauma events are presented. Model estimates logistic mixed-effects models (OR) are presented with CI, alongside survey score equivalents to the cumulative survey scores, and significance value (*p*). All genders, and gender stratified models for girls and boys are presented separately.

|  |  | **All genders** | | **Girls** | | **Boys** | |
| --- | --- | --- | --- | --- | --- | --- | --- |
| **Stress/Trauma** | **Predictor** | **OR (CI)** | **p value** | **OR (CI)** | **p value** | **OR (CI)** | **p value** |
| Sickness | 2016 | 0.603 (0.52 – 0.70) | <0.0001* | 0.685 (0.56 - 0.84) | 0.0002* | 0.523 (0.43 - 0.64) | <0.0001* |
| Sickness | 2018 | 0.694 (0.61 – 0.80) | <0.0001* | 0.680 (0.56 - 0.83) | 0.0001* | 0.700 (0.58 - 0.84) | 0.0002* |
| Sickness | 2020 | 0.645 (0.56 – 0.74) | <0.0001* | 0.720 (0.59 - 0.89) | 0.002 | 0.581 (0.48 - 0.71) | <0.0001* |
| Sickness | 2022 | 2.089 (1.87 – 2.33) | <0.0001* | 2.084 (1.77 - 2.46) | <0.0001* | 2.084 (1.79 - 2.43) | <0.0001* |
| Sickness | 14 years old | 1.037 (0.94 – 1.14) | 0.452 | 1.035 (0.90 - 1.19) | 0.641 | 1.040 (0.91 - 1.19) | 0.559 |
| Sickness | 15 years old | 1.076 (0.98 – 1.18) | 0.137 | 1.185 (1.03 - 1.36) | 0.018 | 0.991 (0.86 - 1.14) | 0.896 |
| Sickness | Boys | 1.045 (0.97 – 1.13) | 0.277 | .. | .. | .. | .. |
| Sickness | Non-binary | 1.838 (1.48 – 2.28) | <0.0001* | .. | .. | .. | .. |
| Bad grade | 2016 | 1.039 (0.97 – 1.11) | 0.283 | 0.962 (0.87 - 1.06) | 0.436 | 1.122 (1.01 - 1.24) | 0.026 |
| Bad grade | 2018 | 0.850 (0.79 – 0.91) | <0.0001* | 0.759 (0.69 - 0.84) | <0.0001* | 0.956 (0.86 - 1.06) | 0.397 |
| Bad grade | 2020 | 0.693 (0.64 – 0.75) | <0.0001* | 0.656 (0.59 - 0.73) | <0.0001* | 0.729 (0.65 - 0.81) | <0.0001* |
| Bad grade | 2022 | 1.184 (1.11 – 1.27) | <0.0001* | 1.140 (1.03 - 1.26) | 0.009 | 1.240 (1.12 - 1.37) | <0.0001* |
| Bad grade | 14 years old | 1.176 (1.12 – 1.24) | <0.0001* | 1.205 (1.12 - 1.30) | <0.0001* | 1.150 (1.07 - 1.24) | <0.0004* |
| Bad grade | 15 years old | 1.110 (1.05 – 1.17) | 0.0002* | 1.138 (1.05 - 1.23) | 0.001 | 1.085 (1.00 - 1.17) | 0.042 |
| Bad grade | Boys | 0.900 (0.86 – 0.94) | <0.0001* | .. | .. | .. | .. |
| Bad grade | Non-binary | 1.187 (1.01 – 1.40) | 0.039 | .. | .. | .. | .. |
| Breakup | 2016 | 0.806 (0.70 – 0.92) | 0.002 | 0.682 (0.57 - 0.82) | <0.0001* | 0.954 (0.78 - 1.17) | 0.645 |
| Breakup | 2018 | 0.754 (0.66 – 0.87) | <0.0001* | 0.675 (0.56 - 0.81) | <0.0001* | 0.834 (0.68 - 1.03) | 0.087 |
| Breakup | 2020 | 0.921 (0.80 – 1.05) | 0.229 | 0.894 (0.75 - 1.07) | 0.230 | 0.938 (0.76 - 1.15) | 0.545 |
| Breakup | 2022 | 0.747 (0.65 – 0.86) | <0.0001* | 0.640 (0.53 - 0.78) | <0.0001* | 0.838 (0.68 - 1.03) | 0.097 |
| Breakup | 14 years old | 0.811 (0.74 – 1.89) | 0.648 | 1.070 (0.93 - 1.24) | 0.360 | 0.913 (0.78 - 1.06) | 0.242 |
| Breakup | 15 years old | 0.927 (0.83 – 1.03) | 0.160 | 1.005 (0.87 -1.17) | 0.947 | 0.859 (0.73 - 1.01) | 0.060 |
| Breakup | Boys | 0.811 (0.74 – 0.89) | <0.0001* | .. | .. | .. | .. |
| Breakup | Non-binary | 1.885 (1.47 – 2.43) | <0.0001* | .. | .. | .. | .. |
| Argument with parents | 2016 | 0.671 (0.61 – 0.74) | <0.0001* | 0.557 (0.49 - 0.64) | <0.0001* | 0.834 (0.72 - 0.97) | 0.017 |
| Argument with parents | 2018 | 0.615 (0.56 – 0.68) | <0.0001* | 0.486 (0.42 - 0.56) | <0.0001* | 0.811 (0.70 - 0.94) | 0.007 |
| Argument with parents | 2020 | 1.111 (1.01 – 1.22) | 0.025 | 1.039 (0.92 - 1.17) | 0.535 | 1.159 (1.00 - 1.34) | 0.046 |
| Argument with parents | 2022 | 1.013 (0.93 – 1.11) | 0.776 | 0.978 (0.87 - 1.10) | 0.719 | 1.017 (0.88 - 1.18) | 0.823 |
| Argument with parents | 14 years old | 1.129 (1.05 – 1.22) | 0.001 | 1.212 (1.10 - 1.34) | <0.0001* | 1.037 (0.93 - 1.16) | 0.525 |
| Argument with parents | 15 years old | 1.098 (1.02 – 1.18) | 0.014 | 1.191 (1.08 - 1.32) | 0.001 | 0.995 (0.89 – 1.12) | 0.931 |
| Argument with parents | Boys | 0.665 (0.63 – 0.71) | <0.0001* | .. | .. | .. | .. |
| Argument with parents | Non-binary | 1.353 (1.12 – 1.63) | 0.002 | .. | .. | .. | .. |
| Argument among parents | 2016 | 0.722 (0.63 – 0.83) | <0.0001* | 0.678 (0.57 - 0.81) | <0.0001* | 0.792 (0.64 - 0.98) | 0.030 |
| Argument among parents | 2018 | 0.659 (0.58 – 0.76) | <0.0001* | 0.652 (0.55 - 0.78) | <0.0001* | 0.666 (0.53 - 0.83) | 0.0003* |
| Argument among parents | 2020 | 0.953 (0.84 – 1.09) | 0.464 | 0.939 (0.79 - 1.11) | 0.465 | 0.977 (0.79 - 1.20) | 0.827 |
| Argument among parents | 2022 | 1.037 (0.92 – 1.17) | 0.569 | 1.102 (0.94 - 1.30) | 0.236 | 0.916 (0.75 - 1.13) | 0.404 |
| Argument among parents | 14 years old | 1.069 (0.97 – 1.18) | 0.194 | 1.144 (1.00 - 1.30) | 0.045 | 0.988 (0.84 - 1.16) | 0.887 |
| Argument among parents | 15 years old | 0.994 (0.90 – 1.10) | 0.914 | 1.069 (0.94 - 1.22) | 0.330 | 0.890 (0.75 - 1.05) | 0.172 |
| Argument among parents | Boys | 0.583 (0.54 – 0.64) | <0.0001* | .. | .. | .. | .. |
| Argument among parents | Non-binary | 1.206 (0.93 – 1.56) | 0.156 | .. | .. | .. | .. |
| Parental unemployment | 2016 | 1.262 (1.07 – 1.49) | 0.006 | 1.208 (0.96 - 1.52) | 0.107 | 1.362 (1.06 - 1.74) | 0.014 |
| Parental unemployment | 2018 | 1.186 (1.00 – 1.40) | 0.047 | 1.169 (0.93 - 1.47) | 0.186 | 1.236 (0.96 - 1.59) | 0.100 |
| Parental unemployment | 2020 | 3.108 (2.68 – 3.60) | <0.0001* | 3.121 (2.55 - 3.83) | <0.0001* | 3.227 (2.59 - 4.03) | <0.0001* |
| Parental unemployment | 2022 | 1.594 (1.36 – 1.87) | <0.0001* | 1.653 (1.32 - 2.06) | <0.0001* | 1.532 (1.20 - 1.96) | 0.001 |
| Parental unemployment | 14 years old | 1.149 (1.03 – 1.28) | 0.014 | 1.147 (0.98 - 1.34) | 0.080 | 1.111 (0.94 - 1.31) | 0.206 |
| Parental unemployment | 15 years old | 1.245 (1.12 – 1.39) | <0.0001* | 1.277 (1.10 - 1.49) | 0.002 | 1.178 (1.00 - 1.39) | 0.050 |
| Parental unemployment | Boys | 0.810 (0.74 – 0.89) | <0.0001* | .. | .. | .. | .. |
| Parental unemployment | Non-binary | 1.447 (1.09 – 1.92) | 0.010 | .. | .. | .. | .. |
| Parental divorce | 2016 | 0.930 (0.77 – 1.12) | 0.451 | 0.856 (0.66 - 1.12) | 0.249 | 1.020 (0.78 - 1.34) | 0.885 |
| Parental divorce | 2018 | 0.878 (0.73 – 1.06) | 0.185 | 0.903 (0.70 - 1.17) | 0.442 | 0.849 (0.64 - 1.13) | 0.262 |
| Parental divorce | 2020 | 0.950 (0.78 – 1.15) | 0.608 | 0.881 (0.67 - 1.16) | 0.367 | 1.036 (0.78 - 1.37) | 0.807 |
| Parental divorce | 2022 | 0.806 (0.66 – 0.98) | 0.031 | 0.817 (0.62 - 1.08) | 0.149 | 0.787 (0.59 - 1.06) | 0.110 |
| Parental divorce | 14 years old | 0.986 (0.85 – 1.14) | 0.848 | 1.085 (0.88 - 1.34) | 0.456 | 0.906 (0.73 - 1.12) | 0.365 |
| Parental divorce | 15 years old | 1.036 (0.89 – 1.20) | 0.641 | 1.244 (1.01 - 1.53) | 0.040 | 0.845 (0.68 - 1.05) | 0.135 |
| Parental divorce | Boys | 0.871 (0.77 – 0.97) | 0.029 | .. | .. | .. | .. |
| Parental divorce | Non-binary | 1.501 (1.01 – 2.24) | 0.045 | .. | .. | .. | .. |
| Witness Domestic Violence | 2016 | 1.071 (0.83 – 1.39) | 0.602 | 1.139 (0.80 - 1.62) | 0.471 | 0.962 (0.65 - 1.42) | 0.843 |
| Witness Domestic Violence | 2018 | 1.207 (0.96 – 1.56) | 0.146 | 1.304 (0.92 - 1.84) | 0.132 | 1.056 (0.72 - 1.55) | 0.778 |
| Witness Domestic Violence | 2020 | 1.274 (0.99 – 1.65) | 0.066 | 1.448 (1.02 - 2.06) | 0.039 | 1.002 (0.67 - 1.50) | 0.992 |
| Witness Domestic Violence | 2022 | 1.070 (0.83 – 1.39) | 0.608 | 1.211 (0.85 - 1.73) | 0.294 | 0.843 (0.56 - 1.27) | 0.411 |
| Witness Domestic Violence | 14 years old | 1.251 (1.02 – 1.53) | 0.029 | 1.567 (1.19 - 2.07) | 0.001 | 0.986 (0.73 - 1.34) | 0.927 |
| Witness Domestic Violence | 15 years old | 1.502 (1.24 – 1.83) | <0.0001* | 1.832 (1.40 - 2.40) | <0.0001* | 1.135 (0.84 - 1.53) | 0.407 |
| Witness Domestic Violence | Boys | 0.680 (0.58 – 0.80) | <0.0001* | .. | .. | .. | .. |
| Witness Domestic Violence | Non-binary | 2.029 (1.32 – 3.11) | 0.001 | .. | .. | .. | .. |
| Subject of domestic violence | 2016 | 1.259 (0.96 – 1.66) | 0.098 | 1.423 (0.99 - 2.04) | 0.055 | 1.070 (0.69 - 1.66) | 0.761 |
| Subject of domestic violence | 2018 | 1.217 (0.92 – 1.60) | 0.164 | 1.120 (0.77 - 1.64) | 0.559 | 1.368 (0.90 - 2.08) | 0.141 |
| Subject of domestic violence | 2020 | 1.387 (1.05 – 1.82) | 0.020 | 1.504 (1.04 - 2.18) | 0.031 | 1.257 (0.81 - 1.94) | 0.302 |
| Subject of domestic violence | 2022 | 1.206 (0.92 – 1.59) | 0.181 | 1.234 (0.84 - 1.81) | 0.277 | 1.173 (0.76 - 1.81) | 0.468 |
| Subject of domestic violence | 14 years old | 1.544 (1.25 – 1.92) | <0.0001* | 1.969 (1.46 - 2.66) | <0.0001* | 1.173 (0.84 - 1.63) | 0.342 |
| Subject of domestic violence | 15 years old | 1.766 (1.43 – 2.18) | <0.0001* | 2.113 (1.57 - 2.85) | <0.0001* | 1.423 (1.03 - 1.96) | 0.030 |
| Subject of domestic violence | Boys | 0.690 (0.58 – 0.82) | <0.0001* | .. | .. | .. | .. |
| Subject of domestic violence | Non-binary | 2.135 (1.38 – 3.31) | 0.001 | .. | .. | .. | .. |
| Parental or sibling death | 2016 | 0.752 (0.56 – 1.02) | 0.066 | 0.915 (0.57 - 1.48) | 0.716 | 0.615 (0.41 - 0.91) | 0.016 |
| Parental or sibling death | 2018 | 0.736 (0.54 – 1.00) | 0.050 | 0.791 (0.48 - 1.30) | 0.354 | 0.662 (0.45 - 0.98) | 0.039 |
| Parental or sibling death | 2020 | 0.686 (0.50 – 0.95) | 0.022 | 0.461 (0.25 - 0.85) | 0.014 | 0.754 (0.51 - 1.12) | 0.157 |
| Parental or sibling death | 2022 | 0.713 (0.53 – 0.97) | 0.029 | 0.546 (0.31 - 0.96) | 0.037 | 0.729 (0.50 - 1.07) | 0.107 |
| Parental or sibling death | 14 years old | 1.186 (0.93 – 1.52) | 0.178 | 1.091 (0.73 - 1.62) | 0.666 | 1.212 (0.88 - 1.68) | 0.248 |
| Parental or sibling death | 15 years old | 1.224 (0.95 – 1.57) | 0.112 | 0.765 (0.49 - 1.19) | 0.230 | 1.449 (1.06 - 1.99) | 0.022 |
| Parental or sibling death | Boys | 1.716 (1.39 – 2.12) | <0.0001* | .. | .. | .. | .. |
| Parental or sibling death | Non-binary | 3.619 (2.15 – 6.09) | <0.0001* | .. | .. | .. | .. |
| Death of friend | 2016 | 0.898 (0.75 – 1.08) | 0.263 | 1.061 (0.82 - 1.38) | 0.658 | 0.722 (0.55 - 0.95) | 0.019 |
| Death of friend | 2018 | 1.087 (0.91 – 1.30) | 0.368 | 1.179 (0.91 - 1.52) | 0.207 | 0.978 (0.76 - 1.26) | 0.863 |
| Death of friend | 2020 | 1.129 (0.94 – 1.36) | 0.198 | 1.302 (1.00 - 1.69) | 0.049 | 0.868 (0.66 - 1.14) | 0.307 |
| Death of friend | 2022 | 1.110 (0.93 – 1.33) | 0.253 | 1.283 (0.99 - 1.66) | 0.058 | 0.878 (0.67 - 1.14) | 0.332 |
| Death of friend | 14 years old | 1.195 (1.04 – 1.37) | 0.011 | 1.169 (0.96 - 1.42) | 0.114 | 1.225 (1.00 - 1.50) | 0.053 |
| Death of friend | 15 years old | 1.378 (1.20 – 1.58) | <0.0001* | 1.457 (1.21 - 1.76) | <0.0001* | 1.296 (1.06 - 1.59) | 0.014 |
| Death of friend | Boys | 0.807 (0.72 – 0.90) | 0.0002* | .. | .. | .. | .. |
| Death of friend | Non-binary | 2.020 (1.48 – 2.74) | <0.0001* | .. | .. | .. | .. |
| Adult sexual abuse | 2016 | 0.473 (0.35 – 0.65) | <0.0001* | 0.444 (0.31 - 0.64) | <0.0001* | 0.511 (0.29 - 0.90) | 0.020 |
| Adult sexual abuse | 2018 | 0.591 (0.44 – 0.79) | 0.00042* | 0.588 (0.42 - 0.83) | 0.003 | 0.548 (0.31 - 0.96) | 0.034 |
| Adult sexual abuse | 2020 | 0.690 (0.52 – 0.92) | 0.012 | 0.650 (0.46 - 0.93) | 0.017 | 0.688 (0.40 - 1.18) | 0.176 |
| Adult sexual abuse | 2022 | 1.089 (0.85 – 1.39) | 0.499 | 1.066 (0.79 - 1.45) | 0.679 | 0.961 (0.59 - 1.55) | 0.870 |
| Adult sexual abuse | 14 years old | 1.472 (1.16 – 1.87) | 0.002 | 1.939 (1.42 - 2.66) | <0.0001* | 0.926 (0.61 - 1.42) | 0.724 |
| Adult sexual abuse | 15 years old | 1.993 (1.58 – 2.51) | <0.0001* | 2.733 (2.02 - 3.69) | <0.0001* | 1.009 (0.66 - 1.54) | 0.966 |
| Adult sexual abuse | Boys | 0.375 (0.31 – 0.46) | <0.0001* | .. | .. | .. | .. |
| Adult sexual abuse | Non-binary | 2.169 (1.48 – 3.18) | <0.0001* | .. | .. | .. | .. |
| Peer sexual abuse | 2016 | 0.612 (0.49 - 0.76) | <0.0001* | 0.652 (0.51 - 0.84) | 0.001 | 0.496 (0.32 - 0.78) | 0.002 |
| Peer sexual abuse | 2018 | 0.619 (0.50 - 0.77) | <0.0001* | 0.686 (0.54 - 0.88) | 0.003 | 0.425 (0.26 - 0.68) | 0.00044 |
| Peer sexual abuse | 2020 | 1.038 (0.85 – 1.27) | 0.708 | 1.206 (0.96 - 1.52) | 0.107 | 0.561 (0.36 - 0.88) | 0.013 |
| Peer sexual abuse | 2022 | 1.448 (1.21 – 1.73) | <0.0001* | 1.668 (1.35 - 2.06) | <0.0001* | 0.844 (0.57 - 1.25) | 0.399 |
| Peer sexual abuse | 14 years old | 1.990 (1.67 – 2.37) | <0.0001* | 2.301 (1.87 - 2.83) | <0.0001* | 1.260 (0.87 - 1.83) | 0.222 |
| Peer sexual abuse | 15 years old | 2.626 (2.22 – 3.11) | <0.0001* | 3.107 (2.54 - 3.80) | <0.0001* | 1.509 (1.05 - 2.17) | 0.026 |
| Peer sexual abuse | Boys | 0.212 (0.18 – 0.25) | <0.0001* | .. | .. | .. | .. |
| Peer sexual abuse | Non-binary | 1.227 (0.90 – 1.68) | 0.202 | .. | .. | .. | .. |
| Accident | 2016 | 0.633 (0.56 - 0.72) | <0.0001* | 0.605 (0.50 - 0.74) | <0.0001* | 0.646 (0.55 - 0.76) | <0.0001* |
| Accident | 2018 | 0.800 (0.71 - 0.90) | 0.0001* | 0.690 (0.57 - 0.83) | 0.0001* | 0.866 (0.75 - 1.00) | 0.054 |
| Accident | 2020 | 0.934 (0.83 – 1.05) | 0.237 | 0.923 (0.77 - 1.12) | 0.389 | 0.937 (0.81 - 1.09) | 0.388 |
| Accident | 2022 | 0.909 (0.81 – 1.02) | 0.096 | 0.929 (0.78 - 1.11) | 0.424 | 0.885 (0.76 - 1.03) | 0.105 |
| Accident | 14 years old | 0.928 (0.85 – 1.01) | 0.091 | 0.968 (0.84 - 1.12) | 0.655 | 0.898 (0.80 - 1.01) | 0.061 |
| Accident | 15 years old | 0.850 (0.78 - 0.93) | 0.00043* | 0.926 (0.80 - 1.07) | 0.301 | 0.806 (0.72 - 0.91) | <0.0003* |
| Accident | Boys | 1.616 (1.50 – 1.74) | <0.0001* | .. | .. | .. | .. |
| Accident | Non-binary | 1.934 (1.52 – 2.46) | <0.0001* | .. | .. | .. | .. |

*Note:* *: Significant after Bonferroni correction (<0.00043).

Table S10. Bioecological model predictions for adolescent mental health outcomes (2023 Reference Year). Results are presented separately and sequentially for depressive symptom scores, anxiety symptom scores and hostility scores. Fixed effects of survey year and stepwise selected risk and protective factors across individual, home, and peer/school environments. Model estimates from beta mixed-effects models (β) are presented with CI, alongside survey score equivalents to the cumulative survey scores, and significance value (*p*). All genders, and gender stratified models for girls and boys are presented separately.

| Depressive symptoms (score range 10-40) | | | | | | | | | | | | |
| --- | --- | --- | --- | --- | --- | --- | --- | --- | --- | --- | --- | --- |
|  | All genders | | | | Girls | | | | Boys | | | |
| **Predictors** | **β (CI)** | **Log odds ratio** | **Survey score equivalent** | **p value** | **β (CI)** | **Log odds ratio** | **Survey score equivalent** | **p value** | **β (CI)** | **Log odds ratio** | **Survey score equivalent** | **p value** |
| 2016 | -0.156 (-0.20 - -0.11) | 0.855 | 29.919 | <0.0001***** | 0.925 (0.67 - 1.18) | 2.521 | 34.224 | <0.0001***** | -0.246 (-0.29 - -0.20) | 0.782 | 21.332 | <0.0001***** |
| 2018 | -0.175 (-0.22 - -0.13) | 0.839 | 29.792 | <0.0001***** | 0.596 (0.34 - 0.85) | 1.815 | 32.537 | <0.0001***** | -0.100 (-0.14 - -0.06) | 0.905 | 22.375 | <0.0001***** |
| 2020 | 0.155 (0.11 - 0.20) | 1.167 | 31.884 | <0.0001***** | 0.634 (0.37 - 0.89) | 1.884 | 32.744 | <0.0001***** | 0.106 (0.06 - 0.15) | 1.111 | 23.892 | <0.0001***** |
| 2022 | 0.132 (0.09 - 0.18) | 1.141 | 31.750 | <0.0001***** | 0.403 (0.15 - 0.66) | 1.496 | 31.399 | 0.002 | 0.049 (0.01 - 0.09) | 1.050 | 23.469 | 0.028 |
| Boy | -0.649 (-0.70 - -0.60) | 0.523 | 26.410 | <0.0001***** | .. | .. | .. | .. | .. | .. | .. | .. |
| Non-binary | 0.290 (0.15 - 0.43) | 1.337 | 32.661 | <0.0001***** | .. | .. | .. | .. | .. | .. | .. | .. |
| Parental social support | -0.119 (-0.12 - -0.12) | 0.888 | 30.164 | <0.0001* | -0.108 (-0.12 - -0.10) | 0.898 | 27.967 | <0.0001* | -0.091 (-0.1 - -0.09) | 0.913 | 22.438 | <0.0001***** |
| Language spoken at home | .. | .. | .. | .. | .. | .. | .. | .. | 0.080 (0.04 - 0.11) | 1.083 | 23.697 | <0.0001***** |
| Watching videos or films | 0.085 (0.08 - 0.09) | 1.089 | 31.466 | <0.0001* | 0.091 (0.08 - 0.10) | 1.096 | 29.371 | <0.0001* | 0.075 (0.07 - 0.08) | 1.077 | 23.661 | <0.0001***** |
| Gaming alone | 0.029 (0.02 - 0.04) | 1.030 | 31.121 | <0.0001* | .. | .. | .. | .. | 0.036 (0.03 - 0.04) | 1.036 | 23.372 | <0.0001***** |
| Social media | 0.063 (0.06 - 0.07) | 1.065 | 31.327 | <0.0001* | 0.087 (0.08 - 0.10) | 1.091 | 29.339 | <0.0001* | 0.021 (0.01 - 0.03) | 1.022 | 23.267 | <0.0001***** |
| Other screen usage | .. |  | .. | .. | 0.069 (0.06 - 0.08) | 1.071 | 29.214 | <0.0001* | .. | .. | .. | .. |
| Sickness | 0.310 (0.26 - 0.35) | 1.363 | 32.770 | <0.0001* | .. | .. | .. | .. | .. | .. | .. | .. |
| Bad grade | 0.360 (0.33 - 0.38) | 1.433 | 33.040 | <0.0001* | 0.331 (0.29 - 0.37) | 1.392 | 30.950 | <0.0001* | 0.360 (0.32 - 0.40) | 1.433 | 25.796 | <0.0001***** |
| Breakup | 0.333 (0.30 - 0.37) | 1.395 | 32.895 | <0.0001* | 0.173 (0.06 - 0.28) | 1.189 | 29.927 | 0.002 | 0.311 (0.26 - 0.36) | 1.365 | 25.432 | <0.0001***** |
| Argument with parents | .. |  | .. | .. | 0.462 (0.41 - 0.51) | 1.587 | 31.756 | <0.0001* | 0.426 (0.37 - 0.48) | 1.531 | 26.290 | <0.0001***** |
| Argument among parents | 0.341 (0.29 - 0.39) | 1.406 | 32.940 | <0.0001* | .. | .. | .. | .. | 0.210 (0.13 - 0.29) | 1.233 | 24.670 | <0.0001***** |
| Parental unemployment | 0.194 (0.14 - 0.25) | 1.214 | 32.117 | <0.0001* | 0.143 (0.07 - 0.21) | 1.153 | 29.719 | <0.0001* | 0.290 (0.22 - 0.36) | 1.337 | 25.275 | <0.0001***** |
| Witness domestic violence | 0.176 (0.08 - 0.27) | 1.193 | 32.011 | 0.0003* | .. | .. | .. | .. | .. | .. | .. | .. |
| Peer sexual abuse | 0.641 (0.57 - 0.71) | 1.899 | 34.431 | <0.001* | 0.650 (0.57 - 0.73) | 1.916 | 32.836 | <0.0001* | .. | .. | .. | .. |
| Accident | .. | .. | .. | .. | .. | .. | .. | .. | 0.196 (0.14 - 0.25) | 1.216 | 24.567 | <0.0001***** |
| Boys*2016 | -0.081 (-0.14 - -0.02) | 0.922 | 30.418 | 0.010 | .. | .. | .. | .. | .. | .. | .. | .. |
| Boys*2018 | 0.074 (0.01 - 0.14) | 1.077 | 31.400 | 0.019 | .. | .. | .. | .. | .. | .. | .. | .. |
| Boys*2020 | -0.031 (-0.10 - 0.03) | 0.970 | 30.741 | 0.353 | .. | .. | .. | .. | .. | .. | .. | .. |
| Non-binary*2020 | -0.510 (-0.73 - -0.29) | 0.600 | 27.430 | <0.0001* | .. | .. | .. | .. | .. | .. | .. | .. |
| Boys*2022 | -0.098 (-0.16 - -0.03) | 0.906 | 30.303 | 0.002 | .. | .. | .. | .. | .. | .. | .. | .. |
| Non-binary*2022 | -0.015 (-0.19 - 0.17) | 0.985 | 30.843 | 0.873 | .. | .. | .. | .. | .. | .. | .. | .. |
| Parental social support*2016 | .. | .. | .. | .. | -0.062 (-0.08 - -0.05) | 0.940 | 28.298 | <0.0001* | .. | .. | .. | .. |
| Parental social support*2018 | .. | .. | .. | .. | -0.043 (-0.06 - -0.03) | 0.958 | 28.432 | <0.0001***** | .. | .. | .. | .. |
| Parental social support*2020 | .. | .. | .. | .. | -0.028 (-0.04 - -0.01) | 0.972 | 28.535 | 0.0002***** | .. | .. | .. | .. |
| Parental social support*2022 | .. | .. | .. | .. | -0.016 (-0.03 - 0.00) | 0.985 | 28.626 | 0.033 | .. | .. | .. | .. |
| 2016*Breakup | .. | .. | .. | .. | 0.353 (0.20 - 0.51) | 1.423 | 31.090 | <0.0001***** | .. | .. | .. | .. |
| 2018*Breakup | .. | .. | .. | .. | 0.072 (-0.08 - 0.23) | 1.074 | 29.235 | 0.360 | .. | .. | .. | .. |
| 2020*Breakup | .. | .. | .. | .. | 0.133 (-0.02 - 0.29) | 1.142 | 29.652 | 0.089 | .. | .. | .. | .. |
| 2022*Breakup | .. | .. | .. | .. | 0.136 (-0.02 - 0.29) | 1.146 | 29.677 | 0.078 | .. | .. | .. | .. |
| Anxiety symptoms (score range 5-20) | | | | | | | | | | | | |
|  | All genders | | | | Girls | | | | Boys | | | |
| **Predictors** | **β (CI)** | **Log odds ratio** | **Survey score equivalent** | **p value** | **β (CI)** | **Log odds ratio** | **Survey score equivalent** | **p value** | **β (CI)** | **Log odds ratio** | **Survey score equivalent** | **p value** |
| 2016 | .. | .. | .. | .. | 0.879 (0.60 - 1.16) | 2.408 | 15.711 | <0.0001***** | .. | .. | .. | .. |
| 2018 | .. | .. | .. | .. | 0.416 (0.13 - 0.70) | 1.516 | 14.169 | 0,004 | .. | .. | .. | .. |
| 2020 | .. | .. | .. | .. | 0.693 (0.40 - 0.98) | 2.000 | 15.121 | <0.0001***** | .. | .. | .. | .. |
| 2022 | .. | .. | .. | .. | 0.610 (0.33 - 0.89) | 1.841 | 14.843 | <0.0001***** | .. | .. | .. | .. |
| 14-years old | .. | .. | .. | .. | 0.123 (0.09 - 0.16) | 1.130 | 13.095 | <0.0001***** | -0.085 (-0.12 - -0.05) | 0.918 | 10.198 | <0.001***** |
| 15-years old | .. | .. | .. | .. | 0.133 (0.10 - 0.17) | 1.143 | 13.136 | <0.0001***** | -0.117 (-0.15 - -0.08) | 0.890 | 10.091 | <0.001***** |
| Boy | -0.857 (-0.88 - -0.84) | 0.424 | 11.233 | <0.0001* | .. | .. | .. | .. | .. | .. | .. | .. |
| Non-binary | 0.064 (-0.01 - 0.14) | 1.066 | 14.616 | 0.112 | .. | .. | .. | .. | .. | .. | .. | .. |
| Parental support | -0.075 (-0.08 - -0.07) | 0.928 | 14.127 | <0.0001* | -0.052 (-0.06 - -0.04) | 0.949 | 12.441 | <0.0001* | -0.059 (-0.06 - -0.05) | 0.943 | 10.288 | <0.001***** |
| Language spoken at home | .. | .. | .. | .. | -0.101 (-0.14 - -0.06) | 0.904 | 12.258 | <0.0001* | .. | .. | .. | .. |
| Watching videos or films | 0.063 (0.06 - 0.07) | 1.066 | 14.615 | <0.0001* | 0.058 (0.05 - 0.07) | 1.060 | 12.855 | <0.0001* | 0.055 (0.05 - 0.06) | 1.056 | 10.683 | <0.001***** |
| Gaming with friends | .. |  |  | .. | 0.018 (0.00 - 0.03) | 1.019 | 12.705 | 0.018 | 0.016 (0.01 - 0.02) | 1.016 | 10.546 | <0.001***** |
| Social media | 0.060 (0.05 - 0.07) | 1.062 | 14.603 | <0.0001* | 0.068 (0.06 - 0.08) | 1.071 | 12.893 | <0.0001* | 0.030 (0.02 - 0.04) | 1.031 | 10.597 | <0.001***** |
| Other screen usage | .. |  |  | .. | 0.066 (0.05 - 0.08) | 1.068 | 12.884 | <0.0001* | 0.025 (0.01 - 0.04) | 1.026 | 10.580 | <0.001***** |
| Sickness | 0.428 (0.38 - 0.47) | 1.534 | 15.797 | <0.0001* | 0.414 (0.34 - 0.48) | 1.512 | 14.160 | <0.0001* | 0.338 (0.27 - 0.40) | 1.402 | 11.713 | <0.001***** |
| Bad grade | 0.274 (0.25 - 0.30) | 1.315 | 15.317 | <0.0001* | 0.295 (0.26 - 0.33) | 1.343 | 13.732 | <0.0001* | 0.228 (0.19 - 0.26) | 1.256 | 11.305 | <0.001***** |
| Breakup | 0.237 (0.20 - 0.27) | 1.267 | 15.197 | <0.0001* | 0.141 (0.02 - 0.26) | 1.151 | 13.162 | 0.019 | 0.199 (0.15 - 0.25) | 1.220 | 11.200 | <0.001***** |
| Argument with parents | 0.308 (0.27 - 0.35) | 1.361 | 15.427 | <0.0001* | 0.242 (0.13 - 0.35) | 1.274 | 13.539 | <0.0001* | 0.331 (0.27 - 0.39) | 1.392 | 11.685 | <0.001***** |
| Argument among parents | .. | .. | .. | .. | 0.250 (0.10 - 0.40) | 1.284 | 13.566 | <0.0001* | 0.127 (0.04 - 0.21) | 1.136 | 10.942 | 0.003 |
| Parental unemployment | .. | .. | .. | .. | -0.288 (-0.49 - -0.08) | 0.750 | 11.562 | 0.006 | .. | .. | .. | .. |
| Witness domestic violence | .. | .. | .. | .. | .. | .. | .. |  | 0.187 (0.03 - 0.34) | 1.206 | 11.158 | 0.018 |
| Death of a friend | 0.256 (0.19 - 0.32) | 1.292 | 15.259 | <0.0001* | 0.174 (0.08 - 0.26) | 1.190 | 13.286 | 0.0001* | 0.328 (0.23 - 0.43) | 1.388 | 11.674 | <0.001***** |
| Adult sexual abuse | .. | .. | .. | .. | 0,091 (-0.05 - 0.23) | 1.095 | 12.976 | 0.1922 | .. | .. | .. | .. |
| Peer sexual abuse | 0.540 (0.46 - 0.61) | 1.716 | 16.128 | <0.0001* | 0.468 (0.38 - 0.55) | 1.596 | 14.351 | < 0.0001* | .. | .. | .. | .. |
| Accident | .. | .. | .. | .. | 0.250 (0.18 - 0.32) | 1.284 | 13.566 | <0.0001* | 0.272 (0.22 - 0.33) | 1.313 | 11.468 | <0.001***** |
| Parental social support*2016 | .. | .. | .. | .. | -0.060 (-0.08 - -0.04) | 0.942 | 12.411 | <0.0001* | .. | .. | .. | .. |
| Parental social support*2018 | .. | .. | .. | .. | -0.035 (-0.05 - -0.02) | 0.966 | 12.505 | <0.0001* | .. | .. | .. | .. |
| Parental social support*2020 | .. | .. | .. | .. | -0.044 (-0.06 - -0.03) | 0.957 | 12.470 | <0.0001* | .. | .. | .. | .. |
| Parental social support*2022 | .. | .. | .. | .. | -0.033 (-0.05 - -0.02) | 0.968 | 12.513 | <0.0001* | .. | .. | .. | .. |
| 2016*Breakup | .. | .. | .. | .. | 0.229 (0.07 - 0.39) | 1.257 | 13.489 | 0.0051 | .. | .. | .. | .. |
| 2018*Breakup | .. | .. | .. | .. | 0.062 (-0.10 - 0.22) | 1.064 | 12.870 | 0.4453 | .. | .. | .. | .. |
| 2020*Breakup | .. | .. | .. | .. | 0.079 (-0.08 - 0.24) | 1.082 | 12.931 | 0.3318 | .. | .. | .. | .. |
| 2022*Breakup | .. | .. | .. | .. | 0.130 (-0.03 - 0.29) | 1.138 | 13.121 | 0.1096 | .. | .. | .. | .. |
| 2016*Argument with parents | .. | .. | .. | .. | -0.028 (-0.19 - 0.14) | 0.972 | 12.531 | 0.7402 | .. | .. | .. | .. |
| 2018*Argument with parents | .. | .. | .. | .. | -0.179 (-0.35 - -0.01) | 0.836 | 11.968 | 0.0436 | .. | .. | .. | .. |
| 2020*Argument with parents | .. | .. | .. | .. | -0.175 (-0.33 - -0.02) | 0.840 | 11.982 | 0.0269 | .. | .. | .. | .. |
| 2022*Argument with parents | .. | .. | .. | .. | 0.045 (-0.11 - 0.20) | 1.046 | 12.804 | 0.5650 | .. | .. | .. | .. |
| 2016*Argument among parents | .. | .. | .. | .. | 0.014 (-0.20 - 0.23) | 1.014 | 12.690 | 0.8964 | .. | .. | .. | .. |
| 2018*Argument among parents | .. | .. | .. | .. | 0.086 (-0.13 - 0.31) | 1.090 | 12.960 | 0.4406 | .. | .. | .. | .. |
| 2020*Argument among parents | .. | .. | .. | .. | -0.106 (-0.32 - 0.11) | 0.900 | -3.137 | 0.3304 | .. | .. | .. | .. |
| 2022*Argument among parents | .. | .. | .. | .. | -0.078 (-0.28 - 0.12) | 0.925 | -2.310 | 0.4485 | .. | .. | .. | .. |
| 2016*Parental unemployment | .. | .. | .. | .. | 0.325 (0.06 - 0.59) | 1.384 | 9.537 | 0.0174 | .. | .. | .. | .. |
| 2018*Parental unemployment | .. | .. | .. | .. | 0.440 (0.17 - 0.71) | 1.553 | 12.801 | 0.0015 | .. | .. | .. | .. |
| 2020*Parental unemployment | .. | .. | .. | .. | 0.411 (0.17 - 0.65) | 1.509 | 11.993 | 0.0008 | .. | .. | .. | .. |
| 2022*Parental unemployment | .. | .. | .. | .. | 0.364 (0.10 - 0.62) | 1.440 | 10.656 | 0.0061 | .. | .. | .. | .. |
| Hostility (score range 5-20) | | | | | | | | | | | | |
|  | All genders | | | | Girls | | | | Boys | | | |
| **Predictors** | **β (CI)** | **Log odds ratio** | **Survey score equivalent** | **p value** | **β (CI)** | **Log odds ratio** | **Survey score equivalent** | **p value** | **β (CI)** | **Log odds ratio** | **Survey score equivalent** | **p value** |
| 2016 | -0.127 (-0.16 - -0.09) | 0.881 | 11.383 | <0.0001***** | -0.251 (-0.33 - -0.17) | 0.777 | 11.857 | <0.0001***** | -0.001 (-0.07 - 0.06) | 0.999 | 10.082 | 0.978 |
| 2018 | -0.037 (-0.07 - 0.00) | 0.964 | 11.716 | 0.028 | -0.256 (-0.34 - -0.18) | 0.774 | 11.841 | <0.0001***** | 0.107 (0.04 - 0.17) | 1.112 | 10.450 | 0.002 |
| 2020 | 0.025 (-0.01 - 0.06) | 1.025 | 11.944 | 0.148 | -0.030 (-0.11 - 0.05) | 0.970 | 12.686 | 0.467 | 0.008 (-0.06 - 0.08) | 1.008 | 10.114 | 0.808 |
| 2021 | -0.059 (-0.09 - -0.03) | 0.943 | 11.633 | 0.0004***** | -0.097 (-0.18 - -0.02) | 0.907 | 12.434 | 0.019 | -0.072 (-0.14 - 0.00) | 0.930 | 9.845 | 0.036 |
| 14-years old | .. | 1.000 | .. | .. | .. | .. | .. | .. | -0.067 (-0.1 - -0.03) | 0.935 | 9.861 | 0.0001***** |
| 15-years old | .. | 1.000 | .. | .. | .. | .. | .. | .. | -0.068 (-0.1 - -0.03) | 0.934 | 9.860 | 0.0002***** |
| Boys | -0.216 (-0.24 - -0.19) | 0.806 | 11.058 | <0.0001***** | .. | .. | .. | .. | .. | .. | .. | .. |
| Non-binary | 0.119 (0.04 - 0.20) | 1.127 | 12.298 | 0.005 | .. | .. | .. | .. | .. | .. | .. | .. |
| Parental social support | -0.078 (-0.08 - -0.07) | 0.925 | 11.563 | <0.0001* | -0.092 (-0.10 - -0.09) | 0.912 | 12.452 | <0.0001* | -0.059 (-0.06 - -0.05) | 0.942 | 9.888 | <0.0001***** |
| Language spoken at home | -0.055 (-0.08 - -0.03) | 0.947 | 11.649 | <0.0001* | -0.081 (-0.12 - -0.04) | 0.922 | 12.496 | <0.0001* | .. | .. | .. | .. |
| Watching videos or films | 0.051 (0.04 - 0.06) | 1.053 | 12.043 | <0.0001* | 0.040 (0.02 - 0.06) | 1.040 | 12.947 | 0.001 | 0.039 (0.03 - 0.05) | 1.040 | 10.218 | <0.0001***** |
| Gaming with friends | 0.017 (0.01 - 0.03) | 1.017 | 11.917 | <0.0001* | 0.024 (-0.01 - 0.06) | 1.024 | 12.888 | 0.138 | 0.020 (0.01 - 0.03) | 1.020 | 10.152 | <0.0001***** |
| Social media | 0.096 (0.09 - 0.10) | 1.100 | 12.209 | <0.0001* | 0.109 (0.10 - 0.12) | 1.115 | 13.205 | <0.0001* | 0.081 (0.06 - 0.10) | 1.085 | 10.362 | <0.0001***** |
| Sickness | 0.090 (0.04 - 0.14) | 1.094 | 12.188 | 0.0002* | .. | .. | .. | .. | .. | .. | .. | .. |
| Bad grade | 0.293 (0.27 - 0.32) | 1.341 | 12.950 | <0.0001* | 0.334 (0.25 - 0.42) | 1.397 | 14.032 | <0.0001* | 0.278 (0.24 - 0.31) | 1.320 | 11.056 | <0.0001***** |
| Breakup | 0.378 (0.34 - 0.41) | 1.460 | 13.267 | <0.0001* | 0.380 (0.33 - 0.43) | 1.462 | 14.194 | <0.0001* | 0.339 (0.29 - 0.39) | 1.403 | 11.278 | <0.0001***** |
| Argument with parents | 0.811 (0.77 - 0.85) | 2.250 | 14.814 | <0.0001* | 0.782 (0.73 - 0.84) | 2.187 | 15.547 | <0.0001* | 0.824 (0.76 - 0.88) | 2.280 | 13.086 | <0.0001***** |
| Argument among parents | 0.184 (0.13 - 0.24) | 1.202 | 12.540 | <0.0001* | 0.173 (0.03 - 0.32) | 1.189 | 13.444 | 0.019 | 0.155 (0.07 - 0.24) | 1.168 | 10.620 | 0.0004 |
| Parental unemployment | .. | .. |  | .. | 0.132 (0.06 - 0.21) | 1.141 | 13.292 | 0.0004 | 0.155 (0.08 - 0.23) | 1.168 | 10.620 | 0.0001***** |
| Witness domestic violence | .. | .. |  | .. | -0.017 (-0.16 - 0.13) | 0.984 | 12.737 | 0.826 | .. | .. | .. | .. |
| Subject of domestic violence | 0.170 (0.07 - 0.27) | 1.186 | 12.489 | 0.001 | 0.159 (0.00 - 0.31) | 1.173 | 13.392 | 0.043 | .. | .. | .. | .. |
| Death of a friend | 0.257 (0.19 - 0.32) | 1.293 | 12.815 | <0.0001***** | 0.257 (0.02 - 0.49) | 1.293 | 13.752 | 0.031 | 0.291 (0.19 - 0.39) | 1.338 | 11.106 | <0.0001***** |
| Adult sexual abuse | .. | .. |  | .. | 0.241 (0.10 - 0.38) | 1.272 | 13.692 | 0.001 | .. | .. | .. | .. |
| Peer sexual abuse | 0.460 (0.38 - 0.54) | 1.584 | 13.567 | <0.0001***** | 0.478 (0.39 - 0.57) | 1.613 | 14.540 | <0.0001***** | .. | .. | .. | .. |
| Accident | .. | .. | .. | .. | 0.147 (-0.01 - 0.30) | 1.158 | 13.346 | 0.060 | 0.229 (0.17 - 0.29) | 1.258 | 10.882 | <0.0001***** |
| Social media*2016 | .. | .. | .. | .. | .. | .. | .. | .. | -0.018 (-0.04 - 0.01) | 0.983 | 10.026 | 0.200 |
| Social media*2018 | .. | .. | .. | .. | .. | .. | .. | .. | -0.001 (-0.03 - 0.03) | 0.999 | 10.082 | 0.940 |
| Social media*2020 | .. | .. | .. | .. | .. | .. | .. | .. | 0.014 (-0.01 - 0.04) | 1.014 | 10.133 | 0.327 |
| Social media*2022 | .. | .. | .. | .. | .. | .. | .. | .. | 0.003 (-0.02 - 0.03) | 1.003 | 10.095 | 0.840 |
| 2016*Game alone | .. | .. | .. | .. | -0.005 (-0.05 - 0.04) | 0.995 | 12.780 | 0.832 | .. | .. | .. | .. |
| 2018*Game alone | .. | .. | .. | .. | 0.035 (-0.02 - 0.08) | 1.035 | 12.928 | 0.179 | .. | .. | .. | .. |
| 2020*Game alone | .. | .. | .. | .. | -0.009 (-0.06 - 0.04) | 0.991 | 12.767 | 0.720 | .. | .. | .. | .. |
| 2022*Game alone | .. | .. | .. | .. | 0.051 (0.01 - 0.09) | 1.052 | 12.990 | 0.019 | .. | .. | .. | .. |
| 2016*Friend death | .. | .. | .. | .. | 0.079 (-0.22 - 0.38) | 1.082 | 13.095 | 0.605 | .. | .. | .. | .. |
| 2018*Friend death | .. | .. | .. | .. | 0.115 (-0.19 - 0.41) | 1.121 | 13.227 | 0.454 | .. | .. | .. | .. |
| 2020*Friend death | .. | .. | .. | .. | -0.117 (-0.43 - 0.19) | 0.889 | 12.359 | 0.459 | .. | .. | .. | .. |
| 2022*Friend death | .. | .. | .. | .. | -0.309 (-0.61 - -0.01) | 0.734 | 11.643 | 0.044 | .. | .. | .. | .. |
| 2016*Watching videos | .. | .. | .. | .. | 0.011 (-0.02 - 0.04) | 1.011 | 12.839 | 0.490 | .. | .. | .. | .. |
| 2018*Watching videos | .. | .. | .. | .. | 0.035 (0.00 - 0.07) | 1.035 | 12.930 | 0.026 | .. | .. | .. | .. |
| 2020*Watching videos | .. | .. | .. | .. | 0.031 (0.00 - 0.06) | 1.031 | 12.914 | 0.056 | .. | .. | .. | .. |
| 2022*Watching videos | .. | .. | .. | .. | 0.029 (0.00 - 0.06) | 1.029 | 12.906 | 0.071 | .. | .. | .. | .. |
| 2016*Accident | .. | .. | .. | .. | 0.240 (0.02 - 0.47) | 1.272 | 13.690 | 0.036 | .. | .. | .. | .. |
| 2018*Accident | .. | .. | .. | .. | 0.077 (-0.14 - 0.30) | 1.080 | 13.087 | 0.496 | .. | .. | .. | .. |
| 2020*Accident | .. | .. | .. | .. | 0.052 (-0.17 - 0.27) | 1.053 | 12.993 | 0.642 | .. | .. | .. | .. |
| 2022*Accident | .. | .. | .. | .. | 0.281 (0.06 - 0.50) | 1.324 | 13.837 | 0.010 | .. | .. | .. | .. |
| Bad grade*2016 | .. | .. | .. | .. | 0.013 (-0.10 - 0.13) | 1.013 | 12.848 | 0.821 | .. | .. | .. | .. |
| Bad grade*2018 | .. | .. | .. | .. | -0.001 (-0.12 - 0.12) | 0.999 | 12.794 | 0.983 | .. | .. | .. | .. |
| Bad grade*2020 | .. | .. | .. | .. | 0.007 (-0.12 - 0.13) | 1.007 | 12.824 | 0.915 | .. | .. | .. | .. |
| Bad grade*2022 | .. | .. | .. | .. | -0.145 (-0.26 - -0.03) | 0.865 | 12.256 | 0.013 | .. | .. | .. | .. |
| 2016* Argument among parents | .. | .. | .. | .. | 0.015 (-0.20 - 0.23) | 1.015 | 12.855 | 0.890 | .. | .. | .. | .. |
| 2018* Argument among parents | .. | .. | .. | .. | -0.099 (-0.32 - 0.12) | 0.906 | 12.429 | 0.372 | .. | .. | .. | .. |
| 2020* Argument among parents | .. | .. | .. | .. | -0.065 (-0.27 - 0.14) | 0.937 | 12.556 | 0.538 | .. | .. | .. | .. |
| 2022* Argument among parents | .. | .. | .. | .. | 0.186 (-0.01 - 0.38) | 1.205 | 13.491 | 0.062 | .. | .. | .. | .. |

*Note:* *: Significant after Bonferroni correction (<0.00043). Missing variables (··) were dropped from models during stepwise model selection using Likelihood-Ratio Tests and BIC optimisation, indicating they did not significantly improve model fit. All starting formulas for the models were the same and included all independent variables and their interactions with survey year.

Table S11. Bioecological model predictions for adolescent mental health outcomes (2016 Reference Year). Results are presented separately and sequentially for depressive symptom scores, anxiety symptom scores and hostility scores. Fixed effects of survey year and stepwise selected risk and protective factors across individual, home, and peer/school environments. Model estimates from beta mixed-effects models (β) are presented with CI, alongside survey score equivalents to the cumulative survey scores original scale, and significance value (*p*). All genders, and gender stratified models for girls and boys are presented separately.

|  | **Depressive symptoms (score range 10-40)** | | | | | | | | | | | |
| --- | --- | --- | --- | --- | --- | --- | --- | --- | --- | --- | --- | --- |
|  | **All genders** | | | | **Girls** | | | | **Boys** | | | |
| **Predictors** | **β (CI)** | **Odds ratio** | **Survey score equivalent** | **p value** | **β (CI)** | **Odds ratio** | **Survey score equivalent** | **p value** | **β (CI)** | **Odds ratio** | **Survey score equivalent** | **p value** |
| 2018 | .. | .. | .. | .. | -0.060 (-0.13 - 0.01) | 0.942 | 29.612 | 0.101 | 0.145 (0.10 - 0.19) | 1.157 | 22.514 | <0.0001* |
| 2020 | .. | .. | .. | .. | 0.319 (0.25 - 0.39) | 1.376 | 32.017 | <0.0001* | 0.355 (0.31 - 0.40) | 1.426 | 24.060 | <0.0001* |
| 2022 | .. | .. | .. | .. | 0.293 (0.22 - 0.37) | 1.340 | 31.862 | <0.0001* | 0.286 (0.24 - 0.33) | 1.332 | 23.553 | <0.0001* |
| 2023 | .. | .. | .. | .. | 0.242 (0.17 - 0.31) | 1.274 | 31.558 | <0.0001* | 0.247 (0.20 - 0.29) | 1.280 | 23.259 | <0.0001* |
| 14-years-old | .. | .. | .. | .. | 0.119 (0.09 - 0.15) | 1.127 | 30.792 | <0.0001* | .. | .. | .. | .. |
| 15-years-old | .. | .. | .. | .. | 0.160 (0.13 - 0.20) | 1.174 | 31.052 | <0.0001* | .. | .. | .. | .. |
| Boys | -0.684 (-0.71 - -0.66) | 0.505 | 26.261 | <0.0001* | .. | .. |  | .. | .. | .. | .. | .. |
| Non-binary | 0.286 (0.21 - 0.36) | 1.331 | 32.721 | <0.0001* | .. | .. |  | .. | .. | .. | .. | .. |
| Parental social support | -0.121 (-0.12 - -0.12) | 0.886 | 30.253 | <0.0001* | -0.134 (-0.14 - -0.13) | 0.874 | 29.099 | <0.0001* | -0.092 (-0.1 - -0.09) | 0.912 | 20.824 | <0.0001* |
| Language spoken at home | .. | .. | .. | .. | -0.059 (-0.1 - -0.02) | 0.943 | 29.616 | 0.002 | .. | .. | .. | .. |
| Watching videos or films | 0.080 (0.07 - 0.09) | 1.083 | 31.527 | <0.0001* | 0.089 (0.07 - 0.11) | 1.093 | 30.595 | <0.0001* | 0.075 (0.07 - 0.08) | 1.078 | 22.002 | <0.0001* |
| Gaming alone | 0.043 (0.04 - 0.05) | 1.044 | 31.299 | <0.0001* | 0.034 (0.00 - 0.07) | 1.035 | 30.241 | 0.042 | 0.037 (0.03 - 0.05) | 1.037 | 21.729 | <0.0001* |
| Social media | 0.061 (0.06 - 0.07) | 1.063 | 31.413 | <0.0001* | 0.080 (0.07 - 0.09) | 1.084 | 30.542 | <0.0001* | 0.022 (0.01 - 0.03) | 1.022 | 21.622 | <0.0001* |
| Other screen usage | .. | .. | .. | .. | 0.059 (0.05 - 0.07) | 1.060 | 30.401 | <0.0001* | .. |  |  | .. |
| Sickness | .. | .. | .. | .. | 0.711 (0.55 - 0.88) | 2.037 | 34.096 | .. | 0.168 (0.10 - 0.23) | 1.183 | 22.677 | <0.0001* |
| Bad grade | 0.354 (0.33 - 0.38) | 1.426 | 33.094 | <0.0001* | 0.310 (0.27 - 0.35) | 1.363 | 31.962 | <0.0001* | 0.355 (0.32 - 0.39) | 1.426 | 24.061 | <0.0001* |
| Breakup | 0.320 (0.29 - 0.35) | 1.378 | 32.909 | <0.0001* | 0.289 (0.24 - 0.34) | 1.335 | 31.838 | <0.0001* | 0.300 (0.25 - 0.35) | 1.350 | 23.653 | <0.0001* |
| Argument with parents | .. | .. | .. | .. | 0.648 (0.53 - 0.76) | 1.911 | 33.789 | <0.0001* | 0.413 (0.36 - 0.47) | 1.512 | 24.500 | <0.0001* |
| Argument among parents | 0.371 (0.32 - 0.42) | 1.450 | 33.181 | <0.0001* | 0.220 (0.15 - 0.29) | 1.246 | 31.421 | <0.0001* | 0.173 (0.09 - 0.26) | 1.189 | 22.713 | <0.0001* |
| Parental unemployment | 0.215 (0.16 - 0.27) | 1.240 | 32.326 | <0.0001* | 0.129 (0.06 - 0.20) | 1.138 | 30.854 | 0.0003* | 0.267 (0.19 - 0.34) | 1.306 | 23.410 | <0.0001* |
| Witness domestic violence | .. | .. | .. | .. | .. | .. | .. | .. | 0.319 (0.17 - 0.47) | 1.376 | 23.794 | <0.0001* |
| Subject of domestic violence | 0.124 (0.02 - 0.22) | 1.132 | 31.790 | 0.015 | .. | .. | .. | .. | .. | .. | .. | .. |
| Death of a friend | 0.336 (0.27 - 0.40) | 1.399 | 32.992 | <0.0001* | 0.385 (0.30 - 0.47) | 1.470 | 32.399 | <0.0001* | .. | .. | .. | .. |
| Peer sexual abuse | 0.647 (0.57 - 0.72) | 1.909 | 34.524 | <0.0001* | 0.558 (0.47 - 0.64) | 1.747 | 33.334 | <0.0001* | .. | .. | .. | .. |
| Accident | 0.204 (0.16 - 0.25) | 1.226 | 32.261 | <0.0001* | 0.194 (0.13 - 0.26) | 1.214 | 31.262 | <0.0001* | 0.182 (0.13 - 0.24) | .. | 22.779 | <0.0001* |
| 2018*Sickness | .. | .. | .. | .. | -0.072 (-0.30 - 0.16) | 0.931 | 29.531 | 0.545 | .. | .. | .. | .. |
| 2020*Sickness | .. | .. | .. | .. | -0.479 (-0.72 - -0.24) | 0.619 | 26.612 | 0.0001* | .. | .. | .. | .. |
| 2022*Sickness | .. | .. | .. | .. | -0.416 (-0.61 - -0.22) | 0.659 | 27.077 | <0.0001* | .. | .. | .. | .. |
| 2023*Sickness | .. | .. | .. | .. | -0.571 (-0.8 - -0.35) | 0.565 | 25.930 | <0.0001* | .. | .. | .. | .. |
| Argument with parents*2018 | .. | .. | .. | .. | -0.212 (-0.38 - -0.05) | 0.809 | 28.556 | 0.012 | .. | .. | .. | .. |
| Argument with parents*2020 | .. | .. | .. | .. | -0.373 (-0.52 - -0.22) | 0.689 | 27.399 | <0.0001* | .. | .. | .. | .. |
| Argument with parents*2022 | .. | .. | .. | .. | -0.322 (-0.47 - -0.17) | 0.725 | 27.767 | <0.0001***** | .. | .. | .. | .. |
| Argument with parents*2023 | .. | .. | .. | .. | -0.304 (-0.45 - -0.16) | 0.738 | 27.898 | <0.0001***** | .. | .. | .. | .. |
| 2018*Gaming alone | .. | .. | .. | .. | 0.062 (0.01 - 0.11) | 1.063 | 30.419 | 0.015 | .. | .. | .. | .. |
| 2020*Gaming alone | .. | .. | .. | .. | -0.016 (-0.06 - 0.03) | 0.984 | 29.909 | 0.513 | .. | .. | .. | .. |
| 2022*Gaming alone | .. | .. | .. | .. | 0.005 (-0.04 - 0.05) | 1.005 | 30.048 | 0.813 | .. | .. | .. | .. |
| 2023*Gaming alone | .. | .. | .. | .. | -0.007 (-0.05 - 0.04) | 0.993 | 29.969 | 0.768 | .. | .. | .. | .. |
| Watching videos*2018 | .. | .. | .. | .. | 0.017 (-0.01 - 0.04) | 1.018 | 30.129 | 0.213 | .. | .. | .. | .. |
| Watching videos*2020 | .. | .. | .. | .. | 0.006 (-0.02 - 0.03) | 1.006 | 30.057 | 0.658 | .. | .. | .. | .. |
| Watching videos*2022 | .. | .. | .. | .. | -0.003 (-0.03 - 0.02) | 0.997 | 29.992 | 0.822 | .. | .. | .. | .. |
| Watching videos*2023 | .. | .. | .. | .. | -0.040 (-0.07 - -0.01) | 0.961 | 29.748 | 0.007 | .. | .. | .. | .. |
|  | **Anxiety symptoms (score range 5-20)** | | | | | | | | | | | |
|  | **All genders** | | | | **Girls** | | | | **Boys** | | | |
| **Predictors** | **β (CI)** | **Odds ratio** | **Survey score equivalent** | **p value** | **β (CI)** | **Odds ratio** | **Survey score equivalent** | **p value** | **β (CI)** | **Odds ratio** | **Survey score equivalent** | **p value** |
| 2018 | .. | .. | .. | .. | -0.041 (-0.09 - 0.00) | 0.960 | 14.180 | 0.065 | .. | .. | .. | .. |
| 2020 | .. | .. | .. | .. | 0.069 (0.02 - 0.12) | 1.072 | 14.568 | 0.004 | .. | .. | .. | .. |
| 2022 | .. | .. | .. | .. | 0.209 (0.16 - 0.26) | 1.233 | 15.043 | <0.0001***** | .. | .. | .. | .. |
| 2023 | .. | .. | .. | .. | 0.146 (0.10 - 0.19) | 1.157 | 14.831 | <0.0001***** | .. | .. | .. | .. |
| 14-years-old | .. | .. | .. | .. | 0.122 (0.09 - 0.16) | 1.130 | 14.751 | <0.0001***** | -0.086 (-0.12 - -0.05) | 0.918 | 10.252 | <0.0001***** |
| 15-years-old | .. | .. | .. | .. | 0.134 (0.10 - 0.17) | 1.143 | 14.789 | <0.0001***** | -0.117 (-0.15 - -0.08) | 0.889 | 10.145 | <0.0001***** |
| Boys | -0.876 (-0.9 - -0.85) | 0.416 | 11.072 | <0.0001***** | .. | .. | .. | .. | .. | .. | .. | .. |
| Non-binary | 0.036 (-0.04 - 0.12) | 1.037 | 14.430 | 0.373 | .. | .. | .. | .. | .. | .. | .. | .. |
| Parental social support | -0.075 (-0.08 - -0.07) | 0.928 | 14.036 | <0.0001***** | -0.088 (-0.09 - -0.08) | 0.916 | 14.014 | <0.0001***** | -0.060 (-0.06 - -0.06) | 0.942 | 10.340 | <0.0001***** |
| Language spoken at home | -0.030 (-0.06 - 0.00) | 0.971 | 14.197 | 0.023 | -0.091 (-0.13 - -0.05) | 0.913 | 14.003 | <0.0001***** | .. | .. | .. | .. |
| Watching videos | 0.058 (0.05 - 0.06) | 1.059 | 14.505 | <0.0001***** | 0.063 (0.05 - 0.07) | 1.065 | 14.545 | <0.0001***** | 0.054 (0.05 - 0.06) | 1.056 | 10.740 | <0.0001***** |
| Gaming alone | 0.007 (0.00 - 0.01) | 1.007 | 14.327 | 0.089 | .. | .. |  | .. | .. | .. |  | .. |
| Gaming with friends | .. | .. | .. | .. | .. | .. |  | .. | 0.016 (0.01 - 0.02) | 1.016 | 10.603 | 0.0002***** |
| Social media | 0.053 (0.05 - 0.06) | 1.054 | 14.489 | <0.0001***** | 0.068 (0.06 - 0.08) | 1.071 | 14.565 | <0.0001***** | 0.030 (0.02 - 0.04) | 1.031 | 10.654 | <0.0001***** |
| Other screen usage | 0.051 (0.04 - 0.06) | 1.052 | 14.482 | <0.0001***** | 0.068 (0.05 - 0.08) | 1.070 | 14.564 | <0.0001***** | 0.026 (0.01 - 0.04) | 1.026 | 10.639 | <0.0001***** |
| Sickness | 0.405 (0.36 - 0.45) | 1.500 | 15.651 | <0.0001***** | 0.422 (0.35 - 0.49) | 1.524 | 15.721 | <0.0001***** | 0.348 (0.28 - 0.41) | 1.416 | 11.809 | <0.0001***** |
| Bad grade | 0.273 (0.25 - 0.30) | 1.314 | 15.233 | <0.0001***** | 0.301 (0.26 - 0.34) | 1.352 | 15.345 | <0.0001***** | 0.231 (0.19 - 0.27) | 1.259 | 11.376 | <0.0001***** |
| Breakup | 0.227 (0.19 - 0.26) | 1.255 | 15.081 | <0.0001***** | 0.241 (0.19 - 0.29) | 1.273 | 15.150 | <0.0001***** | 0.203 (0.15 - 0.25) | 1.225 | 11.274 | <0.0001***** |
| Argument with parents | 0.301 (0.26 - 0.34) | 1.351 | 15.322 | <0.0001***** | 0.175 (0.12 - 0.23) | 1.191 | 14.929 | <0.0001***** | 0.355 (0.30 - 0.41) | 1.426 | 11.835 | <0.0001***** |
| Argument among parents | .. | .. | .. | .. | 0.234 (0.17 - 0.30) | 1.263 | 15.124 | <0.0001***** | .. | .. | .. | .. |
| Death of a friend | 0.228 (0.16 - 0.29) | 1.256 | 15.085 | <0.0001***** | 0.184 (0.09 - 0.27) | 1.202 | 14.958 | <0.0001***** | 0.347 (0.25 - 0.44) | 1.416 | 11.807 | <0.0001***** |
| Peer sexual abuse | 0.518 (0.44 - 0.59) | 1.679 | 15.992 | <0.0001***** | 0.485 (0.40 - 0.57) | 1.624 | 15.913 | <0.0001***** | .. | .. | .. | .. |
| Accident | 0.247 (0.20 - 0.29) | 1.280 | 15.146 | <0.0001***** | 0.253 (0.18 - 0.32) | 1.288 | 15.189 | <0.0001***** | 0.278 (0.22 - 0.33) | 1.320 | 11.548 | <0.0001***** |
|  | **Hostility (score range 5-20)** | | | | | | | | | | | |
|  | **All genders** | | | | **Girls** | | | | **Boys** | | | |
| **Predictors** | **β (CI)** | **Odds ratio** | **Survey score equivalent** | **p value** | **β (CI)** | **Odds ratio** | **Survey score equivalent** | **p value** | **β (CI)** | **Odds ratio** | **Survey score equivalent** | **p value** |
| 2018 | .. | .. | .. | .. | 0.059 (0.01 - 0.10) | 1.061 | 11.978 | 0.012 | 0.226 (0.15 - 0.30) | 1.254 | 10.553 | <0.0001***** |
| 2020 | .. | .. | .. | .. | 0.255 (0.21 - 0.30) | 1.291 | 12.713 | <0.0001***** | 0.066 (-0.02 - 0.15) | 1.068 | 10.005 | 0.113 |
| 2022 | .. | .. | .. | .. | 0.193 (0.14 - 0.24) | 1.213 | 12.481 | <0.0001***** | 0.033 (-0.05 - 0.11) | 1.033 | 9.895 | 0.414 |
| 2023 | .. | .. | .. | .. | 0.219 (0.17 - 0.27) | 1.244 | 12.577 | <0.0001***** | 0.097 (0.02 - 0.18) | 1.102 | 10.110 | 0.017 |
| 14-years-old | .. | .. | .. | .. | .. | .. | .. | .. | -0.004 (-0.08 - 0.07) | 0.996 | 9.775 | 0.920 |
| 15-years-old | .. | .. | .. | .. | .. | .. | .. | .. | 0.042 (-0.03 - 0.12) | 1.042 | 9.924 | 0.283 |
| Boys | -0.216 (-0.24 - -0.19) | 0.806 | 10.952 | <0.0001***** | .. | .. | .. | .. | -0.059 (-0.06 - -0.05) | 0.943 | 9.597 | <0.0001***** |
| Non-binary | 0.135 (0.05 - 0.22) | 1.144 | 12.246 | 0.002 | .. | .. | .. | .. | .. | .. | .. | .. |
| Parental social support | -0.079 (-0.08 - -0.08) | 0.924 | 11.452 | <0.0001***** | -0.093 (-0.1 - -0.09) | 0.911 | 11.415 | <0.0001***** | 0.037 (0.03 - 0.05) | 1.038 | 9.910 | <0.0001***** |
| Language spoken at home | -0.051 (-0.08 - -0.02) | 0.950 | 11.555 | <0.0001***** | -0.081 (-0.12 - -0.04) | 0.922 | 11.460 | <0.0001***** | 0.016 (0.01 - 0.03) | 1.017 | 9.841 | 0.001 |
| Watching videos | 0.050 (0.04 - 0.06) | 1.051 | 11.929 | <0.0001***** | 0.060 (0.05 - 0.07) | 1.062 | 11.984 | <0.0001***** | 0.077 (0.07 - 0.09) | 1.080 | 10.043 | <0.0001***** |
| Gaming alone | 0.020 (0.01 - 0.03) | 1.020 | 11.816 | <0.0001***** | 0.028 (0.01 - 0.05) | 1.029 | 11.865 | 0.001 | .. | .. | .. | .. |
| Gaming with friends | .. | .. | .. | .. | 0.021 (0.00 - 0.04) | 1.021 | 11.836 | 0.013 | 0.010 (0.00 - 0.02) | 1.010 | 9.819 | 0.032 |
| Social media | 0.095 (0.09 - 0.10) | 1.100 | 12.098 | <0.0001***** | 0.108 (0.10 - 0.12) | 1.114 | 12.162 | <0.0001***** | .. | .. | .. | .. |
| Sickness | 0.088 (0.04 - 0.13) | 1.091 | 12.069 | 0.0003***** | 0.154 (0.08 - 0.22) | 1.167 | 12.336 | <0.0001***** | -0.282 (-0.48 - -0.08) | 0.754 | 8.919 | 0.006 |
| Bad grade | 0.287 (0.26 - 0.31) | 1.333 | 12.817 | <0.0001***** | 0.302 (0.26 - 0.34) | 1.353 | 12.890 | <0.0001***** | 0.276 (0.24 - 0.31) | 1.318 | 10.727 | <0.0001***** |
| Breakup | 0.380 (0.34 - 0.42) | 1.462 | 13.164 | <0.0001***** | 0.380 (0.33 - 0.43) | 1.462 | 13.179 | <0.0001***** | 0.339 (0.29 - 0.39) | 1.404 | 10.953 | <0.0001***** |
| Argument with parents | 0.818 (0.78 - 0.86) | 2.266 | 14.737 | <0.0001***** | 0.898 (0.78 - 1.02) | 2.454 | 15.021 | <0.0001***** | 0.828 (0.77 - 0.89) | 2.290 | 12.766 | <0.0001***** |
| Argument among parents | 0.184 (0.13 - 0.24) | 1.202 | 12.429 | <0.0001***** | 0.178 (0.11 - 0.25) | 1.195 | 12.423 | <0.0001***** | -0.084 (-0.27 - 0.11) | 0.920 | 9.519 | 0.390 |
| Parental unemployment | 0.215 (0.16 - 0.27) | 1.240 | 32.326 | <0.0001***** | 0.129 (0.06 - 0.20) | 1.138 | 30.854 | 0.0003***** | 0.267 (0.19 - 0.34) | 1.306 | 23.410 | <0.0001***** |
| Parental divorce | .. | .. | .. | .. | 0.110 (0.01 - 0.21) | 1.116 | 12.168 | 0.034 | 0.040 (-0.08 - 0.15) | 1.038 | 9.908 | 0.520 |
| Witness domestic violence | .. | .. | .. | .. | .. | .. | .. | .. | 0.115 (-0.07 - 0.30) | 1.122 | 10.169 | 0.217 |
| Subject of domestic violence | 0.166 (0.06 - 0.27) | 1.180 | 12.362 | 0.002 | 0.156 (0.02 - 0.29) | 1.168 | 12.340 | 0.028 | 0.206 (0.01 - 0.40) | 1.228 | 10.481 | 0.041 |
| Parental death | .. | .. | .. | .. | .. | .. | .. | .. | -0.046 (-0.23 - 0.13) | 0.955 | 9.637 | 0.613 |
| Death of a friend | 0.257 (0.19 - 0.32) | 1.293 | 12.703 | <0.0001***** | 0.330 (0.14 - 0.52) | 1.391 | 12.993 | 0.001 | 0.270 (0.03 - 0.51) | 1.310 | 10.706 | 0.025 |
| Adult sexual abuse | .. | .. | .. | .. | 0.237 (0.10 - 0.38) | 1.268 | 12.646 | 0.001 | 0.308 (-0.26 - 0.88) | 1.361 | 10.841 | 0.291 |
| Peer sexual abuse | 0.464 (0.39 - 0.54) | 1.591 | 13.476 | <0.0001***** | 0.476 (0.39 - 0.57) | 1.610 | 13.536 | <0.0001***** | .. | .. | .. | .. |
| Accident | .. | .. | .. | .. | 0.255 (0.18 - 0.33) | 1.291 | 12.714 | <0.0001***** | 0.258 (0.12 - 0.40) | 1.294 | 10.663 | <0.0003***** |
| 2018*Friend death | .. | .. | .. | .. | 0.034 (-0.23 - 0.30) | 1.034 | 11.885 | 0.802 | 0.126 (-0.18 - 0.44) | 1.134 | 10.206 | 0.426 |
| 2020*Friend death | .. | .. | .. | .. | -0.204 (-0.48 - 0.07) | 0.815 | 11.011 | 0.149 | 0.109 (-0.23 - 0.44) | 1.115 | 10.148 | 0.524 |
| 2022*Friend death | .. | .. | .. | .. | -0.391 (-0.66 - -0.12) | 0.676 | 10.352 | 0.004 | -0.036 (-0.36 - 0.29) | 0.964 | 9.670 | 0.828 |
| 2023*Friend death | .. | .. | .. | .. | -0.072 (-0.37 - 0.23) | 0.931 | 11.493 | 0.638 | -0.096 (-0.42 - 0.23) | 0.909 | 9.481 | 0.567 |
| 2018*Argument with parents | .. | .. | .. | .. | -0.196 (-0.37 - -0.02) | 0.822 | 11.039 | 0.026 | .. | .. | .. | .. |
| 2020*Argument with parents | .. | .. | .. | .. | -0.173 (-0.33 - -0.02) | 0.841 | 11.123 | 0.028 | .. | .. | .. | .. |
| 2022*Argument with parents | .. | .. | .. | .. | -0.010 (-0.25 - 0.06) | 0.905 | 11.391 | 0.208 | .. | .. | .. | .. |
| 2023*Argument with parents | .. | .. | .. | .. | -0.145 (-0.30 - 0.01) | 0.865 | 11.225 | 0.067 | .. | .. | .. | .. |
| 14-years-old*2018 | .. | .. | .. | .. | .. | .. | .. | .. | -0.105 (-0.21 - 0.00) | 0.900 | 9.452 | 0.055 |
| 15-years-old*2018 | .. | .. | .. | .. | .. | .. | .. | .. | -0.132 (-0.24 - -0.02) | 0.876 | 9.367 | 0.017 |
| 14-years-old*2020 | .. | .. | .. | .. | .. | .. | .. | .. | -0.045 (-0.16 - 0.07) | 0.956 | 9.643 | 0.430 |
| 15-years-old*2020 | .. | .. | .. | .. | .. | .. | .. | .. | 0.001 (-0.11 - 0.11) | 1.001 | 9.792 | 0.981 |
| 14 years-old*2022 | .. | .. | .. | .. | .. | .. | .. | .. | -0.099 (-0.21 - 0.01) | 0.905 | 9.469 | 0.069 |
| 15-years-old*2022 | .. | .. | .. | .. | .. | .. | .. | .. | -0.215 (-0.32 - -0.11) | 0.807 | 9.116 | 0.0001***** |
| 14-years-old*2023 | .. | .. | .. | .. | .. | .. | .. | .. | -0.065 (-0.18 - 0.04) | 0.937 | 9.576 | 0.242 |
| 15-years-old*2023 | .. | .. | .. | .. | .. | .. | .. | .. | -0.194 (-0.3 - -0.08) | 0.824 | 9.178 | 0.001 |
| Argument with parents*2018 | .. | .. | .. | .. | .. | .. | .. | .. | 0.155 (-0.12 - 0.43) | 1.167 | 10.305 | 0.277 |
| Argument with parents*2020 | .. | .. | .. | .. | .. | .. | .. | .. | 0.399 (0.14 - 0.66) | 1.491 | 11.170 | 0.003 |
| Argument with parents*2022 | .. | .. | .. | .. | .. | .. | .. | .. | 0.351 (0.09 - 0.61) | 1.421 | 10.997 | 0.007 |
| Argument with parents*2023 | .. | .. | .. | .. | .. | .. | .. | .. | 0.291 (0.03 - 0.56) | 1.338 | 10.782 | 0.031 |
| Accident*2018 | .. | .. | .. | .. | .. | .. | .. | .. | -0.232 (-0.42 - -0.05) | 0.793 | 9.065 | 0.015 |
| Accident*2020 | .. | .. | .. | .. | .. | .. | .. | .. | 0.024 (-0.17 - 0.21) | 1.024 | 9.865 | 0.805 |
| Accident*2022 | .. | .. | .. | .. | .. | .. | .. | .. | 0.003 (-0.18 - 0.19) | 1.003 | 9.797 | 0.976 |
| Accident*2023 | .. | .. | .. | .. | .. | .. | .. | .. | 0.095 (-0.09 - 0.28) | 1.100 | 10.103 | 0.316 |
| 2018*Sickness | .. | .. | .. | .. | .. | .. | .. | .. | 0.162 (-0.10 - 0.42) | 1.175 | 10.329 | 0.222 |
| 2020*Sickness | .. | .. | .. | .. | .. | .. | .. | .. | 0.172 (-0.11 - 0.45) | 1.188 | 10.365 | 0.225 |
| 2022*Sickness | .. | .. | .. | .. | .. | .. | .. | .. | 0.425 (0.20 - 0.65) | 1.529 | 11.263 | 0.0002***** |
| 2023*Sickness | .. | .. | .. | .. | .. | .. | .. | .. | 0.378 (0.13 - 0.63) | 1.459 | 11.092 | 0.003 |
| Parental unemployment*2018 | .. | .. | .. | .. | .. | .. | .. | .. | -0.205 (-0.48 - 0.07) | 0.815 | 9.145 | 0.138 |
| Parental unemployment*2020 | .. | .. | .. | .. | .. | .. | .. | .. | -0.151 (-0.38 - 0.08) | 0.860 | 9.310 | 0.195 |
| Parental unemployment*2022 | .. | .. | .. | .. | .. | .. | .. | .. | 0.007 (-0.26 - 0.27) | 1.007 | 9.809 | 0.961 |
| Parental unemployment*2023 | .. | .. | .. | .. | .. | .. | .. | .. | 0.074 (-0.24 - 0.38) | 1.077 | 10.033 | 0.638 |
| 2018* Adult sexual abuse | .. | .. | .. | .. | .. | .. | .. | .. | -0.979 (-1.78 - -0.18) | 0.376 | 7.246 | 0.017 |
| 2020* Adult sexual abuse | .. | .. | .. | .. | .. | .. | .. | .. | -0.722 (-1.53 - 0.08) | 0.486 | 7.782 | 0.079 |
| 2022* Adult sexual abuse | .. | .. | .. | .. | .. | .. | .. | .. | -0.219 (-0.94 - 0.51) | 0.804 | 9.105 | 0.555 |
| 2023* Adult sexual abuse | .. | .. | .. | .. | .. | .. | .. | .. | -1.068 (-1.80 - -0.33) | 0.344 | 7.081 | 0.004 |

*Note:* *: Significant after Bonferroni correction (<0.00043). Missing variables (··) were dropped from models during stepwise model selection using Likelihood-Ratio Tests and BIC optimisation, indicating they did not significantly improve model fit. All starting formulas for the models were the same and included all independent variables and their interactions with survey year.

Table S12. Base model predictions for adolescent mental health outcomes (2016 Reference Year)**.** Fixed effects of survey year (indexed to 2016), age, gender and interactions on mental health outcomes (depressive symptom, anxiety symptom and hostility scores), results are presented for all gender models, and gender-stratified models for girls and boys separately. Model estimates from beta mixed-effects models (β) are presented with CI, log-odds ratios, alongside survey score equivalents to the cumulative survey scores, and significance value (*p*).

| Depressive symptoms (score range 10-40) | | | | | | | | | | | | |
| --- | --- | --- | --- | --- | --- | --- | --- | --- | --- | --- | --- | --- |
|  | All genders | | | | Girls | | | | Boys | | | |
| **Predictors** | **β (CI)** | **Odds ratio** | **Survey score equivalent** | **p value** | **β (CI)** | **Odds ratio** | **Survey score equivalent** | **p value** | **β (CI)** | **Odds ratio** | **Survey score equivalent** | **p value** |
| 2018 | 0.125 (0.07 – 0.18) | 1.133 | 18.759 | <0.0001***** | 0.091 (0.02 – 0.17) | 1.095 | 18.027 | 0.019 | 0.170 (0.10 – 0.24) | 1.185 | 15.468 | <0.0001***** |
| 2020 | 0.536 (0.48 – 0.59) | 1.709 | 21.501 | <0.0001***** | 0.568 (0.49 – 0.65) | 1.764 | 21.115 | <0.0001***** | 0.511 (0.43 – 0.59) | 1.667 | 17.160 | <0.0001***** |
| 2021 | 0.569 (0.52 – 0.62) | 1.766 | 21.736 | <0.0001***** | 0.624 (0.55 – 0.70) | 1.866 | 21.508 | <0.0001***** | 0.491 (0.42 – 0.56) | 1.634 | 17.053 | <0.0001***** |
| 2022 | 0.562 (0.51 – 0.62) | 1.754 | 21.687 | <0.0001***** | 0.571 (0.49 – 0.65) | 1.770 | 21.138 | <0.0001***** | 0.522 (0.45 – 0.60) | 1.685 | 17.221 | <0.0001***** |
| 2023 | 0.380 (0.33 – 0.44) | 1.463 | 20.420 | <0.0001***** | 0.390 (0.31 – 0.47) | 1.477 | 19.902 | <0.0001***** | 0.359 (0.28 – 0.44) | 1.432 | 16.367 | <0.0001***** |
| 14-years-old | 0.198 (0.14 – 0.25) | 1.219 | 19.216 | <0.0001***** | 0.339 (0.26 – 0.42) | 1.404 | 19.569 | <0.0001***** | 0.053 (-0.02 – 0.13) | 1.054 | 14.965 | 0.156 |
| 15-years-old | 0.286 (0.23 – 0.34) | 1.331 | 19.787 | <0.0001***** | 0.493 (0.42 – 0.57) | 1.638 | 20.599 | <0.0001***** | 0.087 (0.01 – 0.16) | 1.091 | 15.107 | 0.020 |
| Boys | -0.682 (-0.70 – -0.66) | 0.506 | 14.661 | <0.0001***** | .. | .. | .. | .. | .. | .. | .. | .. |
| Non-binary | 0.537 (0.47 – 0.61) | 1.711 | 21.510 | <0.0001***** | .. | .. | .. | .. | .. | .. | .. | .. |
| 2018*14-years-old | -0.018 (-0.09 – 0.06) | 0.982 | 17.897 | 0.638 | -0.105 (-0.21 – 0.00) | 0.900 | 16.929 | 0.058 | 0.061 (-0.04 – 0.16) | 1.063 | 14.998 | 0.248 |
| 2020*14-years-old | -0.163 (-0.24 – -0.08) | 0.849 | 17.081 | <0.0001***** | -0.160 (-0.27 – -0.05) | 0.853 | 16.643 | 0.006 | -0.140 (-0.25 – -0.03) | 0.869 | 14.216 | 0.011 |
| 2021*14-years-old | -0.180 (-0.25 – -0.11) | 0.835 | 16.993 | <0.0001***** | -0.188 (-0.30 – -0.08) | 0.829 | 16.498 | 0.001 | -0.120 (-0.22 – -0.02) | 0.887 | 14.290 | 0.020 |
| 2022*14-years-old | -0.227 (-0.30 – -0.15) | 0.797 | 16.743 | <0.0001***** | -0.204 (-0.32 – -0.09) | 0.816 | 16.416 | 0.0004* | -0.215 (-0.32 – -0.11) | 0.807 | 13.953 | <0.0001***** |
| 2023*14-years-old | -0.080 (-0.16 – -0.00) | 0.923 | 17.544 | 0.044 | -0.096 (-0.21 – 0.02) | 0.908 | 16.976 | 0.098 | -0.055 (-0.16 – 0.05) | 0.946 | 14.532 | 0.309 |
| 2018*15-years-old | -0.108 (-0.18 – -0.03) | 0.898 | 17.387 | 0.005 | -0.170 (-0.28 – -0.06) | 0.844 | 16.588 | 0.002 | -0.064 (-0.17 – 0.04) | 0.938 | 14.497 | 0.227 |
| 2020*15-years-old | -0.226 (-0.31 – -0.15) | 0.798 | 16.750 | <0.0001***** | -0.333 (-0.45 – -0.22) | 0.716 | 15.787 | <0.0001***** | -0.109 (-0.22 – -0.00) | 0.896 | 14.328 | 0.050 |
| 2021*15-years-old | -0.212 (-0.29 – -0.14) | 0.809 | 16.820 | <0.0001***** | -0.326 (-0.43 – -0.22) | 0.722 | 15.823 | <0.0001***** | -0.067 (-0.17 – 0.03) | 0.935 | 14.486 | 0.196 |
| 2022*15-years-old | -0.262 (-0.34 – -0.19) | 0.770 | 16.563 | <0.0001***** | -0.211 (-0.32 – -0.10) | 0.810 | 16.381 | 0.0003***** | -0.272 (-0.38 – -0.17) | 0.762 | 13.761 | <0.0001***** |
| 2023*15-years-old | -0.219 (-0.30 – -0.14) | 0.804 | 16.787 | <0.0001***** | -0.257 (-0.37 – -0.14) | 0.773 | 16.151 | <0.0001***** | -0.159 (-0.27 – -0.05) | 0.853 | 14.148 | 0.004 |
| Anxiety symptoms (score range 5-20) | | | | | | | | | | | | |
|  | All genders | | | | Girls | | | | Boys | | | |
| **Predictors** | **β (CI)** | **Odds ratio** | **Survey score equivalent** | **p value** | **β (CI)** | **Odds ratio** | **Survey score equivalent** | **p value** | **β (CI)** | **Odds ratio** | **Survey score equivalent** | **p value** |
| 2018 | 0.090 (0.03 – 0.14) | 1.094 | 10.927 | 0.001 | 0.053 (-0.02 – 0.13) | 1.055 | 10.318 | 0.179 | 0.131 (0.06 – 0.21) | 1.140 | 8.426 | 0.001 |
| 2020 | 0.224 (0.17 – 0.28) | 1.251 | 11.415 | <0.0001***** | 0.226 (0.14 – 0.31) | 1.254 | 10.926 | <0.0001***** | 0.231 (0.15 – 0.31) | 1.259 | 8.696 | <0.0001***** |
| 2021 | 0.212 (0.16 – 0.27) | 1.236 | 11.368 | <0.0001***** | 0.287 (0.21 – 0.36) | 1.333 | 11.145 | <0.0001***** | 0.121 (0.05 – 0.19) | 1.129 | 8.400 | 0.001 |
| 2022 | 0.388 (0.33 – 0.44) | 1.475 | 12.024 | <0.0001***** | 0.472 (0.39 – 0.55) | 1.603 | 11.823 | <0.0001***** | 0.289 (0.21 – 0.36) | 1.335 | 8.860 | <0.0001***** |
| 2023 | 0.295 (0.24 – 0.35) | 1.342 | 11.674 | <0.0001***** | 0.340 (0.26 – 0.42) | 1.405 | 11.337 | <0.0001***** | 0.249 (0.17 – 0.33) | 1.282 | 8.747 | <0.0001***** |
| 14-years-old | 0.184 (0.13 – 0.24) | 1.202 | 11.267 | <0.0001***** | 0.289 (0.21 – 0.37) | 1.335 | 11.150 | <0.0001***** | 0.079 (0.00 – 0.15) | 1.082 | 8.289 | 0.041 |
| 15-years-old | 0.215 (0.16 – 0.27) | 1.240 | 11.383 | <0.0001***** | 0.411 (0.33 – 0.49) | 1.508 | 11.598 | <0.0001***** | 0.030 (-0.04 – 0.11) | 1.031 | 8.167 | 0.427 |
| Boys | -0.919 (-0.94 – -0.90) | 0.399 | 7.885 | <0.0001***** | .. | .. | .. | .. | .. | .. | .. | .. |
| Non-binary | 0.234 (0.16 – 0.31) | 1.263 | 11.450 | <0.0001***** | .. | .. | .. | .. | .. | .. | .. | .. |
| 2018*14-years-old | -0.079 (-0.16 – -0.00) | 0.924 | 10.333 | 0.045 | -0.078 (-0.19 – 0.03) | 0.925 | 9.877 | 0.171 | -0.087 (-0.19 – 0.02) | 0.916 | 7.883 | 0.108 |
| 2020*14-years-old | -0.143 (-0.22 – -0.06) | 0.867 | 10.117 | 0.001 | -0.041 (-0.16 – 0.08) | 0.959 | 9.997 | 0.486 | -0.216 (-0.33 – -0.11) | 0.806 | 7.596 | 0.0001***** |
| 2021*14-years-old | -0.149 (-0.22 – -0.07) | 0.862 | 10.097 | 0.0001***** | -0.097 (-0.21 – 0.01) | 0.908 | 9.813 | 0.084 | -0.155 (-0.26 – -0.05) | 0.857 | 7.729 | 0.003 |
| 2022*14-years-old | -0.230 (-0.31 – -0.15) | 0.795 | 9.828 | <0.0001***** | -0.137 (-0.25 – -0.02) | 0.872 | 9.685 | 0.02 | -0.290 (-0.40 – -0.18) | 0.748 | 7.440 | <0.0001***** |
| 2023*14-years-old | -0.140 (-0.22 – -0.06) | 0.870 | 10.127 | 0.001 | -0.111 (-0.23 – 0.01) | 0.895 | 9.767 | 0.061 | -0.154 (-0.26 – -0.04) | 0.858 | 7.731 | 0.006 |
| 2018*15-years-old | -0.128 (-0.21 – -0.05) | 0.880 | 10.166 | 0.001 | -0.192 (-0.30 – -0.08) | 0.825 | 9.508 | 0.001 | -0.076 (-0.18 – 0.03) | 0.927 | 7.909 | 0.164 |
| 2020*15-years-old | -0.182 (-0.26 – -0.10) | 0.834 | 9.986 | <0.0001***** | -0.205 (-0.32 – -0.09) | 0.815 | 9.468 | 0.001 | -0.144 (-0.26 – -0.03) | 0.866 | 7.754 | 0.012 |
| 2021*15-years-old | -0.155 (-0.23 – -0.08) | 0.856 | 10.075 | <0.0001* | -0.266 (-0.38 – -0.16) | 0.766 | 9.278 | <0.0001***** | -0.033 (-0.14 – 0.07) | 0.967 | 8.011 | 0.532 |
| 2022*15-years-old | -0.230 (-0.31 – -0.15) | 0.795 | 9.828 | <0.0001* | -0.204 (-0.32 – -0.09) | 0.816 | 9.471 | 0.001 | -0.230 (-0.34 – -0.12) | 0.794 | 7.565 | <0.0001***** |
| 2023*15-years-old | -0.202 (-0.28 – -0.12) | 0.817 | 9.919 | <0.0001* | -0.182 (-0.30 – -0.07) | 0.833 | 9.539 | 0.002 | -0.205 (-0.31 – -0.09) | 0.815 | 7.619 | 0.0003***** |
| Hostility (score range 5-20) | | | | | | | | | | | | |
|  | All genders | | | | Girls | | | | Boys | | | |
| **Predictors** | **β (CI)** | **Odds ratio** | **Survey score equivalent** | **p value** | **β (CI)** | **Odds ratio** | **Survey score equivalent** | **p value** | **β (CI)** | **Odds ratio** | **Survey score equivalent** | **p value** |
| 2018 | 0.133 (0.08 – 0.19) | 1.142 | 9.105 | <0.0001***** | 0.075 (-0.00 – 0.15) | 1.078 | 8.627 | 0.057 | 0.209 (0.13 – 0.29) | 1.232 | 8.666 | <0.0001***** |
| 2020 | 0.287 (0.23 – 0.34) | 1.332 | 9.581 | <0.0001***** | 0.412 (0.33 – 0.50) | 1.510 | 9.632 | <0.0001***** | 0.153 (0.07 – 0.23) | 1.165 | 8.515 | 0.0002***** |
| 2021 | 0.328 (0.27 – 0.38) | 1.388 | 9.712 | <0.0001***** | 0.475 (0.40 – 0.55) | 1.608 | 9.835 | <0.0001***** | 0.157 (0.08 – 0.23) | 1.170 | 8.525 | <0.0001***** |
| 2022 | 0.291 (0.24 – 0.35) | 1.338 | 9.594 | <0.0001***** | 0.405 (0.32 – 0.49) | 1.499 | 9.608 | <0.0001***** | 0.159 (0.08 – 0.24) | 1.172 | 8.530 | <0.0001***** |
| 2023 | 0.256 (0.20 – 0.31) | 1.292 | 9.483 | <0.0001***** | 0.343 (0.26 – 0.42) | 1.409 | 9.413 | <0.0001***** | 0.153 (0.07 – 0.23) | 1.165 | 8.514 | 0.0002***** |
| 14-years-old | 0.108 (0.05 – 0.16) | 1.114 | 9.033 | 0.0002***** | 0.178 (0.10 – 0.26) | 1.195 | 8.917 | <0.0001***** | 0.031 (-0.05 – 0.11) | 1.032 | 8.198 | 0.426 |
| 15-years-old | 0.170 (0.11 – 0.23) | 1.185 | 9.218 | <0.0001***** | 0.257 (0.18 – 0.34) | 1.294 | 9.151 | <0.0001***** | 0.080 (0.00 – 0.16) | 1.083 | 8.322 | 0.042 |
| Boys | -0.317 (-0.34 – -0.30) | 0.728 | 7.907 | <0.0001***** | .. | .. | .. | .. | .. | .. | .. | .. |
| Non-binary | 0.388 (0.31 – 0.46) | 1.474 | 9.908 | <0.0001***** | .. | .. | .. | .. | .. | .. | .. | .. |
| 2018*14-years-old | -0.046 (-0.12 – 0.03) | 0.955 | 8.594 | 0.244 | -0.030 (-0.14 – 0.08) | 0.971 | 8.346 | 0.599 | -0.078 (-0.19 – 0.03) | 0.925 | 7.931 | 0.160 |
| 2020*14-years-old | -0.103 (-0.18 – -0.02) | 0.902 | 8.441 | 0.013 | -0.099 (-0.22 – 0.02) | 0.906 | 8.170 | 0.099 | -0.092 (-0.20 – 0.02) | 0.912 | 7.899 | 0.110 |
| 2021*14-years-old | -0.174 (-0.25 – -0.10) | 0.840 | 8.255 | <0.0001***** | -0.147 (-0.26 – -0.04) | 0.863 | 8.052 | 0.009 | -0.164 (-0.27 – -0.06) | 0.849 | 7.735 | 0.002 |
| 2022*14-years-old | -0.167 (-0.25 – -0.09) | 0.847 | 8.275 | <0.0001***** | -0.120 (-0.24 – -0.00) | 0.887 | 8.118 | 0.042 | -0.167 (-0.28 – -0.06) | 0.846 | 7.726 | 0.003 |
| 2023*14-years-old | -0.029 (-0.11 – 0.05) | 0.971 | 8.641 | 0.480 | 0.025 (-0.09 – 0.14) | 1.026 | 8.492 | 0.670 | -0.062 (-0.17 – 0.05) | 0.940 | 7.968 | 0.274 |
| 2018*15-years-old | -0.075 (-0.15 – 0.00) | 0.928 | 8.517 | 0.063 | -0.069 (-0.18 – 0.04) | 0.934 | 8.246 | 0.228 | -0.099 (-0.21 – 0.01) | 0.905 | 7.881 | 0.077 |
| 2020*15-years-old | -0.100 (-0.18 – -0.02) | 0.905 | 8.449 | 0.017 | -0.168 (-0.29 – -0.05) | 0.846 | 8.001 | 0.006 | -0.016 (-0.13 – 0.10) | 0.985 | 8.081 | 0.790 |
| 2021*15-years-old | -0.215 (-0.29 – -0.14) | 0.806 | 8.153 | <0.0001***** | -0.275 (-0.39 – -0.16) | 0.759 | 7.752 | <0.0001***** | -0.135 (-0.24 – -0.03) | 0.874 | 7.800 | 0.013 |
| 2022*15-years-old | -0.233 (-0.31 – -0.15) | 0.792 | 8.109 | <0.0001***** | -0.162 (-0.28 – -0.05) | 0.851 | 8.016 | 0.006 | -0.263 (-0.37 – -0.15) | 0.769 | 7.520 | <0.0001***** |
| 2023*15-years-old | -0.152 (-0.23 – -0.07) | 0.859 | 8.314 | 0.0002***** | -0.092 (-0.21 – 0.02) | 0.912 | 8.187 | 0.122 | -0.180 (-0.29 – -0.07) | 0.835 | 7.698 | 0.002 |

*Note:* *: Significant after Bonferroni correction (<0.00043).

Table S13. Model predictions for proportions of adolescents with high mental health scores (2016 Reference Year). Fixed effects of time, age and interactions on the proportion of individuals with high depression, anxiety and hostility scores (scores equal to or above those of the 5th centile determined separately for boys and girls for each age group in 2016). Model estimates presented from logistic mixed effects models (odd ratio, OR) with CI and statistical significance (*p*). Models are presented separately for girls and boys.

| **High-threshold depressive symptom scores** | **Girls** |  |  | **Boys** | |  |
| --- | --- | --- | --- | --- | --- | --- |
|  | **OR** | **CI** | **p value** | **OR** | **CI** | **p value** |
| 2018 | 1.064 | 0.777 - 1.457 | 0.698 | 1.653 | 1.229 - 2.223 | 0.001 |
| 2020 | 1.650 | 1.219 - 2.233 | 0.001 | 1.373 | 1.000 - 1.886 | 0.050 |
| 2021 | 2.140 | 1.621 - 2.824 | <0.0001* | 1.558 | 1.162 - 2.087 | 0.003 |
| 2022 | 1.984 | 1.487 - 2.646 | <0.0001* | 1.938 | 1.451 - 2.589 | <0.0001* |
| 2023 | 1.485 | 1.091 - 2.021 | 0.012 | 1.567 | 1.154 - 2.127 | 0.004 |
| 14-years-old | 0.951 | 0.687 - 1.315 | 0.760 | 1.165 | 0.849 - 1.600 | 0.344 |
| 15-years-old | 0.961 | 0.697 - 1.327 | 0.811 | 1.157 | 0.843 - 1.588 | 0.367 |
| 2018*14-years-old | 0.711 | 0.444 - 1.139 | 0.156 | 0.746 | 0.492 - 1.130 | 0.167 |
| 2020*14-years-old | 0.819 | 0.528 - 1.272 | 0.374 | 1.045 | 0.681 - 1.604 | 0.840 |
| 2021*14-years-old | 0.682 | 0.454 - 1.026 | 0.067 | 0.906 | 0.605 - 1.355 | 0.629 |
| 2022*14-years-old | 0.618 | 0.401 - 0.952 | 0.029 | 0.865 | 0.579 - 1.291 | 0.478 |
| 2023*14-years-old | 0.813 | 0.518 - 1.276 | 0.368 | 0.742 | 0.483 - 1.140 | 0.173 |
| 2018*15-years-old | 0.704 | 0.439 - 1.130 | 0.146 | 0.792 | 0.522 - 1.202 | 0.274 |
| 2020*15-years-old | 0.518 | 0.322 - 0.833 | 0.007 | 0.920 | 0.593 - 1.427 | 0.711 |
| 2021*15-years-old | 0.392 | 0.253 - 0.607 | <0.0001* | 0.971 | 0.649 - 1.453 | 0.886 |
| 2022*15-years-old | 0.522 | 0.336 - 0.813 | 0.004 | 0.813 | 0.541 - 1.219 | 0.316 |
| 2023*15-years-old | 0.542 | 0.336 - 0.874 | 0.012 | 0.734 | 0.476 - 1.133 | 0.163 |
| **High threshold anxiety symptom scores** | **Girls** |  |  | **Boys** | |  |
|  | **OR** | **CI** | **p value** | **OR** | **CI** | **p value** |
| 2016 | 0.955 | 0.698 - 1.305 | 0.771 | 0.862 | 0.626 - 1.187 | 0.363 |
| 2018 | 1.141 | 0.832 - 1.566 | 0.413 | 1.317 | 0.972 - 1.785 | 0.076 |
| 2020 | 1.360 | 1.019 - 1.817 | 0.037 | 0.967 | 0.714 - 1.309 | 0.826 |
| 2021 | 1.846 | 1.390 - 2.450 | <0.0001* | 1.604 | 1.209 - 2.129 | 0.001 |
| 2022 | 1.250 | 0.920 - 1.699 | 0.154 | 1.511 | 1.128 - 2.024 | 0.006 |
| 14-years-old | 0.583 | 0.407 - 0.835 | 0.003 | 1.020 | 0.749 - 1.389 | 0.901 |
| 15-years-old | 0.608 | 0.427 - 0.865 | 0.006 | 0.844 | 0.611 - 1.164 | 0.301 |
| 2016*14-years-old | 0.692 | 0.401 - 1.194 | 0.186 | 0.936 | 0.594 - 1.474 | 0.775 |
| 2018*14-years-old | 0.826 | 0.487 - 1.400 | 0.477 | 0.750 | 0.485 - 1.162 | 0.198 |
| 2020*14-years-old | 0.833 | 0.515 - 1.347 | 0.456 | 1.144 | 0.751 - 1.741 | 0.531 |
| 2021*14-years-old | 0.897 | 0.563 - 1.429 | 0.648 | 0.923 | 0.618 - 1.378 | 0.694 |
| 2022*14-years-old | 0.637 | 0.370 - 1.095 | 0.103 | 0.874 | 0.577 - 1.324 | 0.525 |
| 2016*15-years-old | 0.527 | 0.297 - 0.936 | 0.029 | 0.838 | 0.514 - 1.366 | 0.478 |
| 2018*15-years-old | 0.571 | 0.322 - 1.011 | 0.055 | 0.688 | 0.430 - 1.102 | 0.120 |
| 2020*15-years-old | 0.561 | 0.338 - 0.933 | 0.026 | 0.926 | 0.589 - 1.458 | 0.741 |
| 2021*15-years-old | 0.644 | 0.397 - 1.043 | 0.074 | 0.601 | 0.385 - 0.940 | 0.026 |
| 2022*15-years-old | 0.740 | 0.440 - 1.246 | 0.257 | 0.642 | 0.407 - 1.014 | 0.057 |
|  | **Girls** | |  | **Boys** |  |  |
| **High threshold hostility scores** | **OR** | **CI** | **p value** | **OR** | **CI** | **p value** |
| 2016 | 1.140 | 0.845 - 1.540 | 0.392 | 1.293 | 0.928 - 1.802 | 0.129 |
| 2018 | 2.264 | 1.714 - 2.992 | <0.0001* | 1.281 | 0.906 - 1.812 | 0.162 |
| 2020 | 2.328 | 1.784 - 3.038 | <0.0001* | 1.424 | 1.036 - 1.957 | 0.029 |
| 2021 | 1.911 | 1.443 - 2.531 | <0.0001* | 1.595 | 1.158 - 2.197 | 0.004 |
| 2022 | 1.781 | 1.336 - 2.373 | <0.0001* | 1.781 | 1.291 - 2.458 | 0.00044 |
| 14-years-old | 1.026 | 0.753 - 1.398 | 0.871 | 1.048 | 0.740 - 1.483 | 0.793 |
| 15-years-old | 0.974 | 0.713 - 1.330 | 0.869 | 1.029 | 0.726 - 1.457 | 0.874 |
| 2016*14-years-old | 0.658 | 0.421 - 1.028 | 0.066 | 0.976 | 0.613 - 1.553 | 0.918 |
| 2018*14-years-old | 0.737 | 0.494 - 1.100 | 0.136 | 0.807 | 0.494 - 1.319 | 0.393 |
| 2020*14-years-old | 0.636 | 0.433 - 0.937 | 0.022 | 0.707 | 0.446 - 1.119 | 0.139 |
| 2021*14-years-old | 0.783 | 0.523 - 1.173 | 0.235 | 0.948 | 0.605 - 1.487 | 0.817 |
| 2022*14-years-old | 1.048 | 0.701 - 1.567 | 0.819 | 0.689 | 0.434 - 1.096 | 0.116 |
| 2016*15-years-old | 0.771 | 0.495 - 1.202 | 0.251 | 0.931 | 0.581 - 1.493 | 0.768 |
| 2018*15-years-old | 0.720 | 0.478 - 1.085 | 0.117 | 1.057 | 0.652 - 1.716 | 0.821 |
| 2020*15-years-old | 0.442 | 0.293 - 0.665 | <0.0001* | 0.804 | 0.508 - 1.271 | 0.350 |
| 2021*15-years-old | 0.805 | 0.535 - 1.213 | 0.300 | 0.553 | 0.340 - 0.900 | 0.017 |
| 2022*15-years-old | 0.851 | 0.562 - 1.289 | 0.446 | 0.744 | 0.467 - 1.184 | 0.212 |

*Note*: *: Significant after Bonferroni correction (<0.00043).

Table S14: Spearman rank correlation matrix showing the relationship between the mental health symptom cumulative scores. Correlations from the all genders dataset are presented as well as gender-stratified analysis for girls and boys.

|  |  | Depressive symptom scores | Anxiety symptom scores | Hostility scores |
| --- | --- | --- | --- | --- |
| All genders | **Depressive symptom scores** | 1 | 0.7063927 | 0.5716755 |
|  | **Anxiety symptom scores** | 0.7063927 | 1 | 0.4879264 |
|  | **Hostility scores** | 0.5716755 | 0.4879264 | 1 |
| Girls | **Depressive symptom scores** | 1 | 0.7080862 | 0.6319653 |
|  | **Anxiety symptom scores** | 0.7080862 | 1 | 0.5330634 |
|  | **Hostility scores** | 0.6319653 | 0.5330634 | 1 |
| Boys | **Depressive symptom scores** | 1 | 0.6208111 | 0.4962394 |
|  | **Anxiety symptom scores** | 0.6208111 | 1 | 0.4297145 |
|  | **Hostility scores** | 0.4962394 | 0.4297145 | 1 |

# SUPPLEMENTARY FIGURES


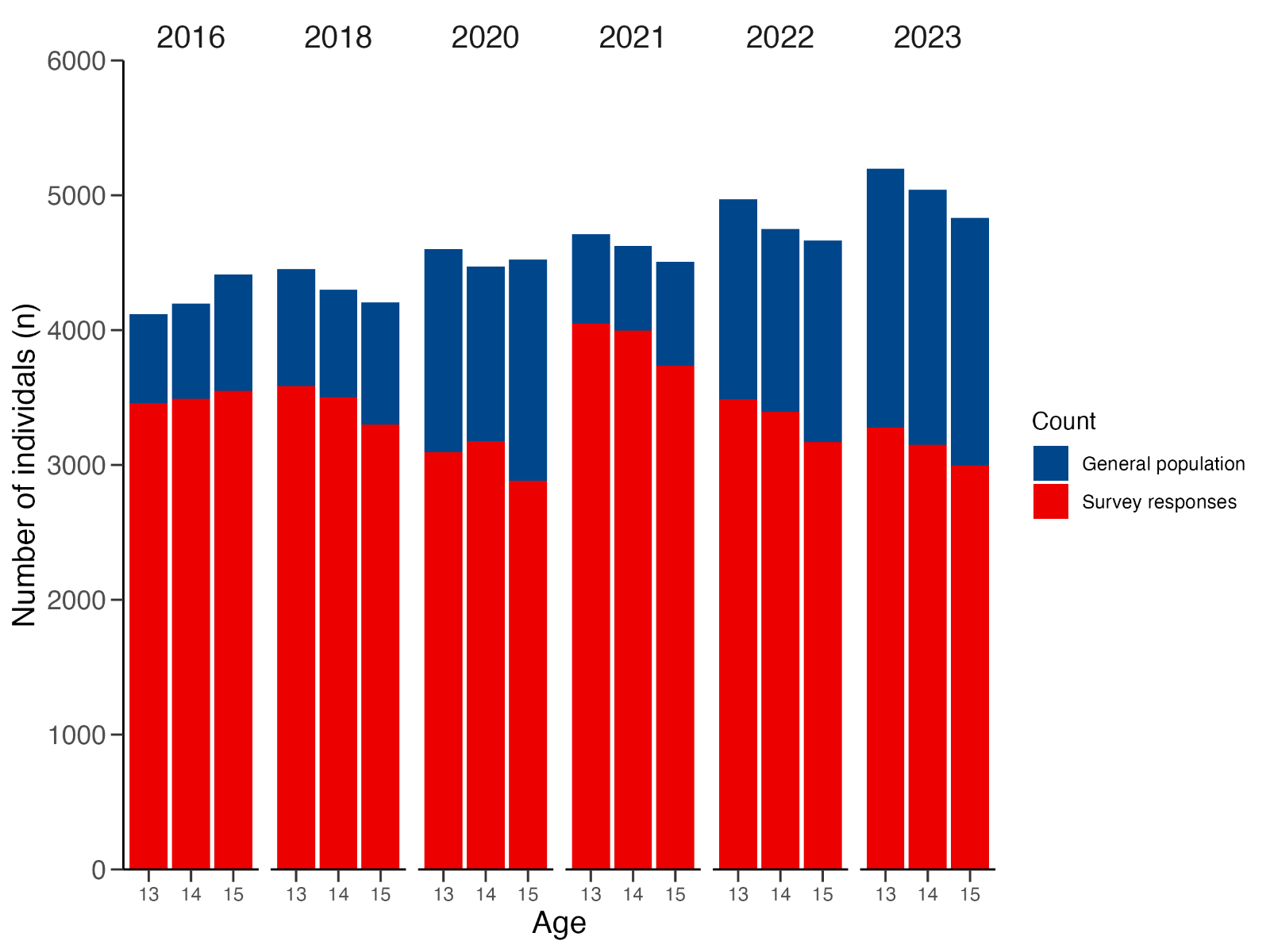


Figure S1. Survey response rates (red bars) for each age group, across the study period (2016 - 2023). Total population counts are shown in blue using demographic data from Statistics Iceland.

# R-script

R script to accompany the paper “Bioecological approach towards predicting adolescent mental health during COVID-19 in Iceland (2016-2023): a repeated, cross-sectional, population-based study”

This script details a subset of the analyses used within the study, namely:

- Converting composite survey scores to a beta-scale (and reversing this for model estimates later)
- Performing beta-distribution mixed-effects models with glmmTMB
- Model building and stepwise reduction with buildmer
- Calculating high-threshold mental health scores benchmarked to 2016 values
- Mixed-effects logistic regression for binary variables

##

## Create a dummy dataset modelled on the summary statistics of variables from Youth in Iceland study

Note that while the overall distributions are modelled on the original data the dummy data does not replicate the findings.

# Install the necessary package if not already installed
if (!require(PearsonDS)) install.packages("PearsonDS", dependencies=TRUE)

# Load the package
library(PearsonDS)

#Create variable for sum scores that matches distribution statistics in the Youth in Iceland study
# Depression - Define target statistics
dep_sx_mean <- 18.50402
dep_sx_sd <- 7.53174
dep_sx_skew <- 0.9223757
dep_sx_kurt <- 3.053184
dep_sx_min <- 10
dep_sx_max <- 40
sample_size <- 10000 # Adjust the sample size as needed
# Generate the Pearson Type IV distributed data
dep_sx <- rpearson(n = sample_size, moments = c(dep_sx_mean, dep_sx_sd, dep_sx_skew, dep_sx_kurt))
# Scale to fit within the 10-40 range and round to nearest integer
dep_sx <- round(dep_sx)
# Ensure values are within the desired bounds
dep_sx <- ifelse(dep_sx < dep_sx_min, dep_sx_min, dep_sx)
dep_sx <- ifelse(dep_sx > dep_sx_max, dep_sx_max, dep_sx)

# Anxiety - Define target statistics
anx_sx_mean <- 10.01877
anx_sx_sd <- 3.838777
anx_sx_skew <- 0.6281045
anx_sx_kurt <- 2.588421
anx_sx_min <- 5
anx_sx_max <- 20
anx_sx <- rpearson(n = sample_size, moments = c(anx_sx_mean, anx_sx_sd, anx_sx_skew, anx_sx_kurt))
anx_sx <- round(anx_sx)
# Ensure values are within the desired bounds
anx_sx <- ifelse(anx_sx < anx_sx_min, anx_sx_min, anx_sx)
anx_sx <- ifelse(anx_sx > anx_sx_max, anx_sx_max, anx_sx)

#Anger - Define target statistics
anger_sx_mean <- 8.686507
anger_sx_sd <- 3.6184
anger_sx_skew <- 1.196271
anger_sx_kurt <- 3.873987
anger_sx_min <- 5
anger_sx_max <- 20
anger_sx <- rpearson(n = sample_size, moments = c(anger_sx_mean, anger_sx_sd, anger_sx_skew, anger_sx_kurt))
anger_sx <- round(anger_sx)
# Ensure values are within the desired bounds
anger_sx <- ifelse(anger_sx < anger_sx_min, anger_sx_min, anger_sx)
anger_sx <- ifelse(anger_sx > anger_sx_max, anger_sx_max, anger_sx)

# Generating screen variables with specified values and ensuring they are integers
screen_video <- sample(c(0, 0.5, 1, 2, 3, 4, 5, 6), sample_size, replace = TRUE)
screen_gamealone <- sample(c(0, 0.5, 1, 2, 3, 4, 5, 6), sample_size, replace = TRUE)
screen_gamefriend <- sample(c(0, 0.5, 1, 2, 3, 4, 5, 6), sample_size, replace = TRUE)
screen_socialm <- sample(c(0, 0.5, 1, 2, 3, 4, 5, 6), sample_size, replace = TRUE)
screen_offline <- sample(c(0, 0.5, 1, 2, 3, 4, 5, 6), sample_size, replace = TRUE)

# Creating the screen_sx variable as the sum of all screen variables
screen_sx <- screen_video + screen_gamealone + screen_gamefriend + screen_socialm + screen_offline

# Generating other variables as before (assuming these are generated similarly)
year <- sample(2016:2023, sample_size, replace = TRUE)
gender <- sample(c("female", "male"), sample_size, replace = TRUE)
age <- sample(13:15, sample_size, replace = TRUE)
school <- sample(1:50, sample_size, replace = TRUE) # Creating a list of 1 to X schools
language <- sample(1:2, sample_size, replace = TRUE)
residency <- sample(0:1, sample_size, replace = TRUE)
parsup_sx <- sample(5:20, sample_size, replace = TRUE)

# Additional binary variables (with NA values if year == 2021)
set_na <- year == 2021
sick_30 <- ifelse(set_na, NA, rbinom(sample_size, 1, 0.054))
fight_30 <- ifelse(set_na, NA, rbinom(sample_size, 1, 0.095))
grade_30 <- ifelse(set_na, NA, rbinom(sample_size, 1, 0.204))
rom_30 <- ifelse(set_na, NA, rbinom(sample_size, 1, 0.044))
par_fight_30 <- ifelse(set_na, NA, rbinom(sample_size, 1, 0.047))
employ_year <- ifelse(set_na, NA, rbinom(sample_size, 1, 0.041))
divorce_year <- ifelse(set_na, NA, rbinom(sample_size, 1, 0.021))
witness_domestic_year <- ifelse(set_na, NA, rbinom(sample_size, 1, 0.013))
domestic_year <- ifelse(set_na, NA, rbinom(sample_size, 1, 0.012))
par_death_year <- ifelse(set_na, NA, rbinom(sample_size, 1, 0.008))
f_death_year <- ifelse(set_na, NA, rbinom(sample_size, 1, 0.027))
adult_sex_year <- ifelse(set_na, NA, rbinom(sample_size, 1, 0.01))
peer_sex_year <- ifelse(set_na, NA, rbinom(sample_size, 1, 0.021))
accident_year <- ifelse(set_na, NA, rbinom(sample_size, 1, 0.061))

# Create the dummy dataset
dummy_data <- data.frame(
 YEAR = as.factor(year),
 gender = as.factor(gender),
 age = as.factor(age),
 school = as.factor(school),
 language = language,
 residency = residency,
 parental_support = parsup_sx,
 depression_sum = dep_sx,
 anxiety_sum = anx_sx,
 anger_sum = anger_sx,
 wellbeing_sum = wellbeing_sx,
 sick_30 = sick_30,
 fight_30 = fight_30,
 grade_30 = grade_30,
 rom_30 = rom_30,
 par_fight_30 = par_fight_30,
 employ_year = employ_year,
 divorce_year = divorce_year,
 witness_domestic_year = witness_domestic_year,
 domestic_year = domestic_year,
 par_death_year = par_death_year,
 f_death_year = f_death_year,
 adult_sex_year = adult_sex_year,
 peer_sex_year = peer_sex_year,
 accident_year = accident_year,
 screen_video = screen_video,
 screen_gamealone = screen_gamealone,
 screen_gamefriend = screen_gamefriend,
 screen_socialm = screen_socialm,
 screen_offline_recode = screen_offline,
 screen_sx = screen_sx
)

# Verify the summary of the data
summary(dummy_data)

##

## Cumulative survey score data analysis (‘base’ models in paper)

### Step 1: Converting cumulative/composite mental health survey scores to a beta scale for statistical analysis

**Explanation**: Sum scores from likert scale data for mental health outcomes are typically treated as continuous. However, in many cases assumptions on normally distributed data are not met in mental health data (data points from survey data are often skewed (*e.g.* most people do not report feelings of suicidality)), scores are double-bound (constrained by number of questions and response outcomes), and exclusively positive integer scales (no negative values). In this case a beta-distributed model tends to be appropriate (validated with distribution analysis), but requires data to be transformed to a 0-1 scale.

#### Step 1.1: Check distribution of data points within cumulative survey score variables

#Check distribution
if (!require(fitdistrplus)) install.packages("fitdistrplus", dependencies=TRUE)

library(fitdistrplus)
descdist(as.numeric(dummy_data$depression_sum))


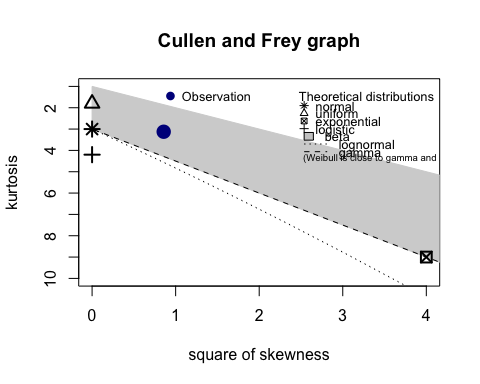


## summary statistics
## ------
## min: 15 max: 28
## median: 18
## mean: 18.5093
## estimated sd: 2.785191
## estimated skewness: 0.9256989
## estimated kurtosis: 3.125261

#### Step 1.2: Convert score values to a scale between 0-1

See: <https://github.com/glmmTMB/glmmTMB/issues/507>

Following protocol in “A better lemon squeezer?” Smithson & Verkuilen 2006 (DOI 10.1037/1082-989X.11.1.54).

See also Geissinger et al 2022 (10.1002/ecs2.3940) and Verkuilen & Smithson 2012 (10.3102/1076998610396895)

# a) generate variable statistics
N <- length(dummy_data$depression_sum)
 # Define the minimum and maximum possible scores for the variable sum score
a <- min(dummy_data$depression_sum)
b <- max(dummy_data$depression_sum)

# b) Linear transformation to open unit interval (0, 1)
y_prime <- (dummy_data$depression_sum - a) / (b - a)

# c) Compression to avoid zeros and ones
y_double_prime <- (y_prime * (N - 1) + 1/2) / N
 #Compare new values to previous

#d) Rename variable
dummy_data$depression_beta <- y_double_prime
plot(dummy_data$depression_beta, dummy_data$depression_sum)


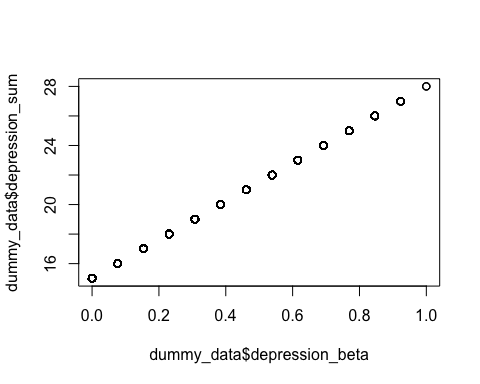


# Option - create gender stratified datasets. Worth doing if you intend to use emmeans package, which will not work if you using the subset function (e.g. data=subset(dummy_data, gender =="male") within the glmmTMB models).
dummy_data_f <- subset(dummy_data, gender=="female")
dummy_data_m <- subset(dummy_data, gender=="male")

# Repeat as necessary for other variables that comprise of a cumulative sum score of survey questions

###

### Step 2: Perform beta mixed-effects regression for mental health composite scores (referred to as ‘base models’ in the paper)

if (!require(glmmTMB)) install.packages("glmmTMB", dependencies=TRUE)

library(glmmTMB)

# Using the glmmTMB package to specify beta_family distribution, fixed and random variables
dep_glm = glmmTMB(depression_beta ~ YEAR + age + gender + (1|school) + (1|residency), family = beta_family(), data=dummy_data)
dep_glm_f = glmmTMB(depression_beta ~ YEAR + age + (1|school) + (1|residency), family = beta_family(), data=dummy_data_f)
dep_glm_m = glmmTMB(depression_beta ~ YEAR + age + (1|school) + (1|residency), family = beta_family(), data=dummy_data_m)

summary(dep_glm)

## Family: beta ( logit )
## Formula:
## depression_beta ~ YEAR + age + gender + (1 | school) + (1 | residency)
## Data: dummy_data
##
## AIC BIC logLik deviance df.resid
## -8348.0 -8239.9 4189.0 -8378.0 9985
##
## Random effects:
##
## Conditional model:
## Groups Name Variance Std.Dev.
## school (Intercept) 0.0068209 0.08259
## residency (Intercept) 0.0003636 0.01907
## Number of obs: 10000, groups: school, 50; residency, 2
##
## Dispersion parameter for beta family (): 2.51
##
## Conditional model:
## Estimate Std. Error z value Pr(>|z|)
## (Intercept) -1.10611 0.04127 -26.800 < 2e-16 ***
## YEAR2017 0.07979 0.04258 1.874 0.06095 .
## YEAR2018 0.08380 0.04220 1.986 0.04705 *
## YEAR2019 0.06059 0.04283 1.415 0.15718
## YEAR2020 0.08321 0.04237 1.964 0.04954 *
## YEAR2021 0.06668 0.04252 1.568 0.11688
## YEAR2022 0.11902 0.04235 2.810 0.00495 **
## YEAR2023 0.07733 0.04212 1.836 0.06634 .
## age14 -0.02363 0.02574 -0.918 0.35855
## age15 0.01758 0.02589 0.679 0.49707
## gendermale -0.01330 0.02583 -0.515 0.60657
## genderother -0.01910 0.02572 -0.743 0.45755
## ---
## Signif. codes: 0 '***' 0.001 '**' 0.01 '*' 0.05 '.' 0.1 ' ' 1

#Useful data exploration – note emmeans doesn’t seem to work if you use subset function in the model building step

#comparing years
if (!require(emmeans)) install.packages("emmeans", dependencies=TRUE)

library(emmeans)

dep_year <- emmeans(dep_glm, ~ YEAR)
pairwise_comparisons <- pairs(dep_year)
# summary(pairwise_comparisons)

if (!require(sjPlot)) install.packages("sjPlot", dependencies=TRUE)

library(sjPlot)

plot_model(dep_glm, vline.color = "black",
 show.intercept = TRUE, show.values=TRUE, value.offset = 0.4, transform=NULL,
 title="All: Depression score model, basic predictors")


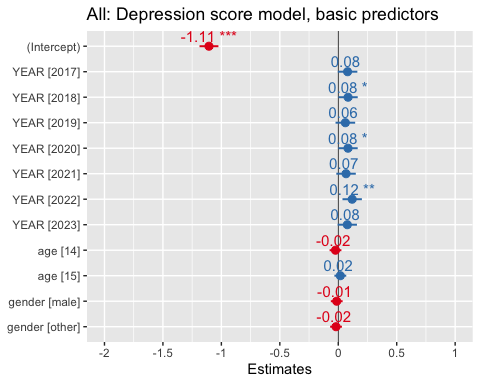


# Model tables for comparisons
dep_models <- list(dep_glm, dep_glm_f, dep_glm_m)
dep_model_table <- tab_model(dep_models,
 dv.labels= c("All genders", "Female", "Male"),
 show.fstat=TRUE, show.aic=TRUE, show.se=TRUE, transform=NULL,
 title="Summary table for basic variable effect on depression score from glmmTMB beta models")
 #file = "dep_models.html") #add this to export table, check getwd()

# Repeat as necessary for other variables that comprise of a sum score of survey questions

###

### Step 3: Convert model estimates back to original survey score scale

Can be useful to convert the model estimates back to the original values of the composite score of mental health from the survey scale.

#### Step 3.1 - Define characteristics from original survey scores (not the beta transformed values)

N <- length(dummy_data$depression_sum)
#if you created separate data sets for the genders you'll need to specify -
#N_fem <- length(dummy_data_f$depression_sum)
a <- min(dummy_data$depression_sum)
b <- max(dummy_data$depression_sum)

#### Step 3.2 - Extract the individual model estimates (ME) and intercept from the models

#a) all genders
dep_glm_summary <- summary(dep_glm) #to check against
rownames(dep_glm_summary$coefficients$cond)

## [1] "(Intercept)" "YEAR2017" "YEAR2018" "YEAR2019" "YEAR2020"
## [6] "YEAR2021" "YEAR2022" "YEAR2023" "age14" "age15"
## [11] "gendermale" "genderother"

dep_ME <- as.numeric(unlist(fixef(dep_glm)))
dep_intercept <- dep_ME[1]
# repeat for gender-stratified models if required

#### Step 3.3 - Calculate the real-scale value for the intercept (RSI)

#a) all genders
dep_I <- exp(dep_intercept) / (1 + exp(dep_intercept)) #inverse log (undo model logit transformation)
dep_RSI <- (dep_I * N - 1/2) / (N - 1) * (b - a) + a
# repeat for gender-stratified models if required

#### Step 3.4 - Calculate real-scale values for the model estimates

#a) all genders
#Identify number of estimates/variables in the model - use this to define the numeric value in step below
length(dep_ME)

## [1] 13

#Calculate overall scores - Define model estimates starting 2 (avoiding the intercept) and SUBTRACT 1 (e.g. if length(dep_ME)=13, specify 2:12), add to the intercept to give overall score (instead of change relative to the intercept)
dep_EC <- dep_ME[2:12] + dep_intercept
dep_TE <- exp(dep_EC) / (1 + exp(dep_EC)) #inverse log (undo model logit transformation)
dep_BTE <- (dep_TE * N - 1/2) / (N - 1) * (b - a) + a #overall score
dep_change_percent <- round(((dep_BTE - dep_RSI) / dep_RSI) * 100, 3) # % change relative to the real-scale intercept

# repeat for gender-stratified models if required

#### Step 3.5 - Create result table to show: Variable name, original model estimates, real scale value and % change in scores relative to the intercept value

#a) all genders
#Generate variable names
dep_glm_variables <- rownames(dep_glm_summary$coefficients$cond)
dep_glm_variables <- dep_glm_variables[dep_glm_variables != "(Intercept)"]

dep_real_scale_table <- cbind(dep_glm_variables, round(dep_ME[2:12], 3), round(dep_BTE, 3), dep_change_percent) #ensure this matches numbers defined in step 3.4
colnames(dep_real_scale_table) <- c("Variable name", "Beta-model estimate", "Real scale value", "Real scale % change")
print(dep_real_scale_table)

## Variable name Beta-model estimate Real scale value Real scale % change
## [1,] "YEAR2017" "0.08" "18.429" "1.084"
## [2,] "YEAR2018" "0.084" "18.439" "1.14"
## [3,] "YEAR2019" "0.061" "18.381" "0.819"
## [4,] "YEAR2020" "0.083" "18.438" "1.131"
## [5,] "YEAR2021" "0.067" "18.396" "0.903"
## [6,] "YEAR2022" "0.119" "18.529" "1.632"
## [7,] "YEAR2023" "0.077" "18.423" "1.05"
## [8,] "age14" "-0.024" "18.174" "-0.313"
## [9,] "age15" "0.018" "18.274" "0.235"
## [10,] "gendermale" "-0.013" "18.199" "-0.177"
## [11,] "genderother" "-0.019" "18.185" "-0.253"

round(dep_RSI,3) #Real scale intercept for comparison

## [1] 18.231

#Repeat as necessary for gender-stratified models and other dependent variable models

#If you want log odds ratios for the model estimates (effect size) then simply exponentiate the raw model beta model estimates: e.g. exp(dep_ME) (dep_ME defined in step 3.2)

##

## Model building and stepwise reduction (as for the ‘bioecological models’ in the paper)

### Step 1: Define starting model formula

Include all pre-specified, hypothesis-driven model covariates, and any interaction and random terms.

whole_dep_all_ff <- depression_beta ~
 YEAR + YEAR*age + YEAR*gender +
 YEAR*parsup_sx + YEAR*language +
 YEAR*screen_video + YEAR*screen_gamealone + YEAR*screen_offline + YEAR*screen_gamefriend +YEAR*screen_socialm +
 YEAR*sick_30 +YEAR*fight_30 +YEAR*par_fight_30 +YEAR*grade_30 + YEAR*rom_30 +
 YEAR*accident_year +YEAR*employ_year +YEAR*divorce_year +
 YEAR*witness_domestic_year +YEAR*domestic_year +YEAR*par_death_year +YEAR*adult_sex_year + +
 YEAR*peer_sex_year +YEAR*f_death_year +
 (1|school) + (1|residency)

#Repeat for other dependent variables or gendered datasets (remove the gender variable if needed)

###

### Step 2: Use buildglmmTMB to build a formula using all the terms above, and order them to make the model that describes the data the best (log likelihood, LRT), and avoiding non-convergence

if (!require(buildmer)) install.packages("buildmer", dependencies=TRUE)

library(buildmer)

#This can take some time depending on the size of your dataset
whole_dep_all_build <- buildglmmTMB(whole_dep_all_ff, family = beta_family(), data=dummy_data, buildmerControl = buildmerControl(direction="order"))

## Determining predictor order

## Fitting via glmmTMB, with ML: depression_beta ~ 1

## Currently evaluating LRT for: YEAR, age, gender, parsup_sx, language,
## screen_video, screen_gamealone, screen_offline, screen_gamefriend,
## screen_socialm, sick_30, fight_30, par_fight_30, grade_30, rom_30,
## accident_year, employ_year, divorce_year, witness_domestic_year,
## domestic_year, par_death_year, adult_sex_year, peer_sex_year,
## f_death_year

## Fitting via glmmTMB, with ML: depression_beta ~ 1 + YEAR

## Fitting via glmmTMB, with ML: depression_beta ~ 1 + age

[output truncated]

#Step 2.1 : Specify the formula of the best model from output above:
(whole_dep_all_form <- formula(whole_dep_all_build@model))

### Step 3: Use build glmmTMB to refine the model based on the BIC criterion, in a backwards direction, removing terms from the tail end of the formula first.

#You can change the criteria, e.g. to AIC to make it less stringent. This step can also take some time.
whole_dep_all_step <- buildglmmTMB(whole_dep_all_form, buildmerControl = buildmerControl(family = beta_family(), data=dummy_data, direction="backward", crit="BIC", elim="BIC"))

## Fitting ML and REML reference models

## Fitting via glmmTMB, with REML: depression_beta ~ 1 + parsup_sx +
## screen_video + language + age + YEAR + language:YEAR +
## parsup_sx:YEAR + screen_video:YEAR + screen_gamealone +
## YEAR:screen_gamealone + fight_30 + rom_30 + accident_year +
## witness_domestic_year + peer_sex_year + YEAR:peer_sex_year +
## employ_year + sick_30 + domestic_year + grade_30 + (1 | school) +
## (1 | residency)

## Fitting via glmmTMB, with ML: depression_beta ~ 1 + parsup_sx +
## screen_video + language + age + YEAR + language:YEAR +
## parsup_sx:YEAR + screen_video:YEAR + screen_gamealone +
## YEAR:screen_gamealone + fight_30 + rom_30 + accident_year +
## witness_domestic_year + peer_sex_year + YEAR:peer_sex_year +
## employ_year + sick_30 + domestic_year + grade_30 + (1 | school) +
## (1 | residency)

## Convergence failure. Reducing terms and retrying... The failures were:
## glmmTMB reports nonconvergence (1)

[output truncated]

#Step 3.1: Specify the formula or the best model from the output above:
(whole_dep_all_red <- formula(whole_dep_all_step@model))

###

### Step 4: Run the model using the resulting formula on the resulting formula

whole_dep_all_glm <- glmmTMB(whole_dep_all_red, family=beta_family, data=dummy_data)

###

### Step 5: Examine output

summary(whole_dep_all_glm)

## Family: beta ( logit )
## Formula: depression_beta ~ 1
## Data: dummy_data
##
## AIC BIC logLik deviance df.resid
## -8340.8 -8326.3 4172.4 -8344.8 9998
##
##
## Dispersion parameter for beta family (): 2.49
##
## Conditional model:
## Estimate Std. Error z value Pr(>|z|)
## (Intercept) -1.04611 0.01182 -88.53 <2e-16 ***
## ---
## Signif. codes: 0 '***' 0.001 '**' 0.01 '*' 0.05 '.' 0.1 ' ' 1

#Alternative packages for running such models applying Markov chain Monte Carlo (MCMC) algorithms are available in the brms and MCMCglmm packages. It is worth comparing models between methods, the MCMC methods tend to be more computationally intense, and possibly not practical for large sample sizes and complex models.

##

## High threshold mental health score data analysis

### Step 1: Calculate pre-COVID 2016 baseline mental health cumulative scores: what is the top 5% threshold for each respective age and gender grouping?

Create new data frames for each gender/age group in 2016 (or baseline year)

if (!require(tidyverse)) install.packages("tidyverse", dependencies=TRUE)

library(tidyverse)
DD_2016 <- dummy_data %>% filter(YEAR == 2016)
DD_2016f13 <- DD_2016 %>% filter(age == 13, gender=="female")
DD_2016f14 <- DD_2016 %>% filter(age == 14, gender=="female")
DD_2016f15 <- DD_2016 %>% filter(age == 15, gender=="female")
DD_2016m13 <- DD_2016 %>% filter(age == 13, gender=="male")
DD_2016m14 <- DD_2016 %>% filter(age == 14, gender=="male")
DD_2016m15 <- DD_2016 %>% filter(age == 15, gender=="male")

###

### Step 2: Calculate the number of individuals reporting the highest scores >95% within the reference year, for each age and gender grouping

# Calculate the 95th percentile threshold for the cumulative depression (depression_sum) variable
dep_95_f13 <- quantile(DD_2016f13$depression_sum, 0.95)
dep_95_f14 <- quantile(DD_2016f14$depression_sum, 0.95)
dep_95_f15 <- quantile(DD_2016f15$depression_sum, 0.95)
dep_95_m13 <- quantile(DD_2016m13$depression_sum, 0.95)
dep_95_m14 <- quantile(DD_2016m14$depression_sum, 0.95)
dep_95_m15 <- quantile(DD_2016m15$depression_sum, 0.95)

#calculate % per year, within gender and age categories
#female 13
dep_95_f13_counts <- subset(dummy_data_f, age == 13) %>%
 group_by(YEAR, gender, age) %>%
 summarize(
 count_above_threshold = sum(depression_sum > dep_95_f13),
 total_count = n(),
 percentage_above_threshold = 100 * count_above_threshold / total_count
 )

## `summarise()` has grouped output by 'YEAR', 'gender'. You can override using
## the `.groups` argument.

#female 14
dep_95_f14_counts <- subset(dummy_data_f, age == 14) %>%
 group_by(YEAR, gender, age) %>%
 summarize(
 count_above_threshold = sum(depression_sum > dep_95_f14),
 total_count = n(),
 percentage_above_threshold = 100 * count_above_threshold / total_count
 )

## `summarise()` has grouped output by 'YEAR', 'gender'. You can override using
## the `.groups` argument.

#Repeat as necessary for each age/gender combination at baseline

#Merge to single dataframe
threshold_dfs <- list(dep_95_f13_counts, dep_95_f14_counts) #add further as necessary
threshold_df <- bind_rows(threshold_dfs)
threshold_df <- as.data.frame(threshold_df)

#### Step 2.1 Create plot

if (!requireNamespace("ggplot2", quietly = TRUE)) install.packages("ggplot2", dependencies = TRUE)
if (!requireNamespace("ggsci", quietly = TRUE)) install.packages("ggsci", dependencies = TRUE)
library(ggplot2)
library(ggsci)

dep_threshold_plot <- ggplot(threshold_df, aes(x=YEAR, y=percentage_above_threshold, group=age, fill=age)) +
 geom_col(position=position_dodge())+
 labs(x = "Survey Year", y = "Participants with high \n depression symptom scores (%)", fill="Age")+
 scale_y_continuous(expand=c(0,0.1), limits=c(0,14), breaks=c(0, 2, 4, 6, 8, 10, 12, 14)) +
 scale_fill_lancet()+
 theme_bw()
# Facet by gender to present two graphs adjacently
# dep_threshold_plot <- dep_threshold_plot + facet_grid(.~ gender, labeller= labeller(gender = c("female"="Female", "male"="Male")))
dep_threshold_plot


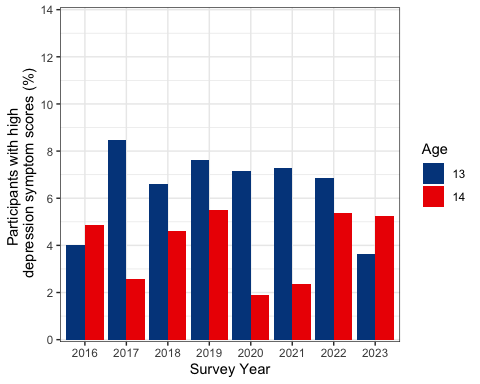


### Step 3: Create binary variable (1= above 95th threshold, 0 = below 95th threshold) to determine proportion of individuals reporting extreme >95th centile mental health scores

#Create new binary variable for the top 5% scores
dummy_data$deptop5 <- 0

# Assigning 0 or 1 values to deptop5 based on the respective thresholds
dummy_data$deptop5[dummy_data$gender == 'female' & dummy_data$age == 13 & dummy_data$depression_sum > dep_95_f13] <- 1
dummy_data$deptop5[dummy_data$gender == 'female' & dummy_data$age == 14 & dummy_data$depression_sum > dep_95_f14] <- 1
dummy_data$deptop5[dummy_data$gender == 'female' & dummy_data$age == 15 & dummy_data$depression_sum > dep_95_f15] <- 1
#... continue for other age/gender groupings

###

### Step 4: Test for changes with binomial mixed effect models

deptop5glm <- glmmTMB(deptop5 ~ YEAR * age * gender + (1|school) + (1|residency), data=dummy_data, family = binomial(link=logit))
# Female
deptop5glm_f <- glmmTMB(deptop5 ~ YEAR * age + (1|school) + (1|residency), data=subset(dummy_data, gender=="female"), family = binomial(link=logit))
summary(deptop5glm_f) #NOTE that the model doesn't converge with the artificial/dummy data. The model still runs but the output is not complete.

## Family: binomial ( logit )
## Formula: deptop5 ~ YEAR * age + (1 | school) + (1 | residency)
## Data: subset(dummy_data, gender == "female")
##
## AIC BIC logLik deviance df.resid
## 1536.1 1695.3 -742.1 1484.1 3350
##
## Random effects:
##
## Conditional model:
## Groups Name Variance Std.Dev.
## school (Intercept) 1.693e-08 1.301e-04
## residency (Intercept) 2.528e-19 5.028e-10
## Number of obs: 3376, groups: school, 50; residency, 2
##
## Conditional model:
## Estimate Std. Error z value Pr(>|z|)
## (Intercept) -3.17108 NaN NaN NaN
## YEAR2017 0.78845 NaN NaN NaN
## YEAR2018 0.52412 NaN NaN NaN
## YEAR2019 0.67787 NaN NaN NaN
## YEAR2020 0.60613 NaN NaN NaN
## YEAR2021 0.62948 NaN NaN NaN
## YEAR2022 0.56101 NaN NaN NaN
## YEAR2023 -0.10228 NaN NaN NaN
## age14 0.19701 NaN NaN NaN
## age15 -0.01390 NaN NaN NaN
## YEAR2017:age14 -1.44315 NaN NaN NaN
## YEAR2018:age14 -0.57857 NaN NaN NaN
## YEAR2019:age14 -0.54539 NaN NaN NaN
## YEAR2020:age14 -1.57686 NaN NaN NaN
## YEAR2021:age14 -1.38109 NaN NaN NaN
## YEAR2022:age14 -0.45861 NaN NaN NaN
## YEAR2023:age14 0.18599 NaN NaN NaN
## YEAR2017:age15 -0.25043 NaN NaN NaN
## YEAR2018:age15 -0.06189 NaN NaN NaN
## YEAR2019:age15 0.09282 NaN NaN NaN
## YEAR2020:age15 0.12785 NaN NaN NaN
## YEAR2021:age15 0.28417 NaN NaN NaN
## YEAR2022:age15 0.26167 NaN NaN NaN
## YEAR2023:age15 0.73640 NaN NaN NaN

#... continue for other age/gender groupings

# REFERENCES

1 Derogatis, Leonard R, Unger, Rachael. Symptom Checklist‐90‐Revised - Derogatis - Major Reference Works - Wiley Online Library. *Corsini Encycl Psychol* 2010; : 1–2.

2 Sigfusdottir ID, Asgeirsdottir BB, Sigurdsson JF, Gudjonsson GH. Trends in depressive symptoms, anxiety symptoms and visits to healthcare specialists: A national study among Icelandic adolescents. *Scand J Public Health* 2008; **36**: 361–8.

3 Rytilä-Manninen M, Fröjd S, Haravuori H, *et al.* Psychometric properties of the Symptom Checklist-90 in adolescent psychiatric inpatients and age- and gender-matched community youth. *Child Adolesc Psychiatry Ment Health* 2016; **10**: 23.

4 Wiznitzer M, Verhulst FC, van den Brink W, *et al.* Detecting psychopathology in young adults: the Young Adult Self Report, the General Health Questionnaire and the Symptom Checklist as screening instruments. *Acta Psychiatr Scand* 1992; **86**: 32–7.

5 Twenge JM, Martin GN, Campbell WK. Decreases in psychological well-being among American adolescents after 2012 and links to screen time during the rise of smartphone technology. *Emotion* 2018; **18**: 765–80.

6 Przybylski AK, Weinstein N. A Large-Scale Test of the Goldilocks Hypothesis: Quantifying the Relations Between Digital-Screen Use and the Mental Well-Being of Adolescents. *Psychol Sci* 2017; **28**: 204–15.

7 Gudjonsson G, Sigurdsson JF, Sigfusdottir ID. False confessions among 15- and 16-year-olds in compulsory education and the relationship with adverse life events. *J Forensic Psychiatry Psychol* 2009; **20**: 950–63.

8 Delignette-Muller ML, Dutang C. fitdistrplus: An R Package for Fitting Distributions. *J Stat Softw* 2015; **64**: 1–34.

9 Smithson M, Verkuilen J. A Better Lemon Squeezer? Maximum-Likelihood Regression With Beta-Distributed Dependent Variables. *Psychol Methods* 2006; **11**: 54–71.

10 Verkuilen J, Smithson M. Mixed and Mixture Regression Models for Continuous Bounded Responses Using the Beta Distribution. *J Educ Behav Stat* 2012; **37**: 82–113.

11 Liu F, Eugenio EC. A review and comparison of Bayesian and likelihood-based inferences in beta regression and zero-or-one-inflated beta regression. *Stat Methods Med Res* 2018; **27**: 1024–44.

12 Steyerberg EW. Clinical prediction models. Springer, 2019.

13 Harrell Jr FE. Hmisc: Harrell Miscellaneous. 2003; : 5.1-3.

14 Ravens-Sieberer U, Devine J, Napp A-K, *et al.* Three years into the pandemic: results of the longitudinal German COPSY study on youth mental health and health-related quality of life. *Front Public Health* 2023; **11**. https://www.frontiersin.org/journals/public-health/articles/10.3389/fpubh.2023.1129073 (accessed Feb 29, 2024).

15 Kaman A, Erhart M, Devine J, *et al.* Two Years of Pandemic: the Mental Health and Quality of Life of Children and Adolescents. *Dtsch Ärztebl Int* 2023; **120**: 269–70.

16 Shoshani A, Kor A. The longitudinal impact of the COVID-19 pandemic on adolescents’ internalizing symptoms, substance use, and digital media use. *Eur Child Adolesc Psychiatry* 2024; **33**: 1583–95.

17 Zijlmans J, Tieskens JM, van Oers HA, *et al.* The effects of COVID‐19 on child mental health: Biannual assessments up to April 2022 in a clinical and two general population samples. *JCPP Adv* 2023; **3**: e12150.

18 Barbieri V, Wiedermann CJ, Piccoliori G, *et al.* Evolution of Youth’s Mental Health and Quality of Life during the COVID-19 Pandemic in South Tyrol, Italy: Comparison of Two Representative Surveys. *Children* 2023; **10**: 895.

19 Barbieri V, Piccoliori G, Mahlknecht A, *et al.* Adolescent Mental Health during the COVID-19 Pandemic: The Interplay of Age, Gender, and Mental Health Outcomes in Two Consecutive Cross-Sectional Surveys in Northern Italy. *Behav Sci* 2023; **13**: 643.

20 Reiß F, Kaman A, Napp A-K, *et al.* Epidemiology of mental well-being in childhood and adolescence: results from three epidemiological studies before and during the COVID-19 pandemic. *Bundesgesundheitsblatt - Gesundheitsforschung - Gesundheitsschutz* 2023; **66**: 727–35.

21 Kiviruusu O, Ranta K, Lindgren M, *et al.* Mental health after the COVID-19 pandemic among Finnish youth: a repeated, cross-sectional, population-based study. *Lancet Psychiatry* 2024; **11**: 451–60.

22 van Oers HA, Alrouh H, Tieskens JM, *et al.* Changes in child and adolescent mental health across the COVID-19 pandemic (2018–2023): Insights from general population and clinical samples in the Netherlands. *JCPP Adv* 2024; **4**: e12213.

23 Kaman A, Devine J, Wirtz MA, *et al.* Trajectories of mental health in children and adolescents during the COVID-19 pandemic: findings from the longitudinal COPSY study. *Child Adolesc Psychiatry Ment Health* 2024; **18**: 89.

24 Park JL, McArthur BA, Plamondon A, *et al.* The course of children’s mental health symptoms during and beyond the COVID-19 pandemic. *Psychol Med* 2024; : 1–12.

25 Zoellner F, Erhart M, Napp A-K, *et al.* Risk and protective factors for mental health problems in children and adolescents during the COVID-19 pandemic: results of the longitudinal COPSY study. *Eur Child Adolesc Psychiatry* 2024; published online Oct 29. DOI:10.1007/s00787-024-02604-6.

26 Shoshani A. Longitudinal changes in children’s and adolescents’ mental health and well-being and associated protective factors during the COVID-19 pandemic. *Psychol Trauma Theory Res Pract Policy* 2024; **16**: 1158–68.

27 Bhandari N, Gupta S. Trends in Mental Wellbeing of US Children, 2019–2022: Erosion of Mental Health Continued in 2022. *Int J Environ Res Public Health* 2024; **21**: 132.
