# Supplementary material for: Adolescent mental health before, during, and after the COVID-19 pandemic in Iceland: a repeated, cross-sectional, population-based study
Source: Lancet Reg Health Eur. 2025 Apr 29;53:101301. doi: 10.1016/j.lanepe.2025.101301 (PMC12432985; doi:10.1016/j.lanepe.2025.101301)
Supplement: IcelandicAbstract [file mmc2.docx]

*This translation in Icelandic was submitted by the authors and we reproduce it as supplied. It has not been peer reviewed. Our editorial processes have only been applied to the original abstract in English, which should serve as reference for this manuscript.*

**Heildrænt líkan Bronfenbrenner notað til að spá fyrir um andlega líðan íslenskra ungmenna á landvísu á tímum COVID-19 faraldursins (2016-2023)**

**Ágrip**

**Bakgrunnur**

Andleg líðan ungmenna versnaði í COVID-19 faraldrinum. Fáar rannsóknir hafa kannað líðan ungmenna eftir að neyðarstigi vegna faraldursins var aflýst á heimsvísu (5. maí 2023). Þessi rannsókn kannaði breytingar á andlegri líðan ungmenna á Íslandi frá 2016 til 2023. Heildrænt líkan Bronfenbrenner sem samanstendur af líffræðilegum og víðtækum umhverfisþáttum var notað til að greina forspárþætti um andlega líðan hjá íslenskum ungmennum.

**Aðferð**

Notast var við þversniðsgögn úr Ungt fólk rannsóknum Rannsókna og greiningar. Borin voru saman svör úr spurningarkönnunum sem lagðar voru fyrir 13-15 ára ungmenni á landsvísu á árunum 2016, 2018, 2020, 2021, 2022 og 2023. Meðaltalssvörun ungmenna þessi ár var 75%. Þátttakendur svöruðu spurningum um þunglyndiseinkenni, kvíða, pirring (Symptom Checklist-90), stuðning frá foreldrum (Perceived Parental Support Scale), skjátíma og tíðni þess að verða fyrir streituvaldi og áfalli. Stigminnkandi slembiþáttarlíkön voru notuð til að meta tengsl aldurs, kyns, tíma og forspárþátta við andlega heilsu. Töpuð gildi voru reiknuð með marghliða tilreiknun og aðferð Bonferroni var notuð til að leiðrétta fyrir fjölda greininga.

**Niðurstöður**

Alls tóku 62.011 ungmenni þátt: 48,2% stelpur (n=29.890), 50,0% strákar (n=31.002) og 1,8% kynsegin (n=1.119). Þunglyndiseinkenni minnkuðu árið 2023 samanborið við 2021 (β=0,19, 95% ÖB=0,13 til 0,24), en voru enn hærri en fyrir faraldurinn (2016: β=-0,38, 95% ÖB=-0,44 til -0,33; 2018: β=-0,26, 95% ÖB=-0,31 til -0,20). Það var einnig aukning á kvíða og pirring sem viðhélst til ársins 2023 (kvíði: 2016 β -0.290, 95% CI –0.35 to –0.24, 2018 β -0.200, 95% CI –0.26 to –0.15; pirringur: 2016 β -0.260, 95% CI –0.31 to –0.20, 2018 β -0.120, 95% CI –0.18 to –0.07). Greiningar á heildræna líkani Bronfenbrenners leiddu í ljós að lítill stuðningur frá foreldrum, mikil samfélagsmiðlanotkun og slæmar einkunnir spáðu helst fyrir um verri andlega heilsu á tímum COVID-19.

**Ályktun**

COVID-19 faraldurinn hefur haft alvarleg og langvarandi slæm áhrif á andlega heilsu íslenskra ungmenna**.** Niðurstöðurnar benda til þess að markviss inngrip séu nauðsynleg til að þess að stemma stigu við aukinni vanlíðan ungmenna í kjölfar faraldursins**,** með sérstakri áherslu á að efla stuðning frá foreldrum og fræðslu um samfélagsmiðlanotkun.

**Styrkir**

Rannsóknarsjóður Rannís (217612-051); NordForsk (Styrkur: 147386).
